# Hyperphalangy in a new sinemydid turtle from the Early Cretaceous Jehol Biota

**DOI:** 10.7717/peerj.5371

**Published:** 2018-07-27

**Authors:** Shuai Shao, Lan Li, Yang Yang, Chang-Fu Zhou

**Affiliations:** 1 Research Center of Palaeontology and Stratigraphy, Jilin University, Changchun, Jilin, China; 2 Paleontological Institute, Shenyang Normal University, Shenyang, Liaoning, China; 3 School of Earth and Space Sciences, Peking University, Beijing, China; 4 Anhui Geological Museum, Hefei, Anhui, China; 5 College of Earth Science and Engineering, Shandong University of Science and Technology, Qingdao, Shandong, China

**Keywords:** Early Cretaceous, Sinemydidae, Jehol Biota, Hyperphalangy

## Abstract

Hyperphalangy is a rare condition in extant aquatic turtles, and mainly limited to soft-shelled turtles. Here we report a new freshwater turtle, *Jeholochelys lingyuanensis* gen. et sp. nov. from the Early Cretaceous Jehol Biota of western Liaoning, China. This new turtle is characterized by a hyperphalangy condition with one additional phalanx in pedal digit V, rather than the primitive condition (phalangeal formula: 2-3-3-3-3) of crown turtles. *J. lingyuanensis* is recovered with other coexisting turtles in the family Sinemydidae in the phylogenetic analysis. This discovery further confirms that hyperphalangy occurred multiple times in the early evolutionary history of the crown turtles. Hyperphalangy is possibly a homoplasy in *Jeholochelys* and the soft-shelled turtles to adapt to the aquatic environments.

## Introduction

Extant turtles of terrestrial, semi-aquatic, and aquatic habitats have evolved diverse morphologic adaptations for these environments that involves the shell, limb proportions, and the phalangeal count (Joyce & Gauthier, 2004; Renous et al., 2008). Terrestrial turtles (e.g., testudinids) have relatively short manus and pedes with reduced phalangeal count (Crumly & Sánchez-Villagra, 2004; Joyce & Gauthier, 2004). In contrast, aquatic turtles have elongate manus and pedes, and for example, highly elongate phalanges are present in marine turtles (chelonioids) and freshwater pig-nosed turtles (carettochelyids) (Joyce & Gauthier, 2004). On the other hand, freshwater soft-shelled turtles (trionychids) tend to have an increased phalangeal number, a condition termed as hyperphalangy (Kükenthal, 1889, 1893). Hyperphalangy is frequently present in aquatic tetrapods, and is well known in cetaceans, ichthyosaurs, and plesiosaurs, where the phalanges form a long flipper. Although hyperphalangy in soft-shelled turtles does not contribute to the formation of longer flippers, the additional phalanges could help to enlarge the paddle surface to facilitate aquatic mobility (Delfino, Fritz & Sánchez-Villagra, 2010). Hyperphalangy is rarely known in other turtle lineages (Delfino, Fritz & Sánchez-Villagra, 2010; De la Fuente & Fernández, 2011; Li, Joyce & Liu, 2015).

In this study, a new sinemydid turtle is described from the Early Cretaceous Jiufotang Formation of Sihedang, Lingyuan, western Liaoning, China. This turtle is characterized by a series of features including a phalangeal formula of 2-3-3-3-4 for the pes, as a contrast to the common condition of the 2-3-3-3-3 formula of crown turtles. The new taxon shows hyperphalangy with one additional phalanx in pedal digit V provides an opportunity for investigating the evolution of hyperphalangy in turtles.

## Materials and Methods

### Material

**Figure 1:**
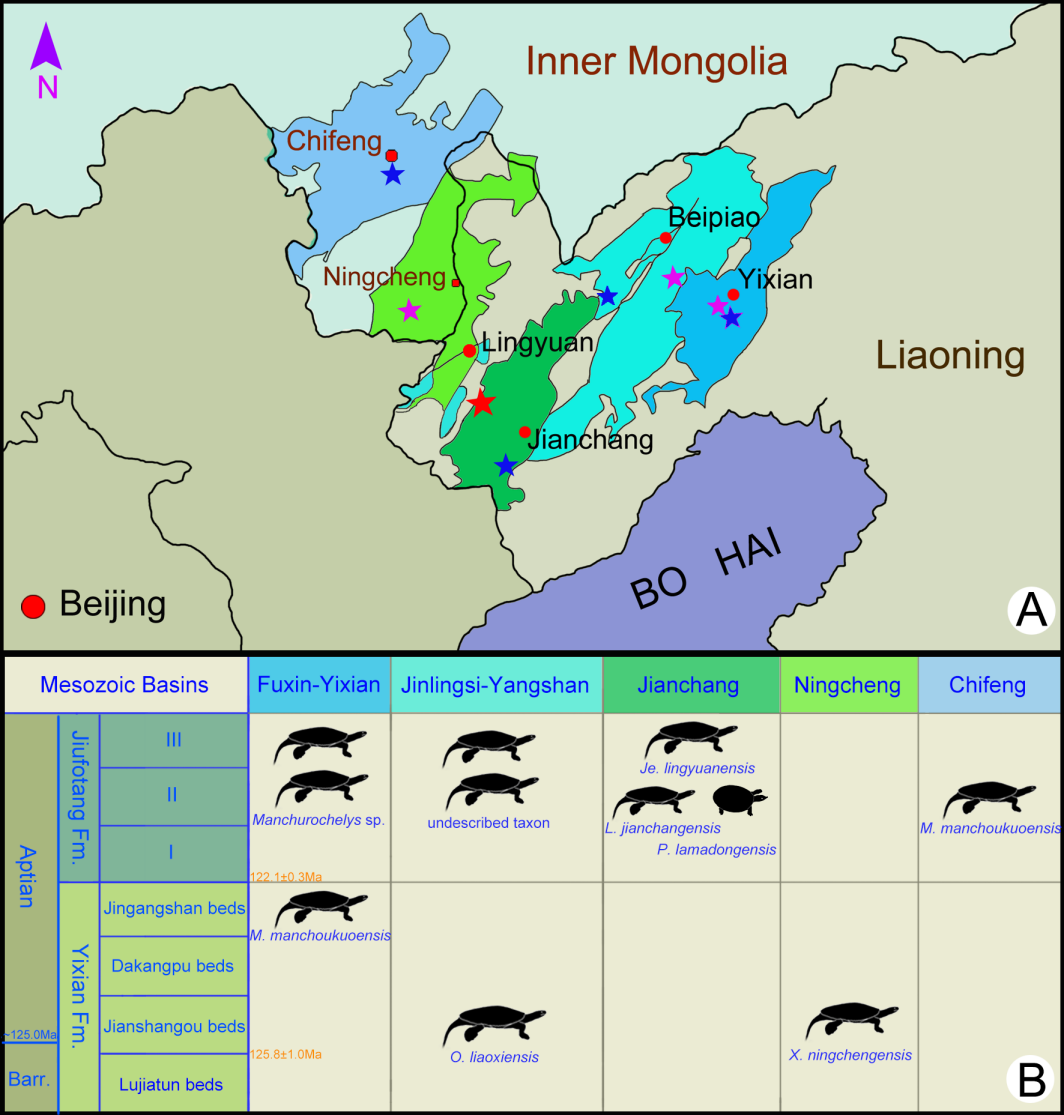
Fossil localities (A) and stratigraphic distribution (B) of turtles in the type areas of the Jehol Biota in northeastern China. The type locality of *Jeholochelys lingyuanensis* gen. et sp. nov. (red asterisk; 40°54′46″N; 119°29′45″E); other localities from Yixian Formation (purple asterisk) and Jiufotang Formation (blue asterisk) of Liaoning and Inner Mongolia. Major Mesozoic basins including Fuxin-Yixian Basin (dark blue), Jinlingsi-Yangshan Basin (blue green), Jianchang Basin (dark green), Ningcheng Basin (light green) and Chifeng Basin (light blue). Cities and counties marked by red circles. Geological dating from Chang et al. (2009) and Chang et al. (2017). Modified from Zhou & Rabi (2015).

**Figure 2:**
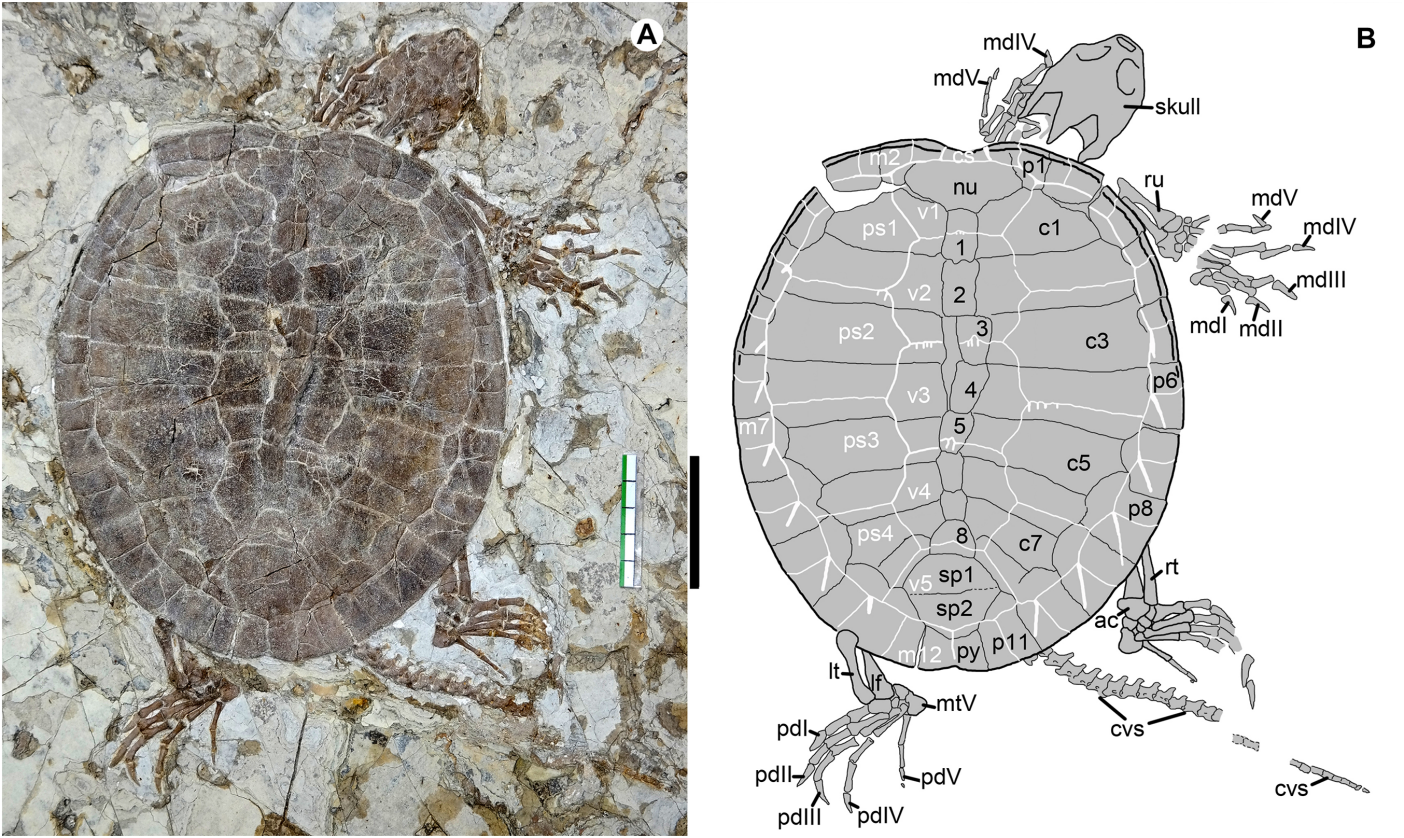
*Jeholochelys lingyuanensis* gen. et sp. nov. (Holotype, PMOL-AR00211; A and B, in dorsal view) from the Early Cretaceous Jiufotang Formation of Sihedang, Lingyuan, western Liaoning, China. Study sites: ac, astragalocalcaneum; cs, cervical scale; cvs, caudal vertebra series; c1–c8, costal plates 1–8; lf, left fibula; lt, left tibia; mdI–mdV, manual digits I–V; mtV, metatarsal V; m1–m12, marginal scales 1–12; nu, nuchal; pdI–pdV, pedal digits I–V; ps1–ps4, pleural scales 1–4; py, pygal; p1–p11, peripheral plates 1–11; rt, right tibia; ru, right ulna; sp1–sp2, suprapygals 1–2; v1–v5, vertebral scales 1–2; 1–8, neural plates 1–8. Scale bar equals to 50 mm.

**Figure 3:**
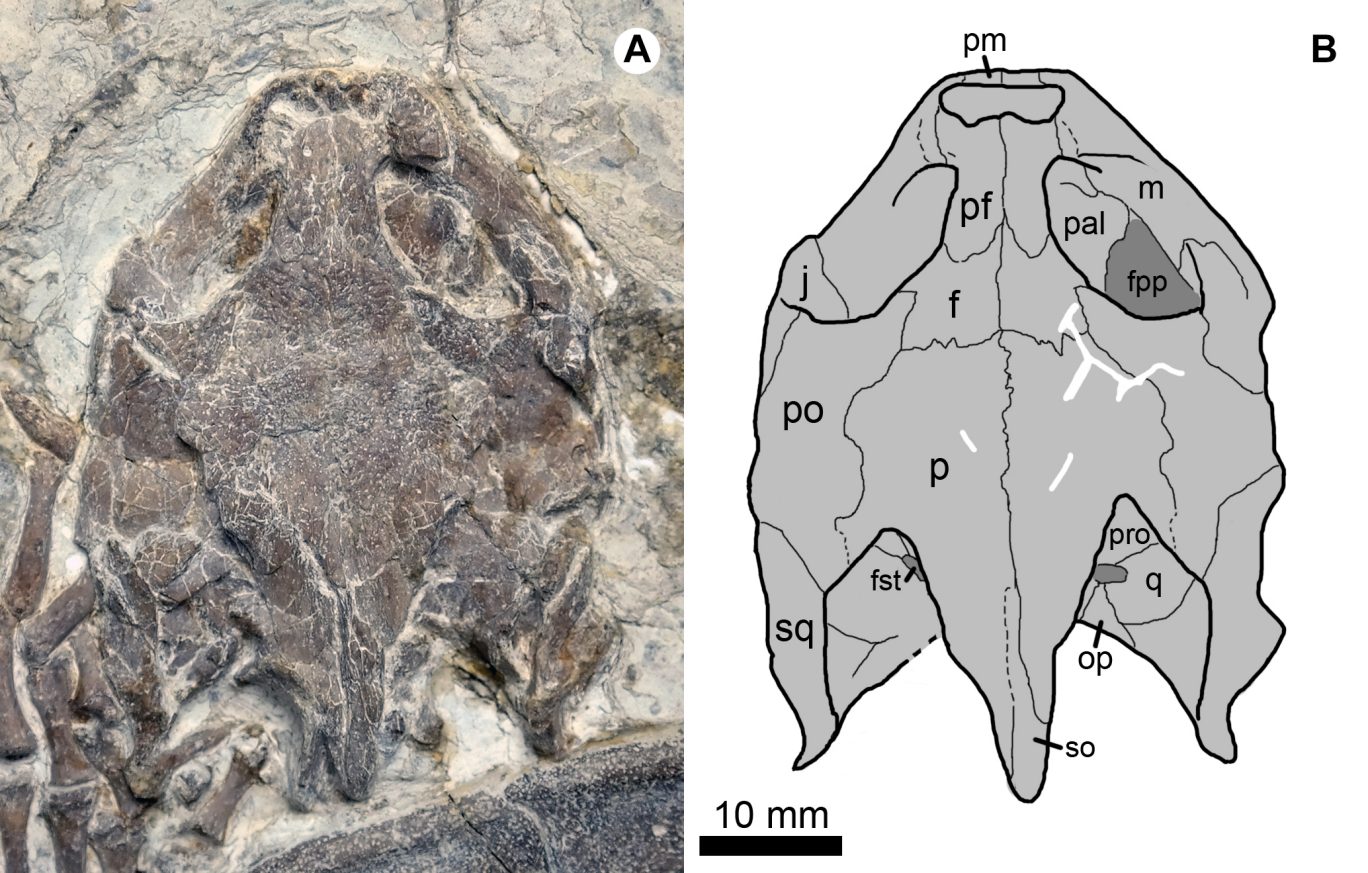
Cranial structure of *Jeholochelys lingyuanensis* gen. et sp. nov. (Holotype, PMOL-AR00211; A and B, in dorsal view). Study sites: f, frontal; fpp, foramen palatinum posterius; fst, foramen stapedio-temporale; j, jugal; m, maxilla; op, opisthotic; p, parietal; pal, palatine; pf, prefrontal; pm, premaxilla; po, postorbital; pro, prootic; q, quadrate; so, supraoccipital; sq, squamosal.

**Figure 4:**
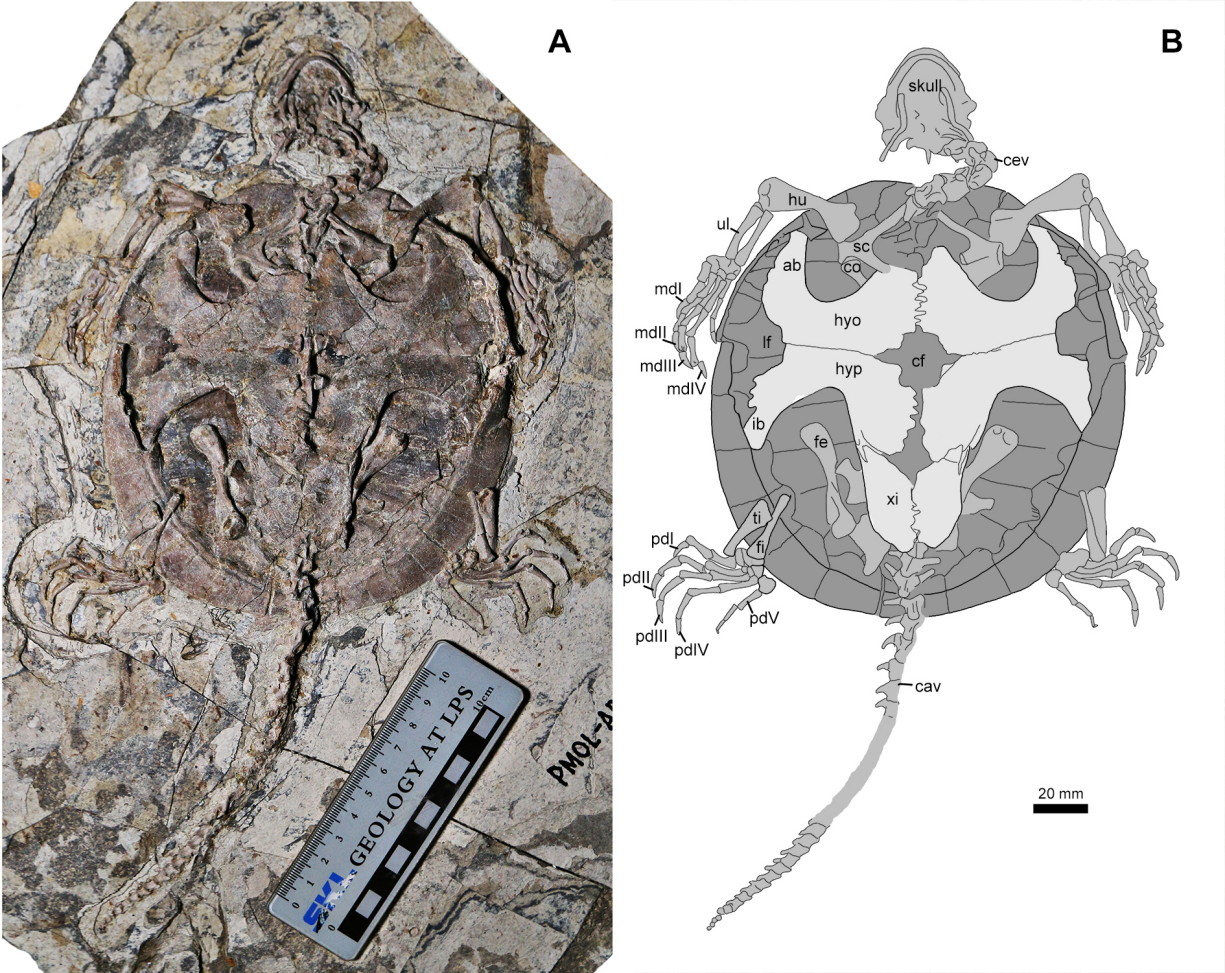
*Jeholochelys lingyuanensis* gen. et sp. nov. (PMOL-AR00213; A and B, in ventral view) from the Early Cretaceous Jiufotang Formation of Sihedang, Lingyuan, western Liaoning, China. Study sites: ab, axillary buttress; cav, caudal vertebrae; cev, cervical vertebrae; cf, central fenestra; co, coracoid; fe, femur; fi, fibula; hu, humerus; hyo, hyoplastron; hyp, hypoplaston; ib, inguinal buttress; lf, lateral fenestra; mdI–IV, manual digits I–IV; sc, scapula; ti, tibia; ul, ulna; xi, xiphiplastron.

**Figure 5:**
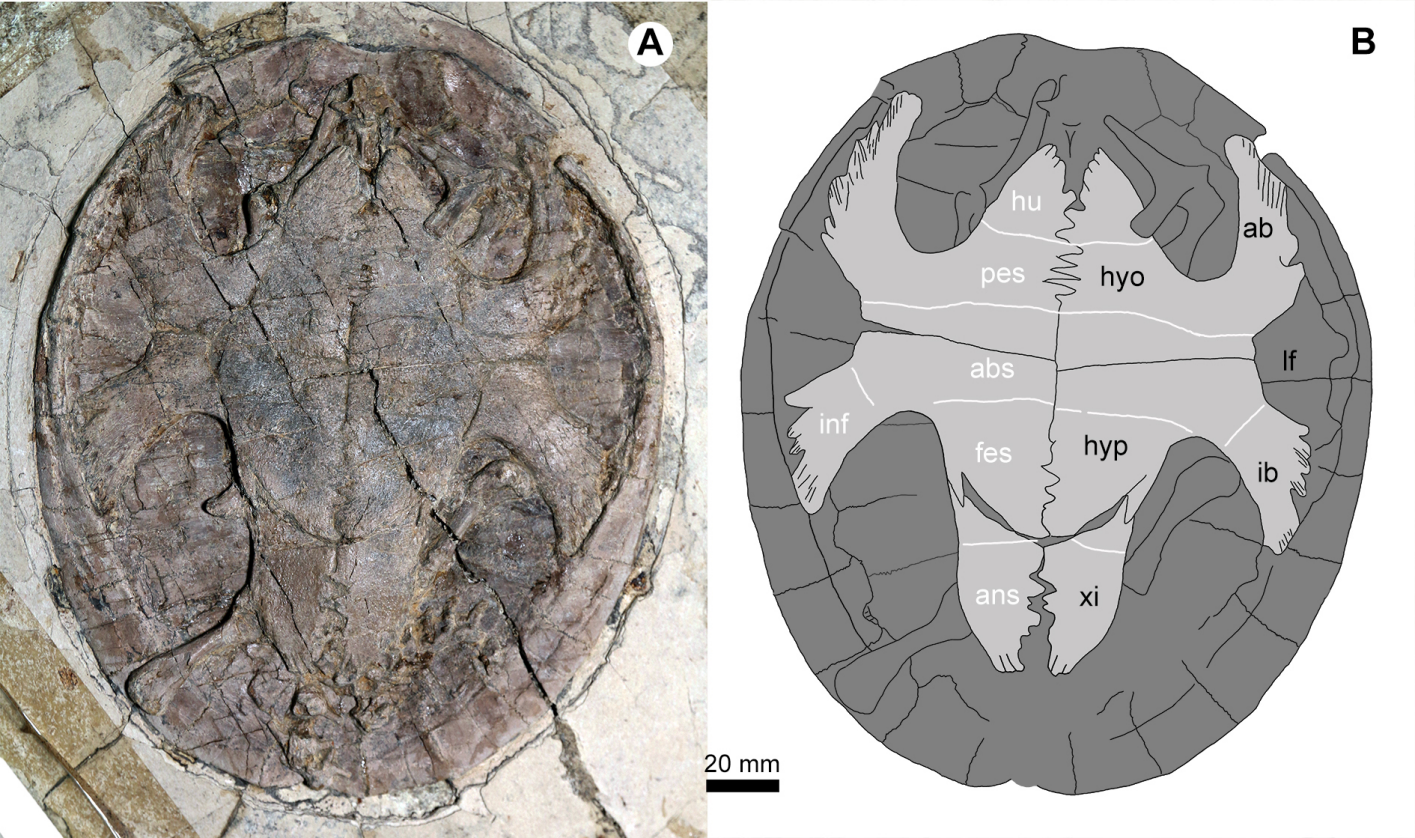
*Jeholochelys lingyuanensis* gen. et sp. nov. (PMOL-AR00218; A and B, in ventral view) from the Early Cretaceous Jiufotang Formation of Sihedang, Lingyuan, western Liaoning, China. Study sites: ab, axillary buttress; abs, abdominal scale; ans, anal scale; fes, femoral scale; hyo, hyoplastron; hu, humeral scale; hyp, hypoplaston; pes, pectoral scale; ib, inguinal buttress; inf, inframarginal scale; lf, lateral fenestra; xi, xiphiplastron.

**Figure 6:**
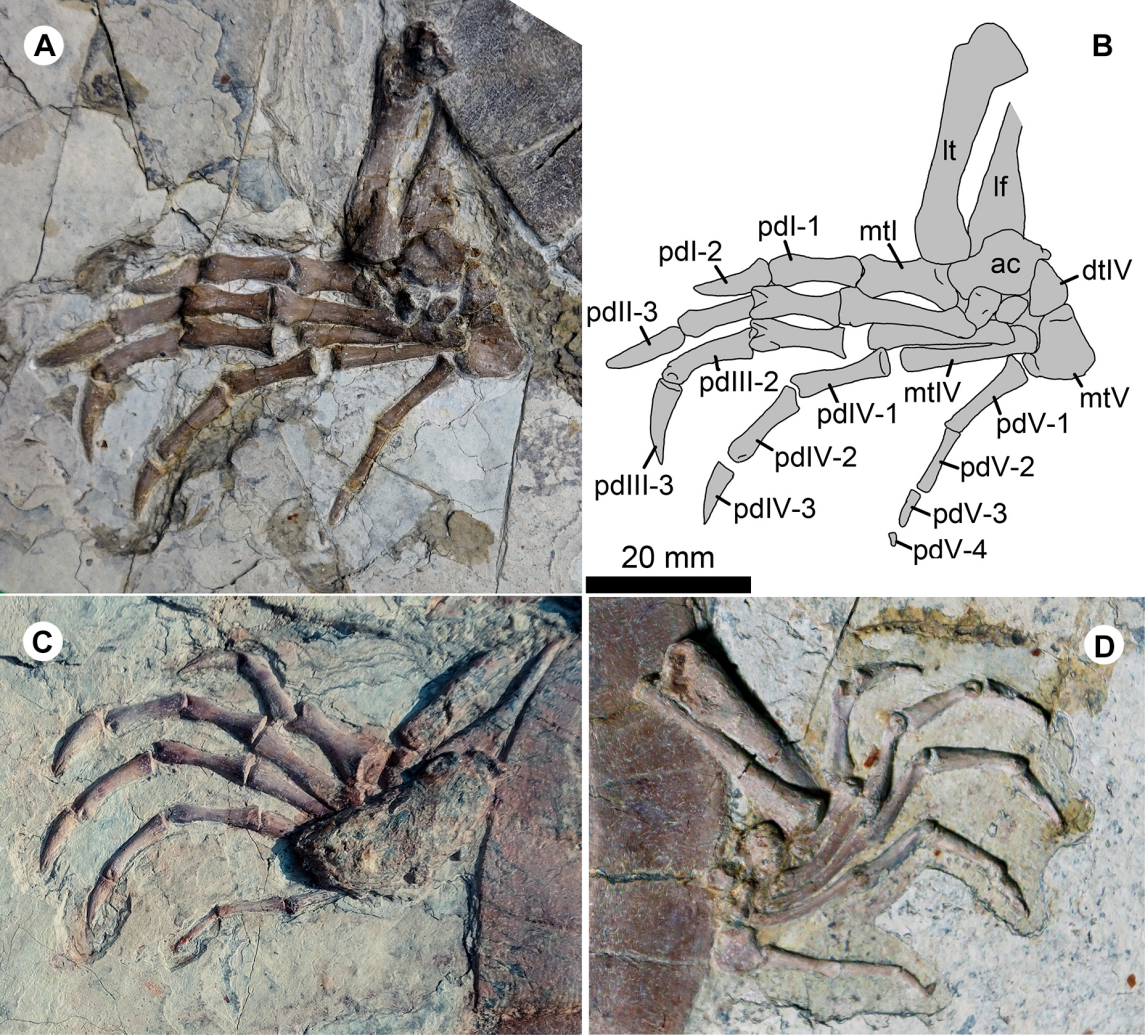
Pedes of *Jeholochelys lingyuanensis* gen. et sp. nov. The left pes of PMOL-AR00211 (Holotype; A and B) and PMOL-AR00222 (C) in dorsal view, and PMOL-AR00213 (D) in ventral view. Study sites: ac, astragalocalcaneum; dtIV, distal tarsal IV; lf, left fibula; lt, left tibia; mtI–mtV, metatarsals I–V; pdI-1–pdI-2, phalanges 1–2 of pedal digit I; PdII-1–PdII-3, phalanges 1–3 of pedal digit II; pdIII-1–pdIII-3, phalanges 1–3 of pedal digit III; pdIV-1–pdIV-3, phalanges 1–3 of pedal digit IV; pdV-1–pdV-3, phalanges 1–4 of pedal digit V.

The fossil specimens of the new taxon are recovered from the Early Cretaceous Jiufotang Formation of the Jehol Biota at a site (40°54′46″N; 119°29′45″E) near Liuligou, Sihedang Township, Lingyuan City, western Liaoning Province, China (Fig. 1). The specimens were prepared and are kept in the Paleontological Museum of Liaoning (PMOL). Seven specimens (Figs. 2–6 and Figs. S1–S4) are described in this study, five of which (PMOL-AR00190, AR00211, AR00213, AR00214, and AR00222) are nearly complete, and two others (PMOL-AR00217 and AR00218) represented by shells. Four skeletons (PMOL-AR00190, AR00213, AR00214 and AR00222) are juvenile due to open costal-peripheral fenestrae and central plastral fenestrae while other specimens have fully ossified shell.

Hundreds of turtle specimens were found at this locality during the last decade. The fossil-bearing layer is within the Sihedang beds of the third member of the Jiufotang Formation, which is a lacustrine unit mainly consisting of dark-gray mudstones intersected with green-gray shales (Zhang, 2016). Associated vertebrates include avians, pterosaurs, and non-avian dinosaurs (Wang et al., 2014; Zhou, O’Connor & Wang, 2014; Xu & Qin, 2017).

### Phylogenetic analysis

A new phylogenetic analysis is performed with TNT v1.5 beta (Goloboff & Catalano, 2016) based on the latest dataset of global turtle phylogeny (Joyce et al., 2016), which was updated from previous analyses (Joyce, 2007; Zhou, Rabi & Joyce, 2014; Zhou & Rabi, 2015). We used the traditional search option and tree-bisection-reconnection swapping algorithm with 1,000 random addition sequence replicates and 100 trees saved per replicate. All multistate characters were treated as ordered. Two runs were conducted in order to capture as many most parsimonious trees (MPTs). As in the previous analyses, the relationships of extant taxa are constrained by the molecular backbone topology of Crawford et al. (2015). Joyce et al. (2016) accidentally figured a slightly incorrect backbone topology in their supplementary file but we here follow Crawford et al. (2015). In addition, *Odontochelys semitestacea* is indicated as being part of the backbone constraint in Joyce et al. (2016) but in fact this was an error and this taxon was not constrained (M. Rabi, 2018, personal communication) and therefore we do not constrain it here either. Due to the limited memory of the program, 1,000,000 most parsimonious trees have been saved with a tree length of 969 steps (CI = 0.278; RI = 0.723). More detailed information of the phylogenetic analysis is included in the Supplementary Data.

### Comparative fossil taxa

Data on relevant basal eucryptodires come from published literature, photographs, and personal observations and include *Changmachelys bohlini* Brinkman et al., 2013; *Dracochelys bicuspis* Gaffney & Ye, 1992 (Brinkman, 2001); *Kirgizemys* (=*Hangaiemys*) *hoburensis* Sukhanov, 2000; *Kirgizemys leptis* Sukhanov & Narmandakh, 2006, *K. exaratus* Nessov & Khosatzky, 1973; *K. dmitrievi* Nessov & Khosatzky, 1981 (Danilov et al., 2006); *Judithemys sukhanovi* Parham & Hutchison, 2003; *Liaochelys jianchangensis* Zhou, 2010a (PMOL-AR00140 holotype, PMOL-AR00160, and two undescribed new specimens SDUST-V1004 and SDUST-V1005, Figs. S5 and S6); *Macrobaena mongolica* Tatarinov, 1959 (PIN 533-4 holotype); *Manchurochelys manchoukuoensis* Endo & Shikama, 1942 (PMOL-AR00007, PMOL-AR00008, PMOL-AR00180, and a undescribed new specimen PKUP V1070; Zhou, 2010b; Zhou, Rabi & Joyce, 2014; Shao et al., 2017); *Ordosemys leios* Brinkman & Peng, 1993a; *O. liaoxiensis* Ji, 1995 (a undescribed new specimen SDUST-V1020, Fig. S7; Tong, Ji & Ji, 2004); *Sinemys lens* Wiman, 1930 (Brinkman & Peng, 1993b); *S. gamera* Brinkman & Peng, 1993b; *S. brevispinus* Tong & Brinkman, 2013; *Wuguia hutubeiensis* Matzke et al., 2004; *Xiaochelys ningchengensis* Zhou & Rabi, 2015 (PMOL-AR00210 holotype).

### Nomenclatural acts

The electronic version of this article in portable document format will represent a published work according to the International Commission on Zoological Nomenclature (ICZN), and hence the new names contained in the electronic version are effectively published under that Code from the electronic edition alone. This published work and the nomenclatural acts it contains have been registered in ZooBank, the online registration system for the ICZN. The ZooBank LSIDs (Life Science Identifiers) can be resolved and the associated information viewed through any standard web browser by appending the LSID to the prefix http://zoobank.org/. The LSID for this publication is: urn:lsid:zoobank.org:pub:74F005E5-4268-4374-9983-ADA9752B8400. The online version of this work is archived and available from the following digital repositories: PeerJ, PubMed Central and CLOCKSS.

## Systematic Paleontology

Testudinata Klein, 1760
Testudines Batsch, 1788
Pan-Cryptodira Joyce, Parham & Gauthier, 2004
Sinemydidae sensu Rabi et al., 2014
*Jeholochelys lingyuanensis* gen. et sp. nov.
LSID: zoobank.org:act: 348B9DD1-4479-4EE5-88E8-9B85B36F9622
LSID: zoobank.org:act: D43DDDF6-ACAD-4450-BBAA-02E18F303593
(Figs. 2–6 and Figs. S1–S4)

### Etymology

“Jehol,” refers to “the Jehol Biota”; “chelys” is “turtle” in Greek; the specific epithet refers to the type locality.

### Holotype

Paleontological Museum of Liaoning-AR00211, an articulated and complete skeleton (Figs. 2, 3, 6A and 6B).

### Paratypes

Paleontological Museum of Liaoning-AR00190 (Fig. S1), a nearly complete skeleton exposed in dorsal view, but missing the tail and the right hindlimb; PMOL-AR00213 (Figs. 4 and 6D), a complete skeleton exposed in ventral view; PMOL-AR00214 (Fig. S2), a nearly complete skeleton exposed in dorsal view, but the posterior half of the carapace is slightly deformed; PMOL-AR00217 (Fig. S3), a complete carapace exposed in dorsal view, with disarticulated vertebrae and long bones; PMOL-AR00218 (Fig. 5), a complete shell exposed in ventral view, with disarticulated cervical and caudal vertebrae, pectoral girdles, and femora; PMOL-AR00222, a complete skeleton exposed in dorsal view (Fig. 6C and Fig. S4).

### Type locality and horizon

The fossil site (40°54′46″N; 119°29′45″E; Fig. 1) near Liuligou, Sihedang Township, Lingyuan City, western Liaoning Province; Early Cretaceous, Sihedang beds of the third member of the Jiufotang Formation (Aptian; Chang et al., 2009).

### Diagnosis

This new taxon has a low-domed shell, as in other Cretaceous sinemydids, and it is diagnosted with an unusual combination of features: nasal absent; midline contact of prefrontals; interorbital roof narrow; parietal expanded bilaterally along the supraoccipital crest; parietal separated from squamosal; supraoccipital crest slightly longer than squamosal horn; upper temporal emargination moderately developed; cranial scales present; oval carapace distinctly longer than wide; nuchal emargination shallow; preneural absent; eight neurals; two subequal suprapygals; pygal present; third costals with parallel anterior and posterior sides; cervical scale present; vertebral scales wider than long; first vertebral wider than nuchal and contact second marginal; central and posterior plastral fenestrae absent; lateral plastral fenestrae large; four phalanges of pedal digit V.

*J. lingyuanensis* differs from *Sinemys* spp. in having pygal, cervical scales, the absence of the lateral process or spine of peripheral 7, and vertebrals 2–4 wider than long. *J. lingyuanensis* differs from *M. manchoukuoensis* in having a shorter supraoccipital crest, vertebral scales 2–4 wider than long, two subequal suprapygals, and large lateral plastral fenestrae. *J. lingyuanensis* differs from *D. bicuspis* in having eight neurals, the costo-peripheral fenestrae closed, the anterolateral peripherals guttered, the presence of cervical scales, and the medial plastral fenestrae closed. *J. lingyuanensis* differs from *Kirgizemys* spp. in the absence of nasal, eight neurals, and vertebral scales 2–4 wider than long. *J. lingyuanensis* differs from *Ordosemys* spp. in having a midline contact of the prefrontals, the preneural absent, the oval carapace longer than wide, and vertebrals 2–4 narrower than vertebral 1. *J. lingyuanensis* differs from *L. jianchangensis* in having a longer supraoccipital crest, the third costals with parallel anterior and posterior sides, and vertebrals 2–4 narrower than vertebral 1. *Jeholochelys lingyuanensis* differs from *X. ningchengensis* in having an oval carapace longer than wide, and large lateral plastral fenestrae. *J. lingyuanensis* differs from *C. bohlini* in having an oval carapace longer than wide, a closed costal-peripheral fenestrae, and wider vertebral scales 2–4.

## Description

### Skull

The roof of the skull of *J. lingyuanensis* is sculptured by tiny ridges and pits (Figs. 2 and 3). Several shallow and broad sulci can be identified, possibly implying the presence of cranial scales, as in *Kirgizemys* spp. The interorbital roof is proportionally narrower than that of *K. dmitrievi* and *M. mongolica*. The preorbital portion of the skull is subtriangular, making up about 35.4% of the cranial length (from the anterior tip of the premaxilla to the posterior end of the squamosal). Posterior to the orbit, the skull is sub-rectangular, whereas it is strongly sculptured by the supraoccipital crest and squamosal crests further posteriorly. The supraoccipital crest is slightly beyond the posterior end of the squamosal, relatively longer than that of *O. liaoxiensis*, and *L. jianchangensis* (Tong, Ji & Ji, 2004; Zhou, 2010a). The temporal emargination is moderately developed, resulting in the processus trochlearis oticum unexposed in dorsal view, as in *Ordosemys* spp. and *Kirgizemys* spp.

The nasals are not present in known specimens (PMOL-AR00211 holotype, PMOL-AR00190, and PMOL-AR00222). The prefrontals are well preserved and form the interorbital roof with the frontals. Anteriorly, the prefrontals enclose the external naris with the maxillae and the premaxillae. Laterally, the prefrontal bears a stout process to contact the maxilla, and this process along with the maxilla separate the external naris from the orbit. Medially, the prefrontals meet along most of their length, and are partially separated posteriorly by the frontals, as a contrast to *Sinemys* spp. and *Ordosemys* spp., in which the prefrontals are entirely separated (Brinkman & Peng, 1993a, 1993b; Tong, Ji & Ji, 2004). Posteriorly, the frontals are positioned among the prefrontals anteriorly, the postorbitals laterally and the parietals posteriorly. The frontal has a limited contribution to the orbital margin.

The parietal is a large element of the cranial roof. The parietals meet firmly along the midline, and separated posteriorly by the supraoccipital crest. The posterior portion of the parietal is shelf-like and expands laterally, partially overhanging the foramen stapedio-temporale, as in *Kirgizemys* spp. (Sukhanov, 2000; Danilov et al., 2006). Posteriorly, the parietal almost reaches the posterior end of the supraoccipital crest. Along the upper temporal emargination, the parietal bears a lateral process and possibly fails to contact the squamosal.

The small premaxillae are positioned at the anterior tip of the skull. They form the ventral rim of the naris with the maxillae. The maxilla forms the ventral margin of the orbit along the buccal side, and bears a dorsal process to contact the prefrontal. The maxilla encloses the foramen palatinum posterius with the medially-positioned palatine, as exposed through the orbit.

The jugal is partially exposed at the posteroventral corner of the orbit. The postorbital is posterodorsal to the orbit, and possibly forms the rim of the temporal emargination with the parietal and squamosal. The squamosal bears a low crest that forms the lateral margin of the upper temporal emargination. The squamosal is pinched posteriorly and directed posterolaterally. Its posterior end fails to reach the distal end of the supraoccipital crest.

The prootic, the opisthotic, and the quadrate can be identified through the upper temporal emargination. They probably enclose the foramen stapedio-temporale, which is partially obscured by the parietal.

### Axial skeleton

The cervical series is well exposed in articulation in PMOL-AR00213 (Fig. 4). Cervical vertebra 3 appears to have a posterior cotyle, possibly implying an opisthocoelous condition. Cervical vertebrae 4 is biconvex. The remaining cervicals are possibly procoelous. As in other sinemydids, the transverse processes of the cervical vertebra are anteriorly positioned. The ventral keels of the cervical vertebra are well developed. The cervical ribs are not observed in known specimens, and possibly absent in *Jeholochelys lingyuanensis*. In contrast, the cervical ribs are present in *L. jianchangensis* (Zhou, 2010a), which coexisted with *Jeholochelys* *lingyuanensis*.

As in other coexisting turtles, the tail of *J. lingyuanensis* is elongate, comparable with the carapace length. In PMOL-AR00213 (Fig. 4), a complete tail is preserved with 30 caudal vertebrae. The anterior five caudals are procoelous, the sixth is amphicoelous, and the remaining caudal vertebrae are opisthocoelous. The position of the amphicoelous caudal vary in the known sinemydids, and for example it is the sixth caudal in *J. sukhanovi*, the third caudal in *Ordosemys* spp., and is the fourth in *S. brevispinus* (Brinkman & Peng, 1993a; Parham & Hutchison, 2003; Tong, Ji & Ji, 2004; Tong & Brinkman, 2013). The transverse processes are spine-like. The size of transverse processes reduces posteriorly along the caudal series, and they disappear on the 20th caudal. The first three caudal centra bear ventral keels like in the sacral vertebrae. On the lateral surface, the caudal is sculptured by one or two foramina. The chevron is well developed and attached firmly at the posteroventral corner of the centrum along the entire length of the tail.

### Appendicular skeleton

The pectoral girdle is poorly exposed in ventral view (Figs. 4 and 5). The scapula is notable with the bifurcate scapular and acromial processes. The scapula bears a distinct neck to participate in the glenoid fossa. The glenoid fossa comprises the scapula and the coracoid. The coracoid bears a strong constriction posterior to the glenoid fossa, and then becomes flat distally.

The humerus is a massive element with two expanded ends. Proximally, the lateral process is developed. Distally, the ectepicondylar foramen is exposed in dorsal view. The ulna and the radius are much slender and shorter than the humerus. The ulna is more robust than the radius, and both bones are subequal in length.

The manus is moderately elongate, about 172–179% of the length of the ulna, comparable with that of the soft-shelled turtles (Joyce & Gauthier, 2004). The length increases gradually from metacarpal I to metacarpal IV, and metacarpal V has a length between metacarpals I and II. The width of the shaft also reduces gradually from metacarpal I to metacarpal V. Metacarpal I is the shortest and most robust metacarpal. The five digits are clawed and with a phalangeal formula of 2-3-3-3-3, as the plesiomorphic condition of crown turtles (Joyce, 2007). Digits III and IV are elongate and subequal in length. The claws of digits II–III are larger than other ungual phalanges; while the claw of digit V is reduced and uncurved.

The pelvic girdles are poorly exposed in the known specimens, while the hindlimbs are well exposed in dorsal or ventral views (Figs. 2 and 4). The femur is robust, and is slightly longer than the humerus. The slender tibia and fibula are comparable in length. In the known specimens, the astragalus and calcaneum are fused as the astragalocalcaneum, which becomes the largest tarsal element.

The pes is well preserved (Figs. 2, 4 and 6). The curved metatarsal V (= the ansulate bone in Joyce, Werneburg & Lyson, 2013) is notable with a large size. In contrast, metatarsals I–IV are elongate and rod-like. Metatarsal I is the shortest and the most robust among the metatarsals, while metatarsal III is the longest. The phalangeal formula is 2-3-3-3-4, different from the primitive condition of 2-3-3-3-3 in the crown turtles. The additional phalanx of digit V is also known in few extant turtles and fossil turtles as hyperphalangy (Zug, 1971; Ludwig, Auer & Fritz, 2007; Delfino, Fritz & Sánchez-Villagra, 2010). The additional phalanx V-4 is small and unclawed. The pedal claws in digits I–IV appear to be more robust than the manual claws. The size of the pedal claws is reduced gradually along the series.

### Carapace

The oval carapace is distinctly longer than it is wide, different from *Ordosemys* spp., *C. bohlini*, and *X. ningchengensis*, in which the carapace is subequal in width and length (Brinkman & Peng, 1993a; Tong, Ji & Ji, 2004; Brinkman et al., 2013; Zhou & Rabi, 2015). The posterior half of the carapace is constricted and tightly curved, as in *O. liaoxiensis*, *M. manchoukuoensis*, *D. bicuspis*, and *L. jianchangensis*. The carapacial surface is generally smooth. Plications are posteriorly-directed and weakly developed along the vertebral sulci in the known specimens. However, in the holotype, some plications are well developed along the sulci of marginals 6–11 (Fig. 2), but the plications are absent from these positions in other specimens (PMOL-AR00214 and PMOL-AR00217; Figs. S3 and S4).

The nuchal plate is large and forms a shallow emargination, as in *M. manchoukuoensis*, *Kirgizemys* spp., *L. jianchangensis*, and *X. ningchengensis*, and different from *C. bohlini* and *Ordosemys* spp., in which the emargination is more developed (Tong, Ji & Ji, 2004; Brinkman et al., 2013). The nuchal contacts the first peripherals laterally, the first costals posterolaterally, and the first neural posteriorly. In contrast, a limited contact of the nuchal and second peripherals is present in *O. leios*, *O. liaoxiensis* and *D. bicuspis* (Brinkman & Peng, 1993a; Brinkman, 2001; Tong, Ji & Ji, 2004).

The neural series is complete and composed of eight elements. The first three neurals are subrectangular and large. Neurals 4–7 appear to be hexagonal with very short anterolateral sides. They decrease in size along the series, and neural 7 is the smallest. Neural 7 tapers posteriorly to form a narrow contact with neural 8. Neural 8 is subtrapezoidal with a longer posterior side.

Two trapezoidal suprapygals are present and comparable in size, different from *M. manchoukuoensis* and *L. jianchangensis* in which the first suprapygal is small (Zhou, 2010a, 2010b). The first suprapygal is separated from the peripherals by a broad contact with the second suprapygal and the eighth costals, as in *M. manchoukuoensis*, *L. jianchangensis*, *K. leptis*, *O. liaoxiensis*, and *X. ningchengensis*. The suture between the two suprapygals is nearly straight. Distally, the suprapygal-pygal suture is distinctly beyond the sulci of vertebral and marginal scales.

The pygal is a small element between the eleventh peripherals, forming a shallow emargination at the distal end of the shell.

A total of eight pairs of costal plates are present. The costo-peripheral fenestrae are fully closed in the large individuals, while a partially-closed condition is present in the smaller ones (PMOL-AR00190, PMOL-AR00124, and PMOL-AR00222), as a juvenile feature that is also observed in other sinemydids (Tong, Ji & Ji, 2004; Shao et al., 2017). The first costal plate has a relatively broad contact with the first peripheral. Posteriorly, the costal plates widen gradually, reaching the maximum width at the fourth costal plate. Costals 2 and 3 are subparallel in anterior and posterior margins, unlike the distally-convergent costal 2 and the distally-expanded costal 3 of *L. jianchangensis* (Zhou, 2010a). Costal 4 is slightly expanded distally. The succeeding costals are gradually reduced in size and directed obliquely. The distal ends of the posterior costals have a tiny extrusion within the peripherals in dorsal view, like in *L. jianchangensis*, *S. brevispinus*, *O. liaoxiensis*, *K. exaratus*, *M. manchoukuoensis*, *D. bicupis*, and *X. ningchengensis* (Sukhanov, 2000; Brinkman, 2001; Tong, Ji & Ji, 2004; Zhou, 2010a, 2010b; Tong & Brinkman, 2013; Zhou & Rabi, 2015).

A total of 11 pairs of peripheral plates form the carapace with the nuchal and the pygal. Along the peripheral ring, a shallow gutter extends to peripheral 6 on each side. Peripheral 1 is small and subtriangular. Peripherals 2 and 3 are enlarged and have a rectangular outline. Their medial sides are beyond the pleural-marginal sulci. Peripherals 4, 5, and 6 are slender and gradually increase in size. Their sutures with the costals are confluent with the pleural-marginal sulci. In contrast, peripherals 7–11 are distinctly enlarged and expanded medially beyond the pleural-marginal sulci.

### Carapacial scales

The carapacial scalation is distinct (Fig. 2). The cervical scale is slender and limited within the nuchal emargination. The vertebrals are wider than long, different from a longer condition in vertebrals 2–4 of *Sinemys* spp., *M. manchoukuoensis*, *Kirgizemys* spp., *W. hutubeiensis*, and *C. bohlini* (Brinkman & Peng, 1993b; Matzke et al., 2004; Danilov et al., 2006; Zhou, 2010b; Brinkman et al., 2013). Vertebrals 2–4 are comparable with the pleurals in width. In contrast, vertebrals 2–4 are wider than the pleurals in *Ordosemys* spp., *L. jianchangensis*, and *X. ningchengensis* (Brinkman & Peng, 1993a; Tong, Ji & Ji, 2004; Zhou & Rabi, 2015).

Vertebral 1 is slightly wider than the nuchal. Vertebrals 2–4 are hexagonal. Vertebrals 2 and 3 have comparable sizes to each other, and vertebral 4 is slightly smaller. The vertebral 1–2 sulcus has a small anterior midline projection across the mid-portion of neural 1. A similar condition is also present in other inter-vertebral sulci, associated with shallow sulcus plications. The vertebral 3–4 sulcus crosses the posterior portion of neural 5. The posterior side of vertebral 4 is reduced, and its posterolateral sides are slightly longer than the anterolateral sides. Vertebral 5 is subpentagonal, and it is larger than the suprapygals and extends onto peripherals 10 and 11 laterally.

Pleurals are paired and bilaterally positioned along the vertebrals. Pleurals 1–3 are wider than long. Pleural 4 is reduced in size and has a similar width and length.

The marginals increase in size posteriorly. They are restricted to the peripherals, except for the first marginals that extend onto the nuchal. Marginal 2 has a broad contact with vertebral 1, unlike the point-like contact in *O. leios*, *O. liaoxiensis*, and *J. sukhanovi* (Brinkman & Peng, 1993a; Parham & Hutchison, 2003; Tong, Ji & Ji, 2004). The pleuro-marginal sulci of marginals 4–6 match up with the costo-peripheral suture; while the pleuro-marginal sulci of marginals 7–11 are limited on the associated peripherals. Marginals 12 meet each other along the midline, and cover the pygal at the distal end of the carapace. In the holotype, marked sulcus plications are unusually present at corner point of marginals 6–11. The plication are directed posterolaterally without crossing the marginal scale. However, this feature is absent in other specimens, possibly representing an individual variation.

### Plastron

As in other sinemydids, the plastron connects to the carapace via ligaments and pegs. It is cruciform, as wide as it is long, different from the wider than long condition in *J. sukhanovi* (Parham & Hutchison, 2003). In PMOL-AR00213 (Fig. 4), the plastral fenestrae are developed, whereas in the larger individual (PMOL-AR00218; Fig. 5), central and posterior (hypo-xiphiplastra) fenestrae are closed, and two large and paired lateral fenestrae are developed. In contrast, the lateral fenestrae are extremely reduced in *K. exaratus* and *J. sukhanovi* (Sukhanov, 2000; Parham & Hutchison, 2003). The anterior lobe is triangular and stout. The posterior lobe is elongate and wide. It has a broad base and tapers distally, forming a narrow distal end. The bridge is anteroposteriorly short, less than 30% of the plastral length. It is distinctly longer than the anterior lobe, but shorter than the posterior lobe. The plastral surface is sculptured by numerous pits and ridges.

The epiplastra and entoplastron are not preserved in any known specimens (PMOL-AR00213 and PMOL-AR00218). The hyoplastra are large elements, anteriorly separated by the entoplastron, but they meet each other posteriorly along a jagged suture. Laterally, the hyoplastron bears a deep axillary notch along its anterior margin. The axillary buttress wedges into the medial fossa of peripheral 2. Posteriorly, the hyoplastron has a nearly straight suture with the following hypoplastron. The inguinal notch is well-developed, and defines a shorter bridge with the axillary notch anteriorly. The inguinal buttress is relatively stout, in contrast to the slender buttress in *L. jianchangensis* (Zhou, 2010a). The xiphiplastra are reduced and form a relatively sharp distal margin of the posterior lobe.

The plastral scalation is partially exposed in PMOL-AR00218. The major elements, including the humeral scales, the pectoral scales, the abdominal scales, the femoral scales and the anal scales are observed. The humeral-pectoral sulcus is nearly straight, and is behind the entoplastron. The pectoral scales are restricted to the hyoplastron. The pectoral-abdominal and abdominal-femoral sulci are also nearly straight. The femoral-anal sulcus is confluent with the hyo-xiphiplastral suture medially. The inframarginal scales are developed, but their number is uncertain.

## Discussion

### Phylogenetic position of *Jeholochelys lingyuanensis*

**Figure 7:**
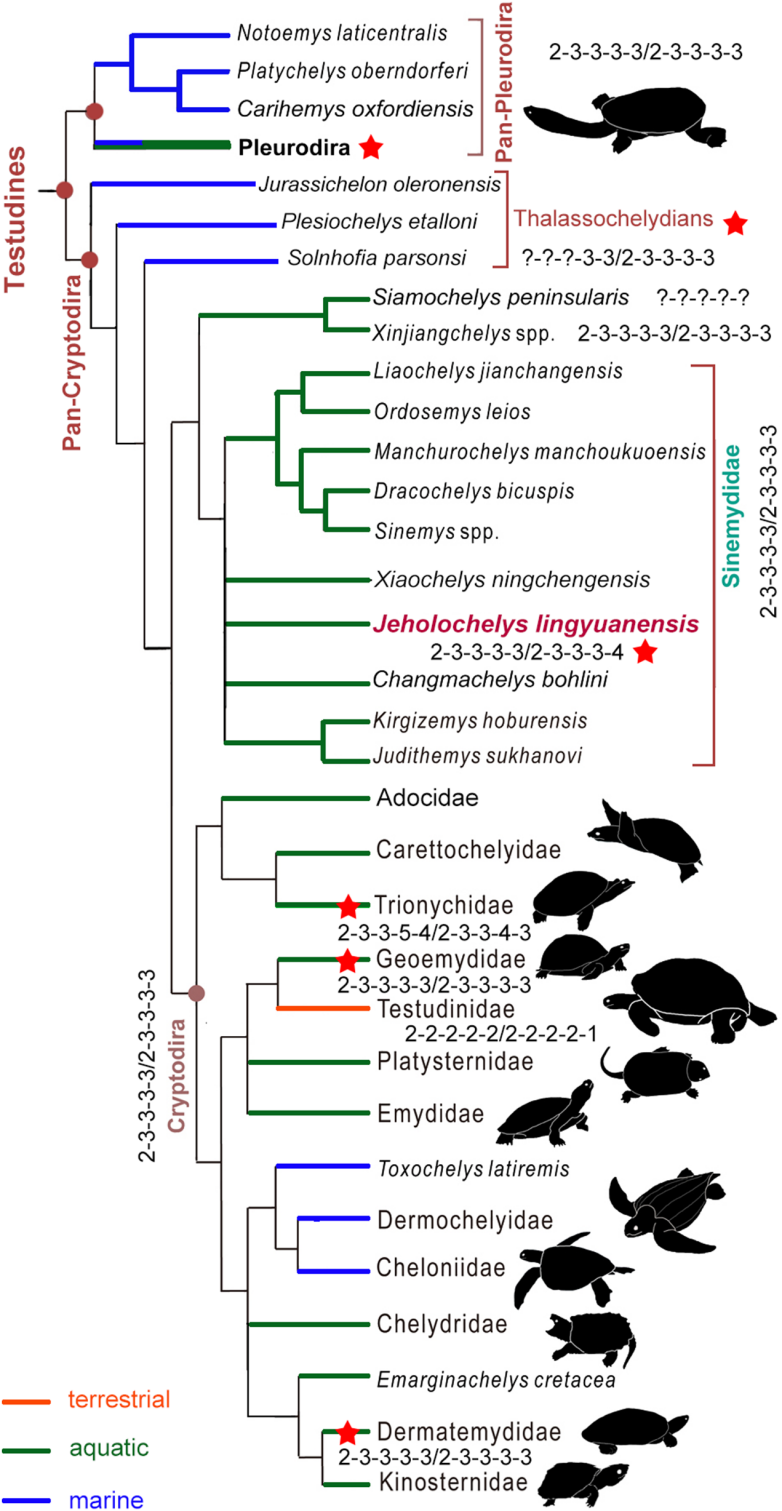
Simplified strict consensus tree of the phylogenetic analysis showing the position of *Jeholochelys lingyuanensis* gen. et sp. nov. Hyperphalangy is marked by red stars with the manual/pedal phalangeal formula depicted. Turtle silhouettes from Zhou & Rabi (2015).

*J. lingyuanensis* was recovered in the clade of Sinemydidae (sensu Rabi et al., 2014) in our phylogenetic analysis (Fig. 7). In the strict consensus tree, *Jeholochelys lingyuanensis* is positioned in a polytomy with *X. ningchengensis*, *C. bohlini*, the *K. hoburensis*–*Judithemys sukhanovi* clade, and the rest of sinemydids. The clade of Sinemydidae is weakly supported by four synapomorphies, which include paired pits on the ventral surface of basisphenoid (78-1), narrow and elongate epiplastra and entoplastron (142-1), distal end of dorsal rib visible only within the costo-peripheral fenestrae on the dorsal face of the carapace (230-1), and cruciform plastron (233-1).

*J. lingyuanensis* probably introduced character conflict relative to other members of the Sinemydidae. For example, the cranial structure of *Jeholochelys lingyuanensis* is more similar to that of *C. bohlini*, *Judithemys sukhanovi*, *Kirgizemys* spp., and *X. ningchengensis*. These features include a long supraoccipital crest and the laterally expanded parietal. In contrast, these features are less developed in most of the other sinemydids (e.g., *Sinemys* spp., *M. manchoukuoensis*, *Ordosemys* spp., *L. jianchangensis*; Brinkman & Peng, 1993a, 1993b; Tong, Ji & Ji, 2004; Zhou, 2010a, 2010b). On the other hand, vertebrals 2–4 are almost as wide as long in *Jeholochelys lingyuanensis*, like that of *D. bicuspis* and *Judithemys sukhanovi*, but different from the proportionally wide vertebrals (e.g., *Ordosemys* spp., *L. jianchangensis*, and *X. ningchengensis*; Brinkman & Peng, 1993a; Tong, Ji & Ji, 2004; Zhou, 2010a; Zhou & Rabi, 2015) and the proportionally narrow vertebrals (e.g., *Sinemys* spp., *M. manchoukuoensis*, *Kirgizemys* spp.; Brinkman & Peng, 1993b; Sukhanov, 2000; Zhou, 2010b) in other taxa. The central plastral fenestrae are closed in adult individuals, as in most of sinemydids, but different from the open condition in *Ordosemys* spp., *L. jianchangensis*, *D. bicuspis*, *C. bohlini*, and *S. brevispinus* (Brinkman, 2001; Tong, Ji & Ji, 2004; Zhou, 2010a; Brinkman et al., 2013; Tong & Brinkman, 2013). However, the large lateral plastral fenestrae are comparable to that of *O. liaoxiensis*, *L. jianchangensis*, *D. bicuspis*, and *C. bohlini*, but different from a reduced or enclosed condition in other sinemydids (Brinkman & Peng, 1993a, 1993b; Sukhanov, 2000; Zhou & Rabi, 2015).

### Hyperphalangy of the crown turtles

In the crown turtles, the variation of the phalangeal counts of the manus and the pes is associated with the adaptation to terrestrial or aquatic environments. The terrestrial turtles tend to have decreased phalangeal count and shorter phalanges compared to freshwater turtles. Apart from this variation, the phalangeal formula of 2-3-3-3-3 for both the manus and the pes is widely distributed (Fig. 7) among crown turtles including stem-cryptodires and pleurodires (e.g., thalassochelydians, xinjiangchelyids and sinemydids, and platychelyids; Joyce, 2000; Tong, Ji & Ji, 2004; Karl & Tichy, 2006; Zhou, 2010a, 2010b; Brinkman et al., 2013; Rabi et al., 2013; Anquetin, Püntener & Joyce, 2017) and is therefore considered the plesiomorphic condition of the crown (Joyce, 2007; Sánchez-Villagra, Winkler & Wurst, 2007).

Hyperphalangy is rare in turtles, and is mostly found in representatives of Trionychidae with inter- and infraspecific variations. Trionychids have a distinctly increased phalangeal count (2-3-3-6-5/2-3-3-5-4 in the maximum manual/pedal phalangeal count) (Delfino, Fritz & Sánchez-Villagra, 2010). Hyperphalangy is otherwise present in few extant turtles, all of which are aquatic, including the pleurodiran *Acanthochelys pallidipectoris* (additional phalanx in manual digit IV), *Phrynops* spp. (a pedal phalangeal formula of 2-3-3-3-5), and the cryptodiran *Dermatemys mawii* and *Pangshura smithii* (supplementary phalanx in pedal digit V) (Zug, 1971; Ludwig, Auer & Fritz, 2007; Sánchez-Villagra, Winkler & Wurst, 2007; Bona & Alcalde, 2009; Delfino, Fritz & Sánchez-Villagra, 2010).

Hyperphalangy among fossil crown turtles has been reported for the Early Cretaceous soft-shelled species *Perochelys lamadongensis* (pedal phalangeal formula of 2-3-3-4-?; Li, Joyce & Liu, 2015), and in the primitive marine pancryptodiran *Neusticemys neuquina* from the Late Jurassic (pedal phalangeal formula of 2-3-3-3-5 (2-3-3-3-4 in De la Fuente & Fernández, 2011, as they considered the hooked element as distal tarsal V)). In contrast, other thalassochelydians (sensu Anquetin, Püntener & Joyce, 2017, but is recovered as paraphyletic in this study, including plesiochelyids, eurysternids, and thalassemydids; Fig. 7) have the plesiomorphic condition (2-3-3-3-3) of crown turtles (Joyce, 2000; Anquetin & Joyce, 2014; Anquetin, Püntener & Joyce, 2017).

In the sinemydid *J. lingyuanensis*, the additional phalanx is a tiny element, observed in four specimens (PMOL-AR00211, AR00213, AR00214, and AR00222; Figs. 2, 4 and 6) in which the pedal digits are completely preserved. We therefore interpret the pedal phalangeal formula of 2-3-3-3-4 as typical for *J. lingyuanensis*, instead of a infraspecific variation like in some extant turtles (Zug, 1971; Ludwig, Auer & Fritz, 2007; Sánchez-Villagra, Winkler & Wurst, 2007; Bona & Alcalde, 2009; Delfino, Fritz & Sánchez-Villagra, 2010). In contrast, the additional phalanx in pedal digit V is absent in other coexisting sinemydid turtles, e.g., *C. bohlini* (Brinkman et al., 2013), *M. manchoukuoensis* (PKUP V1070), *L. jianchangensis* (SDUST-V1004 and SDUST-V1005; Figs. S5 and S6) and *O. liaoxiensis* (SDUST-V1020; Figs. S7).

In soft-shelled turtles, the additional phalanges are present in the unclawed digits IV and V of both the manus and pes, possibly to achieve higher mobility in water by enlarging the paddle surface (Delfino, Fritz & Sánchez-Villagra, 2010). Similarly, one additional phalanx of the unclawed digit V is present in *Jeholochelys lingyuanensis*. As mentioned above, the proportions of the forelimb in *J. lingyuanensis* is comparable to that of soft-shelled turtles, possibly implying a similar degree of aquatic adaptation like in trionychids (Joyce & Gauthier, 2004).

## Supplemental Information

10.7717/peerj.5371/supp-1 Supplemental Information 1 Supplementary Information. Supplementary Figures and Supplementary Data.

## Institutional Abbreviations

PIN: Paleontological Institute, Russian Academy of Sciences, Moscow, Russia
PMOL: Paleontological Museum of Liaoning, Shenyang Normal University, Shenyang, China
PKUP: Paleontological Collections, School of Earth and Space Sciences, Peking University, Beijing, China
SDUST: College of Earth Science and Engineering, Shandong University of Science and Technology, Qingdao, China

## Additional Information and Declarations

### New Species Registration

The following information was supplied regarding the registration of a newly described species: Publication LSID: urn:lsid:zoobank.org:pub:74F005E5-4268-4374-9983-ADA9752B8400 Genus name: Jeholochelys ur:Isid:zoobank.org:act:348B9DD1-4479-4EE5-88E8-9B85B36F9622, Species name: Jeholochelys lingyuanensis ur:Isid:zoobank.org:act:D43DDDF6-ACAD-4450-BBAA-02E18F303593.

### Data Availability

The following information was supplied regarding data availability: The specimens of *Jeholochelys lingyuanensis* described in this study are stored in Paleontological Museum of Liaoning, Shenyang Normal University, Shenyang, China. Accession numbers: PMOL-AR00190, PMOL-AR00211, PMOL-AR00213, PMOL-AR00214, PMOL-AR00217, PMOL-AR00218, PMOL-AR00222.
