# Supplementary material for: Hyperphalangy in a new sinemydid turtle from the Early Cretaceous Jehol Biota
Source: PeerJ. 2018 Jul 27;6:e5371. doi: 10.7717/peerj.5371 (PMC6065475; doi:10.7717/peerj.5371)
Supplement: Supplemental Information 1 — Supplementary Figures and Supplementary Data. [file peerj-06-5371-s001.docx]

**Supplementary Information**

**Hyperphalangy in a new sinemydid turtle from the Early Cretaceous Jehol Biota**

**Shuai Shao^1,2^, Lan Li^3^, Yang Yang^4^, Chang-Fu Zhou^5^**

^1^Research Center of Palaeontology & Stratigraphy, Jilin University, Changchun, Jilin Province, China

^2^Paleontological Institute, Shenyang Normal University, Shenyang, Liaoning, China

^3^School of Earth and Space Sciences, Peking University, Beijing, China

^4^Anhui Geological Museum, Anhui, China

^5^College of Earth Science and Engineering, Shandong University of Science and Technology, Qingdao, Shandong Province, China

Corresponding author: Chang-Fu Zhou, zhoucf528@163.com

**Including:**

**Supplementary Figures.**

**Supplementary Data.**

**Supplementary Figures.**

**Figure S1. PMOL-AR00190 of *Jeholochelys lingyuanensis* gen. et sp. nov.**

**
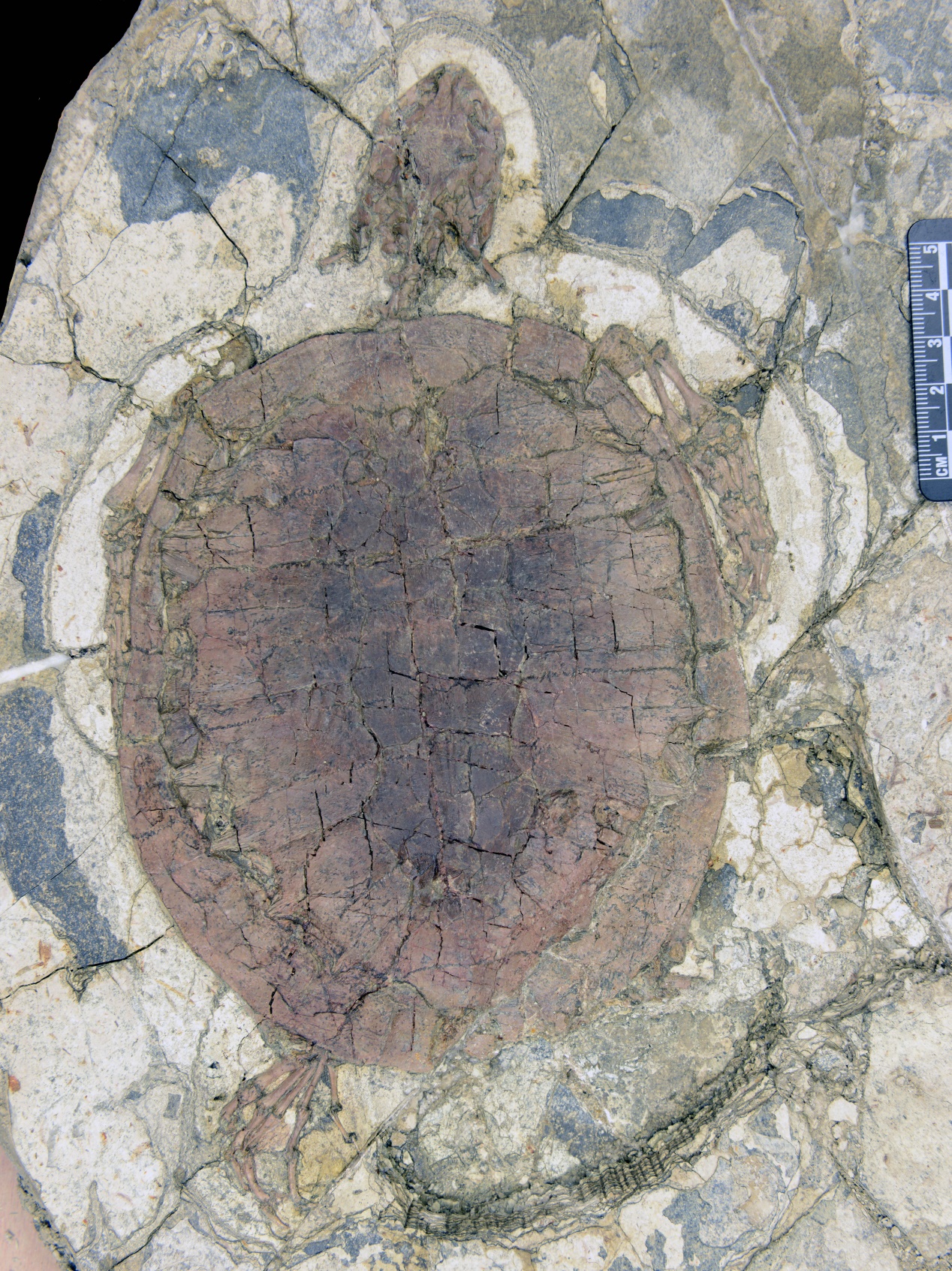
**

**Figure S2. PMOL-AR00214 of *Jeholochelys lingyuanensis* gen. et sp. nov.**

**
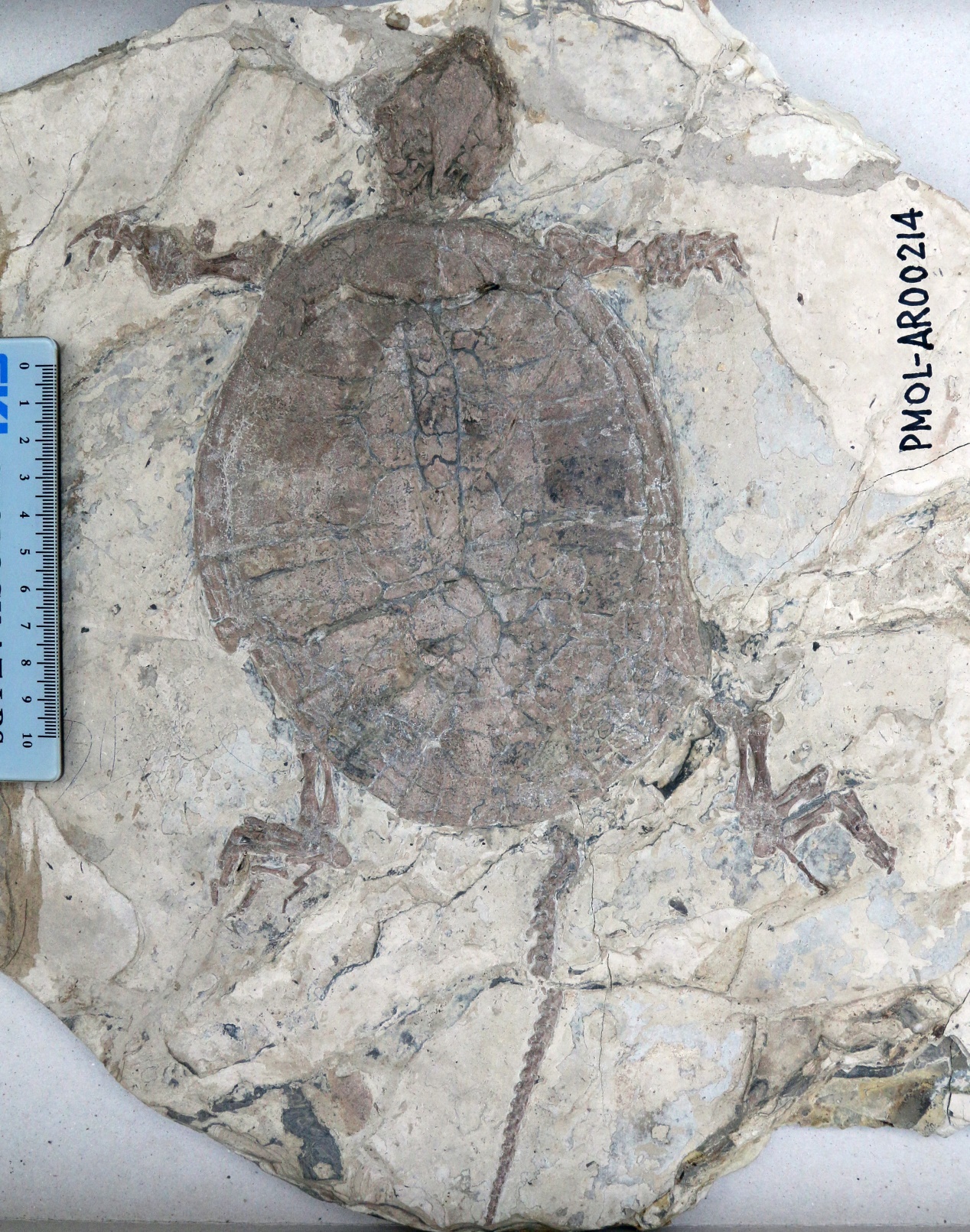
Figure S3. PMOL-AR00217 of *Jeholochelys lingyuanensis* gen. et sp. nov.**

**
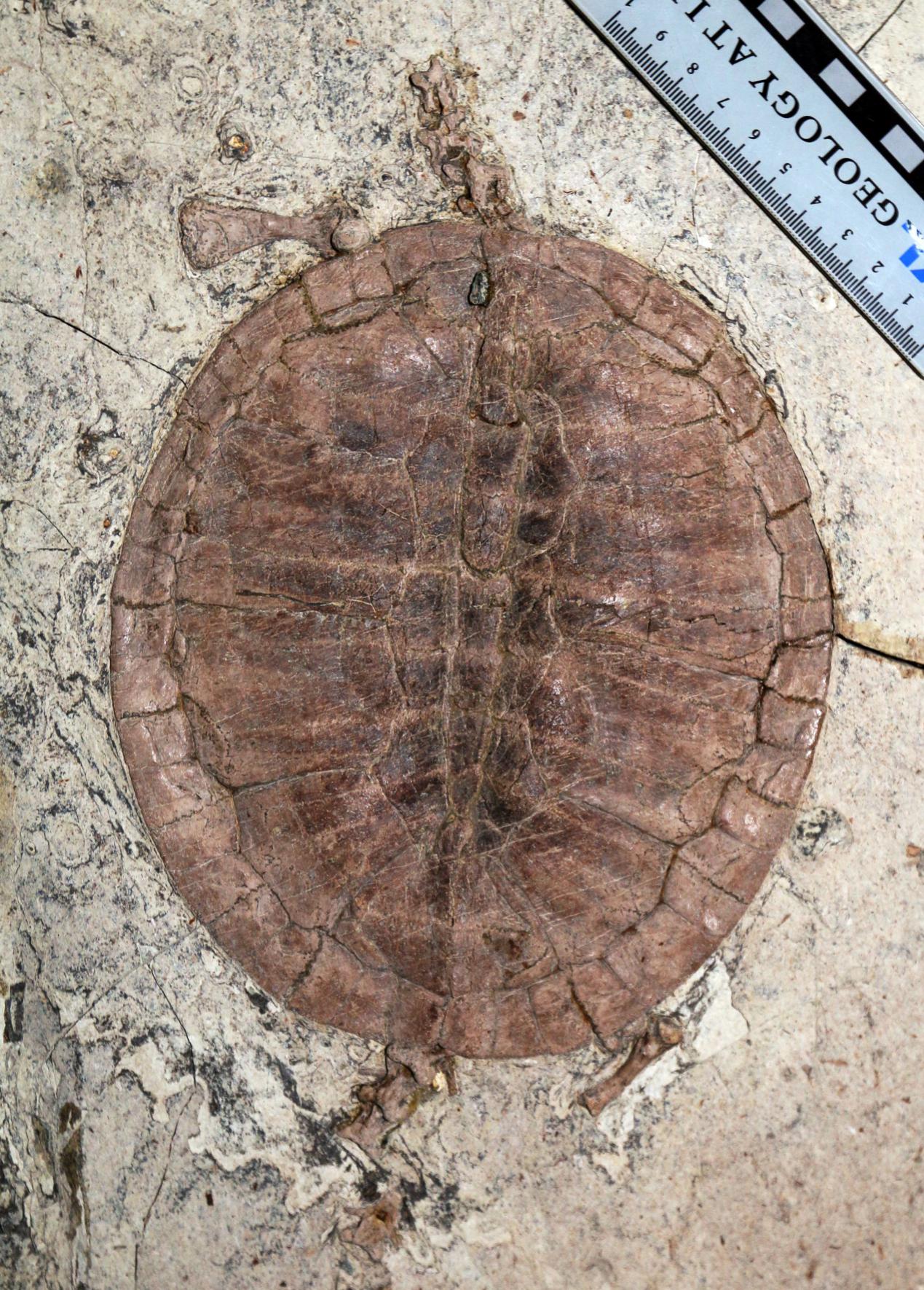
**

**Figure S4. PMOL-AR00222 of *Jeholochelys lingyuanensis* gen. et sp. nov.**

**
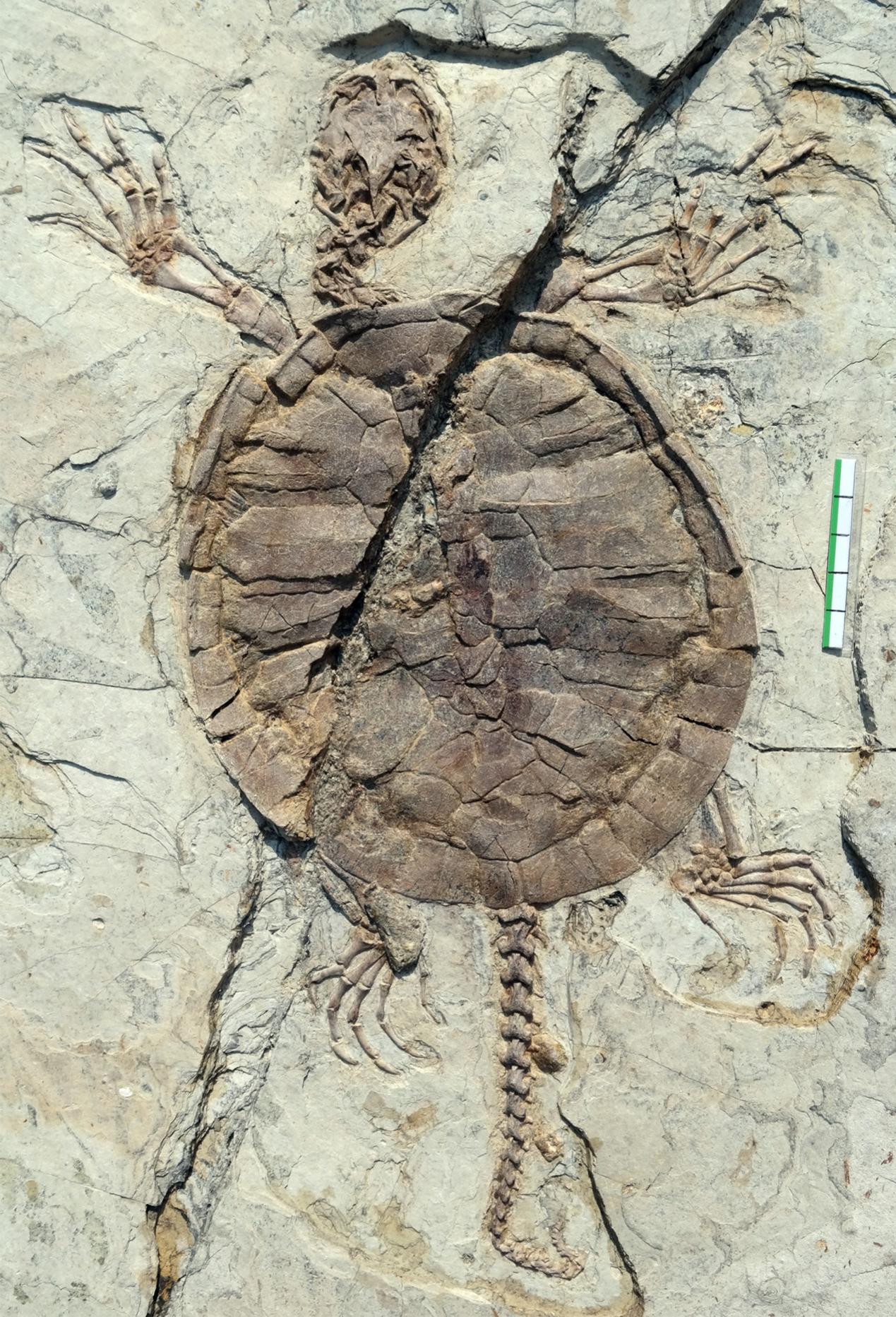
**

**Figure S5. SDUST-V1004 of *Liaochelys jianchangensis* Zhou, 2010a.**

**
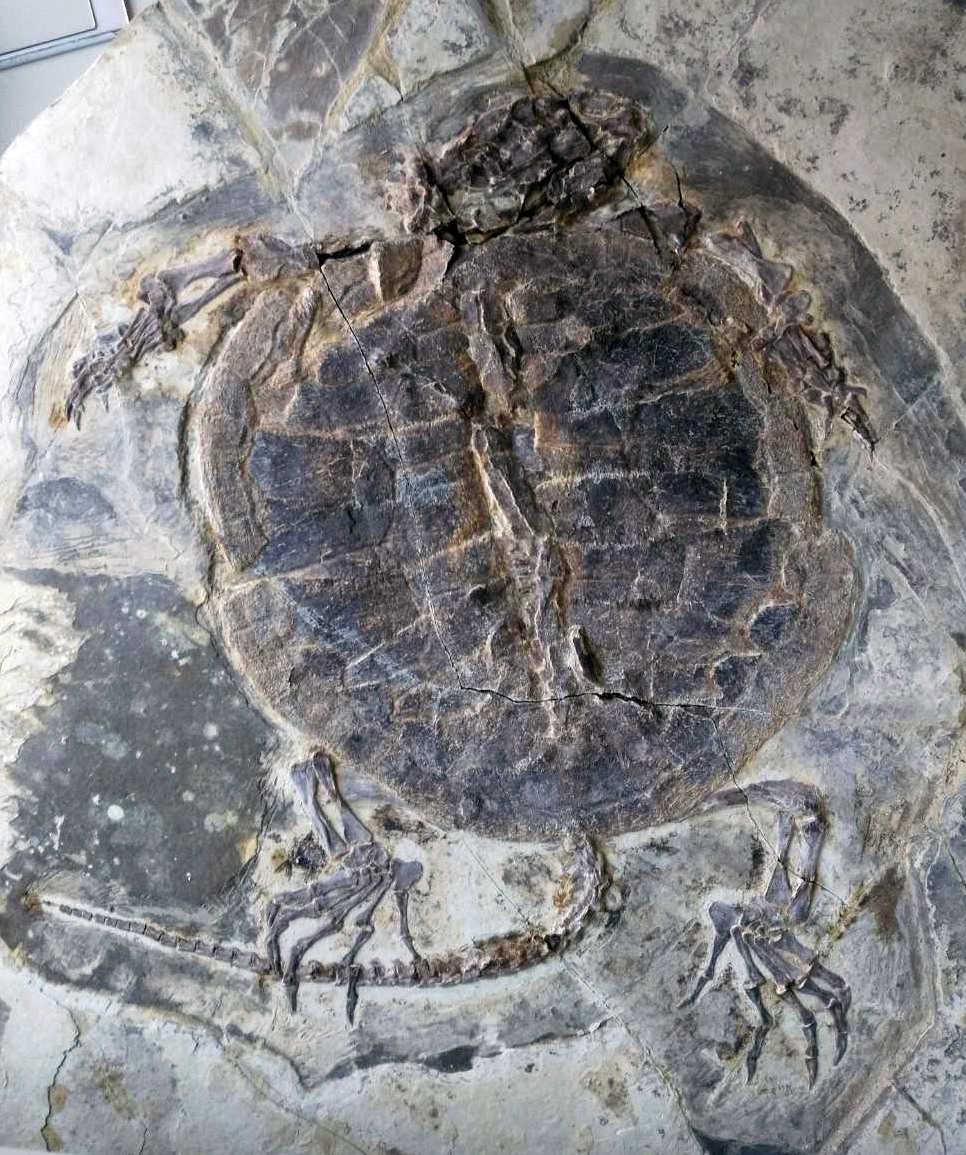
**

**Figure S6. SDUST-V1005 of *Liaochelys jianchangensis* Zhou, 2010a.**

**
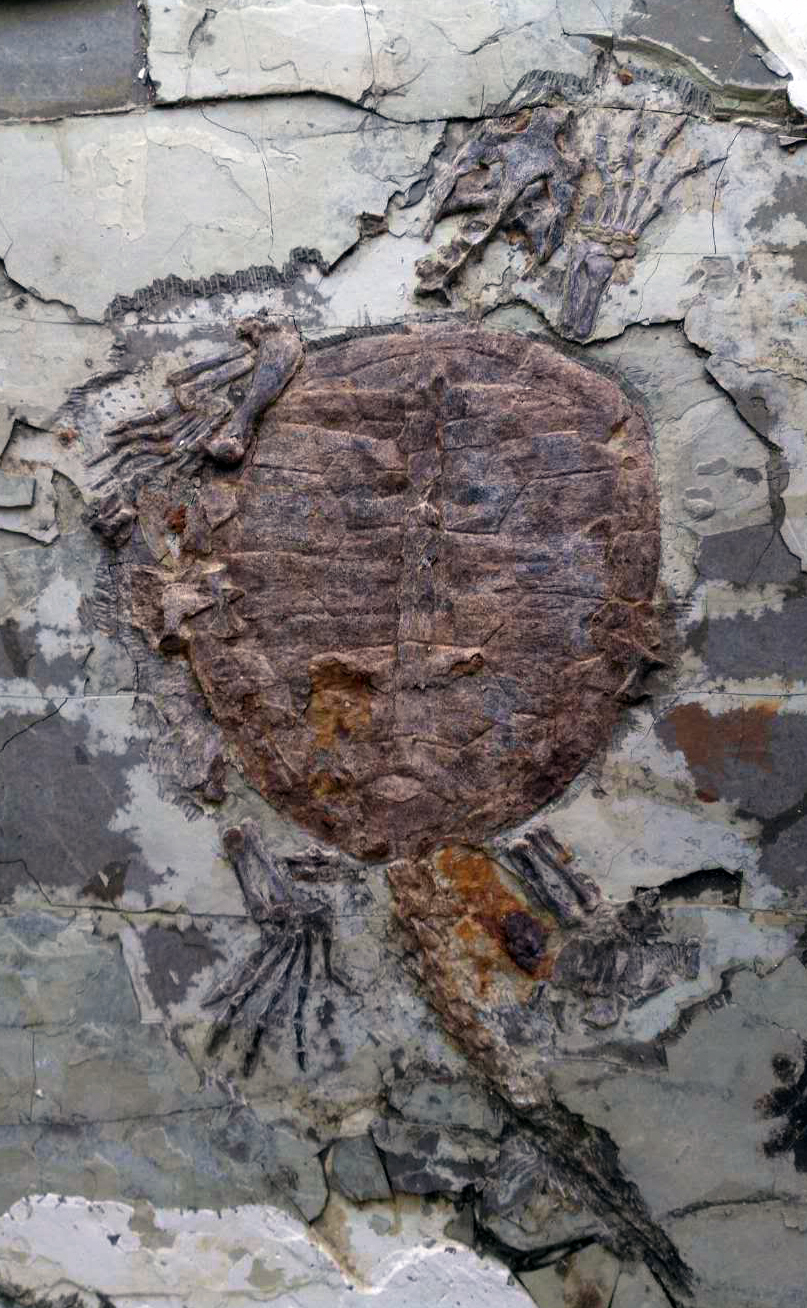
**

**Figure S7. SDUST-V1020 of *Ordosemys liaoxiensis* (Ji, 1995).**

**
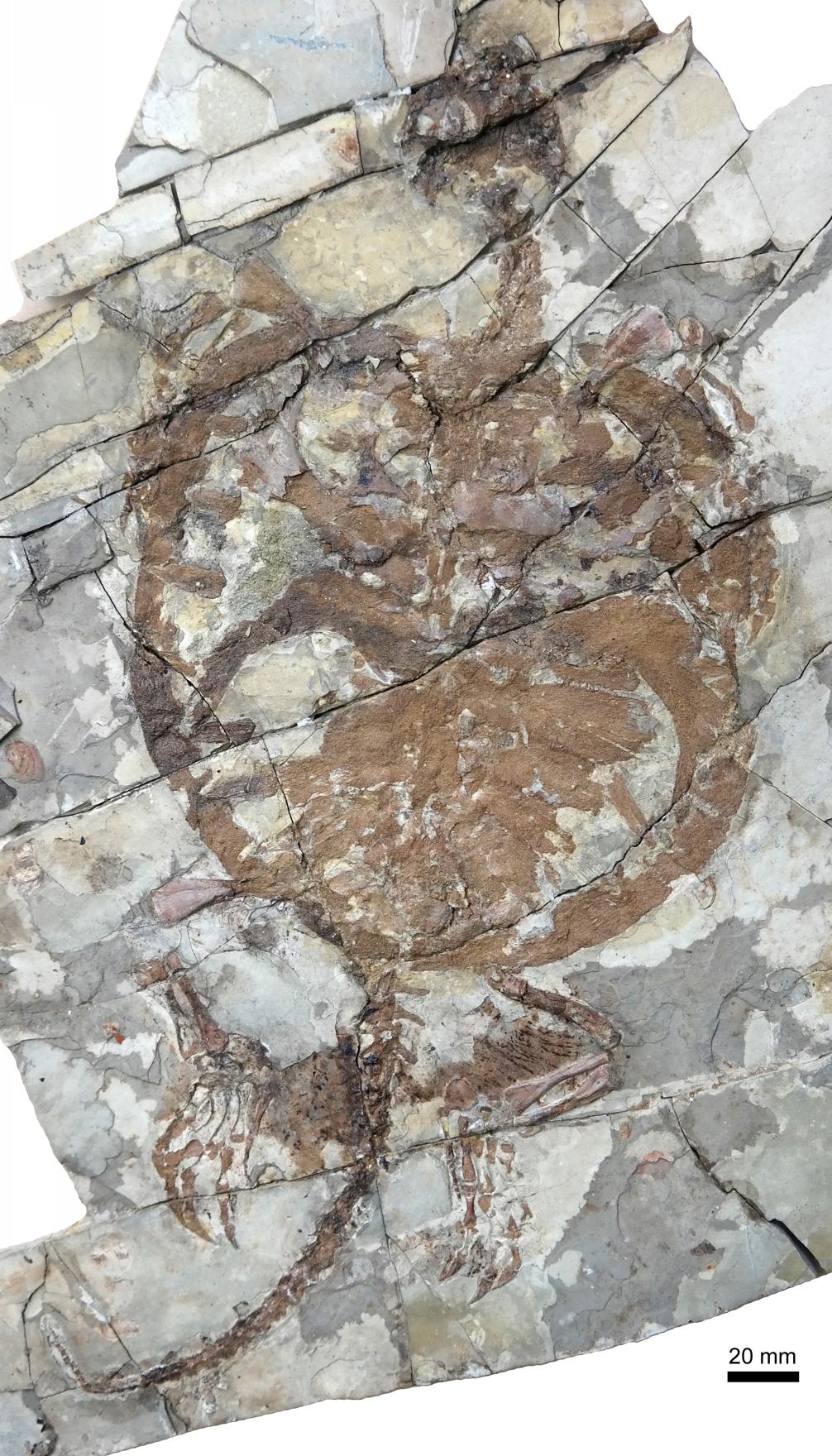
**

**Supplementary Data.**

**
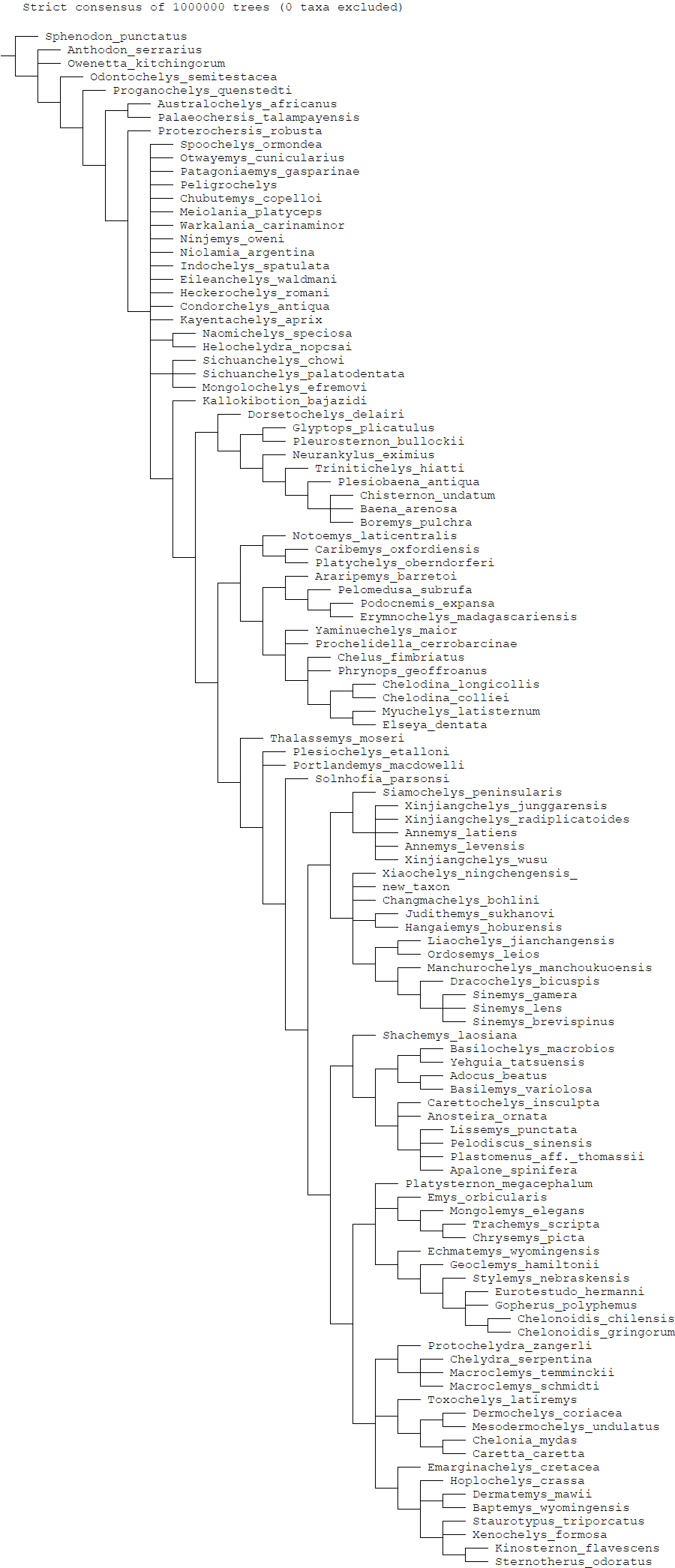
**

**Strict consensus tree**

**Synapomorphies of strict consensus tree**


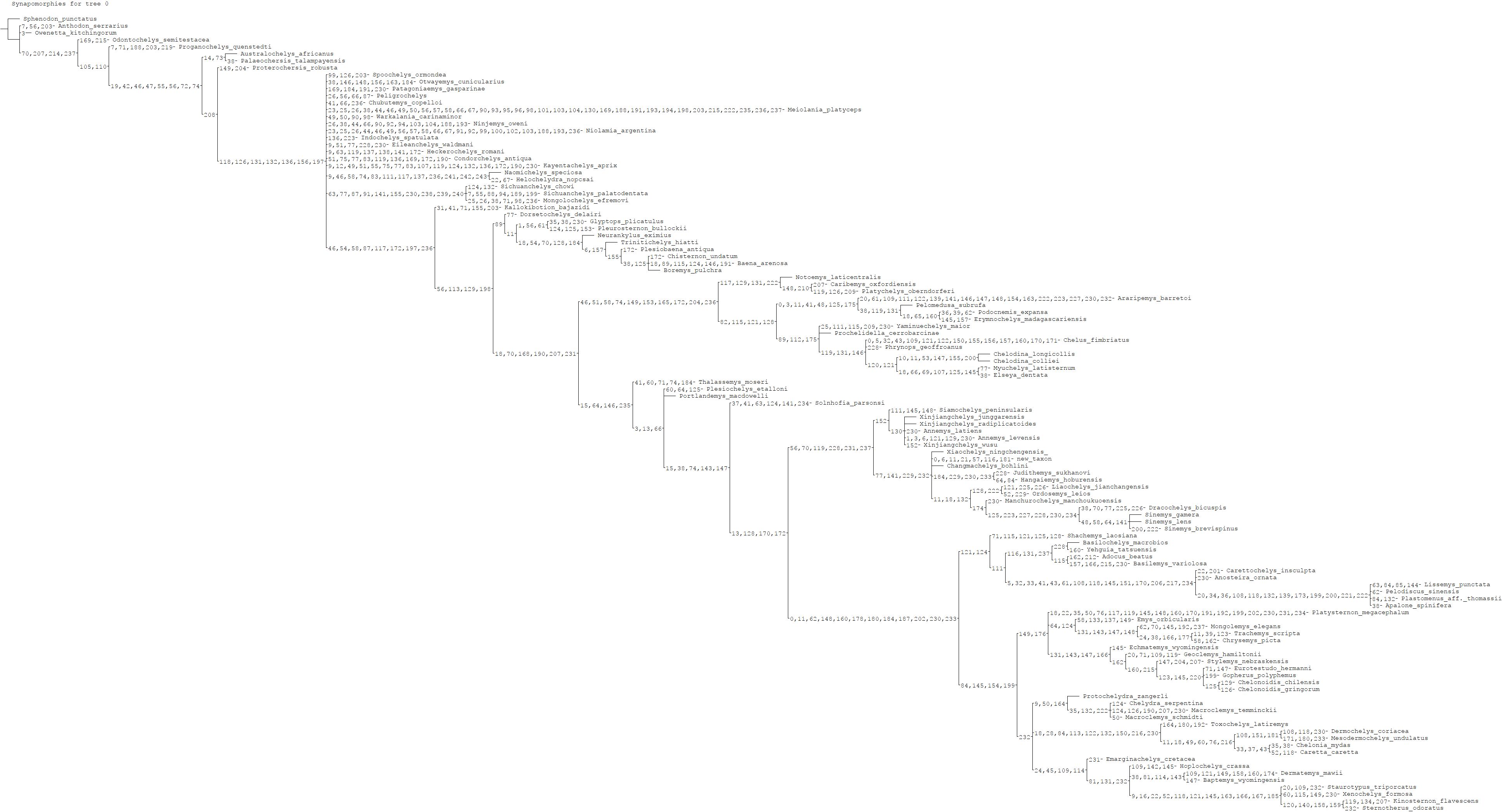


#NEXUS

[written Sat May 19 17:11:03 CST 2018 by Mesquite version 3.5 (build 888) at DESKTOP-DBPU9LT/192.168.0.106]

BEGIN TAXA;

TITLE Untitled_Taxa;

DIMENSIONS NTAX=114;

TAXLABELS

Sphenodon_punctatus Owenetta_kitchingorum Anthodon_serrarius Odontochelys_semitestacea Proganochelys_quenstedti Proterochersis_robusta Palaeochersis_talampayensis Australochelys_africanus Kayentachelys_aprix Condorchelys_antiqua Heckerochelys_romani Eileanchelys_waldmani Indochelys_spatulata Niolamia_argentina Ninjemys_oweni Warkalania_carinaminor Meiolania_platyceps Chubutemys_copelloi Peligrochelys Patagoniaemys_gasparinae Otwayemys_cunicularius Platychelys_oberndorferi Caribemys_oxfordiensis Notoemys_laticentralis Prochelidella_cerrobarcinae Elseya_dentata Myuchelys_latisternum Chelodina_colliei Chelodina_longicollis Phrynops_geoffroanus Chelus_fimbriatus Yaminuechelys_maior Araripemys_barretoi Erymnochelys_madagascariensis Pelomedusa_subrufa Podocnemis_expansa Dorsetochelys_delairi Pleurosternon_bullockii Glyptops_plicatulus Neurankylus_eximius Trinitichelys_hiatti Plesiobaena_antiqua Boremys_pulchra Baena_arenosa Chisternon_undatum Portlandemys_macdowelli Plesiochelys_etalloni Solnhofia_parsonsi Thalassemys_moseri Toxochelys_latiremys Caretta_caretta Chelonia_mydas Mesodermochelys_undulatus Dermochelys_coriacea Macroclemys_schmidti Macroclemys_temminckii Protochelydra_zangerli Chelydra_serpentina Platysternon_megacephalum Mongolemys_elegans Gopherus_polyphemus Eurotestudo_hermanni Chelonoidis_gringorum Chelonoidis_chilensis Stylemys_nebraskensis Chrysemys_picta Trachemys_scripta Emys_orbicularis Geoclemys_hamiltonii Echmatemys_wyomingensis Emarginachelys_cretacea Baptemys_wyomingensis Dermatemys_mawii Xenochelys_formosa Staurotypus_triporcatus Sternotherus_odoratus Kinosternon_flavescens Basilemys_variolosa Yehguia_tatsuensis Adocus_beatus Hoplochelys_crassa Apalone_spinifera Plastomenus_aff._thomassii Pelodiscus_sinensis Lissemys_punctata Shachemys_laosiana Anosteira_ornata Carettochelys_insculpta Xinjiangchelys_wusu Annemys_levensis Annemys_latiens Xinjiangchelys_radiplicatoides Xinjiangchelys_junggarensis Hangaiemys_hoburensis Changmachelys_bohlini Judithemys_sukhanovi Dracochelys_bicuspis Ordosemys_leios Sinemys_brevispinus Sinemys_lens Sinemys_gamera Liaochelys_jianchangensis new_taxon Basilochelys_macrobios Xiaochelys_ningchengensis_ Kallokibotion_bajazidi Mongolochelys_efremovi Sichuanchelys_palatodentata Sichuanchelys_chowi Helochelydra_nopcsai Naomichelys_speciosa Spoochelys_ormondea Siamochelys_peninsularis Manchurochelys_manchoukuoensis

;

END;

BEGIN CHARACTERS;

TITLE matriz_ppal;

LINK TAXA = Untitled_Taxa;

DIMENSIONS NCHAR=244;

FORMAT DATATYPE = STANDARD GAP = - MISSING = ? SYMBOLS = " 0 1 2 3 4 5 6 7 8 9";

CHARSTATELABELS

1 Nasal_A / present absent,

2 Nasal_B / nasals_contact_another_medially_along_their_entire_length medial_contact_of_nasals_partially_or_fully_hindered_by_long_anterior_fl,

3 Nasal_C / dorsal_exposure_of_nasal_large greately_reduced_relative_to_that_of_all_other_elements,

4 Prefrontal_A / medial_contact_on_dorsal_skull_roof_absent medial_contact_on_dorsal_skull_roof_present,

5 Prefrontal_B / 'prefrontal-vomer contact present' 'prefrontal-vomer contact absent',

6 Prefrontal_C / 'prefrontal-palatine contact present' 'prefrontal-palatine contact absent',

7 Prefrontal_D / prefrontal_exposure_large reduced absent_or_near_absent,

8 Prefrontal_E / prefrontal_heavily_sculptured_present absent,

9 Lacrimal_A / present absent,

10 Frontal_A / frontal_contribution_to_orbit_absent present,

11 'Frontal B*' / not_fused fused,

12 Parietal_A / 'parietal-squamosal contact present' absent,

13 Parietal_B / 'parietal contact with pt, epipt, and/or palatine absent' present,

14 Parietal_C / length_of_anterior_extension_of_the_lateral_braincase_wall_inter elongated 'short, enclosing the foramen nervi trigemini',

15 Parietal_D / overhanging_process_of_the_skull_roof_absent present,

16 Parietal_E / processus_inferior_parietalis_forming_posterior_margin_for_nerv_trigemini_absent ..._present,

17 'Parietal F*' / not_contribute_to_the_processus_trochlearis_oticum contributes_to_the_processus_trochlearis_oticum,

18 'Parietal G*' / 'forming part of the foramen stapedio-temporalis' not_forming,

19 'Parietal H*' / 'absent or weak, foramen stapedio-temporale concealed in dorsal view' 'moderate, f.s.t. but not entire processes trochlearis exposed in dorsal view' 'strong, entire processus trochlearis exposed in dorsal view',

20 Jugal_A / 'jugal-squamosal contact present' absent,

21 Jugal_B / jugal_participation_to_upper_temporal_rim_absent present,

22 Quadratojugal_A / present 'absent, due to the presence of a deep lower temporal emargination',

23 Quadratojugal_B / 'quadratojugal-maxilla contact absent' present,

24 Quadratojugal_C / 'quadratojugal-squamosal contact below cavum tympani absent' present,

25 Squamosal_A / 'squamosal-postorbital contact present' absent,

26 Squamosal_B / 'squamosal-supraoccipital contact absent' present,

27 'Squamosal C*' / posterolateral_protuberances_developing_horns_absent small_protuberances big_protuberances_developed_as_horns,

28 'Squamosal D*' / long_posterior_process_protruding_beyond_condylus_occipitalis_absent present,

29 'Squamosal E* ' / 'Qu-Sq contact tightly sutured' wide_open,

30 Postorbital_A / 'postorbital-palatine contact absent' 'present, foramen palatinum posterius situated posterior to the orbit',

31 Supratemporal_A / present absent,

32 Premaxilla_A / external_nares_divided united,

33 Premaxilla_B / fusion_of_premaxilla_absent present,

34 Premaxilla_C / foramen_praepalatinum_present 'absent, premaxillae well-ossified' 'absent, foramen intermaxillaris present',

35 Premaxilla_D / exclusion_of_premaxilla_from_the_apertura_narium_externa_absent present,

36 Premaxilla_E / 'distinct, medial premaxillary hook along the labial margin absent' present,

37 'Maxilla A*' / do_not_contact_each_other_in_ventral_view contacts_each_other_in_ventral_view,

38 'Maxilla C* ' / 'Secondary palate formed by premaxilla, maxilla, and vomer, palatines not contacting in midline absent' 'formed by premaxilla, maxilla, and vomer, palatines not contacting in midline present',

39 'Maxilla D* ' / Triturating_surface_with_only_labial_ridge_present labial_and_lingual_ridge_present 'labial, lingual and accesory ridges present',

40 'Maxilla E* ' / Accesory_ridge_on_maxilla_present_all_along_the_triturating_surface accessory_ridge_only_in_some_sectors_of_the_triturating_surface,

41 Vomer_A / paired single 'single, greatly reduced',

42 Vomer_B / 'vomer-pterygoid contact in palatal view present' 'absent, medial contact of palatines present',

43 Vomer_C / vomerine_and_palatine_teeth_present absent,

44 Vomer_D / 'vomer-premaxilla contact present' absent,

45 'Vomer E* ' / Narrow_and_tall_ventral_crest_on_vomer_absent present_all_along_the_vomer,

46 Palatine_A / palatine_contribution_to_anterior_extension_of_lat_braincase_absent 'present, well-developed',

47 Quadrate_A / flooring_of_the_cranioquadrate_space_absent 'by pt, but pt does not cover the prootic' by_pt by_qu_and_pro,

48 'Quadrate B + C' / 'development of the c.t. shallow, but not developed antpost' 'shallow, but anteroposteriorly developed' deep_and_anteroposteriorly_developed,

49 Quadrate_D / precolumellar_fossa_absent large_and_deep,

50 Antrum_postoticum_A / antrum_postoticum_absent incipient fully_developed,

51 'Quadrate F: incisura columella auris' / 'present, but qu and the op for an angle of 90 degrees in lat view' 'present, but qu and the op for an angle less 90 degrees in lat view' 'present and closed, but only enclosing the stapes' 'present and closed, enclosing stapes and the Eustachian tube' 'partially closed, allowing see the columella auris in posterior view',

52 Quadrate_G / processus_trochlearis_oticum_absent present,

53 'Quadrate H* ' / Processus_trochlearis_oticum_formed_by_a_great_contribution_of_quadrate small_contribution_of_the_quadrate,

54 'Quadrate I*' / 'Quadrate-basisphenoid contact absent' present,

55 Epipterygoid_A / 'present, rod-like' 'present, laminar' absent,

56 Pterygoid_A / pterygoid_teeth_present absent,

57 Pterygoid_B / basipt_process_present_and_movable_articulation basipt_process_present_and_sutured_articulation basipt_process_absent_and_sutured_articulation,

58 Pterygoid_C / triangular_in_shape reduced_to_an_interpterygoid_slit reduced_to_a_paired_foramen_caroticum_laterale,

59 Pterygoid_D / 'pterygoid-basioccipital contact absent' present,

60 Pterygoid_E / processus_trochlearis_pterygoidei_absent present,

61 Pterygoid_F / foramen_palatinum_posterius_present 'present, but open laterally' absent,

62 Pterygoid_G / medial_contact_of_pterygoids_present absent,

63 Pterygoid_H / pterygoid_contribution_to_foramen_palatinum_posterius_present absent,

64 Pterygoid_I / vertical_flange_on_lateral_process_absent vertical_falnge_on_lateral_process_present,

65 'Pterygoid J*' / not_reaching_the_exoccipitals reaching_the_exoccipitals,

66 'Pterygoid K*' / Fossa_podocnemidoidea_absent present,

67 Supraoccipital_A / crista_occipitalis_poorly_developed protruding_significantly_posterior_to_the_foramen_magnum,

68 Supraoccipital_B / large_supraoccipital_exposure_to_dorsal_skull_roof_absent present,

69 'Supraoccipital C*' / horizontal_ventral_crest_in_the_supraoccipital_absent_or_poorly_developed_anteriorly horizontal_ventral_crest_present_along_all_the_crista_supraoccipitalis,

70 Exoccipital_A / medial_contact_of_exoccipitals_dorsal_to_foramen_magnum_absent present,

71 Basioccipital_A / with_two_or_one_ventral_basioccipital_tubercle tubercle_absent,

72 'Prootic A*' / dorsal_exposure_large dorsal_exposure_reduced_or_absent,

73 Opisthotic_A / loosely_articulated tightly_sutured,

74 Opisthotic_B / depressions_for_musculature_absent present,

75 Opisthotic_C / ventral_ridge_on_opisthotic_absent 'present, with an incipient enclosed middle ear region' 'present, but modified with a enclosed middle ear region',

76 'Opisthotic D: processus interfenestralis' / 'present, robust, not reaching the floor of cavum a-j' 'present, robust, reaching the floor of cavum a-j' 'present, small, reaching the floor of cavum a-j',

77 Basisphenoid_A / rostrum_basisphenoidale_flat 'rod-like, thick, and rounded',

78 Basisphenoid_B / paired_pits_on_ventral_surface_absent present,

79 'Basisphenoid C*' / 'reduced to a v-shaped basisphenoid trapped between the pterygoids and the basioccipital absent' 'reduced to a v-shaped basisphenoid trapped between the pterygoids and the basioccipital present',

80 Hyomandibular_nerve_A / ': path of hyomandibular branch facial nerve through cranio-quadrate space parallel to vena capitis lateralis' independent_to_vena_capitis_lateralis,

81 Stapedial_artery_A / posterior_to_fenestra_ovalis_between_paraoccipital_process_and_qu anterior_to...,

82 Stapedial_artery_B / relatively_large significantly_reduced_in_size absent,

83 'Stapedial artery C* ' / 'Foramen stapedio-temporalis located in the dorsal part of the otic region and points dorsally' located_in_the_anterior_wall_of_the_otic_region_and_points_anteriorly,

84 'Recessus scalae tympani A*' / 'almost inexistent, not surrounded by bone' well_developed,

85 'Foramen jugulare posterius A*' / separated_from_fenestra_postotica coalescent_with_fenestra_postotica,

86 'Foramen jugulare posterius B*' / separated_from_fenestra_postotica_by_pterygoid separated_by_opisthotic_and_or_exoccipital,

87 'Foramen nervi hypoglossi (XII)*' / not_covered_ventrally_by_an_extension_of_the_pterygoid_and_the_basioccipital covered_ventrally_by_an_extension_of_the_pterygoid_and_the_basioccipital covered_ventrally_by_an_extension_of_the_bo,

88 'Canalis caroticum F* ' / Arteria_palatina_enters_the_skull_through_the_interpterygoid_vacuity_or_intrapterygoid_slit through_foramen_posterius_canalis_carotici_palatinum_or_split_of_branches_enclosed_in_skull,

89 Fenestra_perilymphatica_A / large relatively_small,

90 'Cranial scutes A*' / present absent,

91 'Cranial scute B*' / Scute_D_meeting_in_midline_no yes,

92 'Cranial scute C*' / Scute_X_much_smaller_than_D_scute_no yes,

93 'Cranial scute D*' / X_scute_partially_separates_G_scales_no yes,

94 'Cranial scute E*' / 'Scutes A, B, and C forming a continuous posterolateral shelf yes' no,

95 'Cranial scute F*' / D_scute_high low,

96 'Cranial scute G*' / B_scute_a_recurved_horn_no yes,

97 'Cranial scute H*' / B_scute_in_cross_section_triangular round,

98 'Cranial scute I*' / Scute_B_and_D_in_contact_yes no,

99 'Cranial scute J*' / A_scute_small_and_not_forming_a_large_shelf_no yes,

100 'Cranial scute K*' / A_scute_small_A_scute_very_large A_scute_comparable_in_size_to_B_scute,

101 'Cranial scute L*' / Y_and_Z_scutes_relatively_larges_mall large,

102 'Cranial scute M*' / Y_scute_pentagonal_pointing_posteriorly_and_separating_the_medial_contact_of_G_scutes rectangular_not_separating_the_medial_contact_of_G_scutes,

103 'Cranial scute N*' / H_scute_present absent,

104 'Cranial scute O*' / Scale_F_formed_by_several_scales Scale_F_formed_by_only_one_scale,

105 'Cranial scale P*' / Scale_J_formed_by_several_scales Scale_J_formed_by_only_one_scale,

106 Teeth_A / 'teeth present in premaxilla, maxilla, and dentary' 'teeth absent in premaxilla, maxilla, and dentary',

107 Upper_temporal_fenestra_A / present absent,

108 Dentary_A / medial_contact_of_dentaries_fused sutured_only,

109 Carapace_A / carapacial_scutes_present partially_present absent,

110 Carapace_B / tricarinate_carapace_absent 'present, but only slightly' present_and_pronounced,

111 Carapace_C / absent present,

112 'Carapace D *' / Sculpturing_of_the_shell_absent present,

113 'Carapace E*' / Sculpturing_of_the_shell_like_in_Hydromedusa like_in_Pleurosternon like_in_trionychians,

114 Nuchal_A / cervical_articulates_with_nuchal_along_a_blunt_facet articulation_absent cervical_articulates_with_nuchal_along_a_raised_pedestal,

115 Nuchal_B / elongate_costiform_process_of_nuchal_absent 'present, process crosses peripheral I to contact pe II ' 'present, contacts pe III',

116 'Nuchal C*' / wider_than_long longer_than_wide_or_as_long_as_wide,

117 Neural_A / 'neural formula 6>4<6<6<6<6 absent' present,

118 'Neural B*' / 'irregular in shape, wider than long' 'regular, often hexagonal, longer than wide',

119 Peripheral_A / more_than_11_pairs 11_pairs 10_pairs less_than_10_pairs,

120 'Musk ducts A*' / absent present,

121 Costal_A / medial_contact_of_costal_I_absent present,

122 Costal_B / medial_contact_of_posterior_costals_absent medial_contact_of_up_to_three_posterior_costals_present medial_contact_of_all_costals_present,

123 Costal_C / 'absent, costals fully or almost fully ossified, fontanelles abs or red' present,

124 'Costal D*' / absence_of_alternative_short_and_long_ends_in_the_lateral_part_of_the_costals presence,

125 'Suprapygal A*' / none one_element two_elements more_than_2_elements,

126 Cervical_A / 'cervicals absent, carapacial scutes otherwise present' one_cervical_present more_than_one_cervical_present,

127 Supramarginal_A / complete_row_present partial_row_present absent,

128 Vertebral_A / 4 5,

129 Vertebral_B / 'vertebral II-IV broader than pleurals' 'vertebrals II-IV narrower or as narrow as pleurals',

130 Vertebral_C / sulcus_between_V_3_and_4_on_neural_VI on_neural_V,

131 'Marginal A*' / marginal_scales_overlap_onto_costals_absent present,

132 Plastron_A / connection_between_carapace_and_plastron_osseous ligamentous,

133 Plastron_B / central_plastral_fontanella_absent present,

134 Plastron_C / plastral_kinesis_absent present,

135 'Plastral kinesis A*' / anterior anterior_and_posterior,

136 'Plastral kinesis B*' / between_hyo_and_hypoplastron 'between hyo and epi-entoplastron',

137 Entoplastron_A / anterior_entoplastral_process_present absent,

138 Entoplastron_B / size_of_posterior_entoplastral_process_long short,

139 Entoplastron_C / distinct_posterolateral_entoplastral_process_present absent,

140 Entoplastron_D / 'entoplastron V-shaped absent' present,

141 Entoplastron_E / present absent,

142 Epiplastron_A / epiplastra_and_entoplastron_narrow_and_elongate_absent present,

143 'Epiplastron B*' / thick_anterior_border thick_anterior_border_absent,

144 Hyoplastron_A / axillary_buttresses_contact_peripherals_only peripherals_and_first_costal,

145 'Hyo-hypoplastron A*' / not_fused fused,

146 'Hyoplastron B*' / Axillary_buttress_terminates_on_peripheral_2_or_1 terminates_on_peripheral_3 terminates_on_peripheral_4,

147 Mesoplastron_A / 1_or_2_pairs_of_meso_with_medial_contact 1_reduced_pair absent,

148 Hypoplastron_A / inguinal_buttresses_contact_peripherals_only peripheral_and_costal_V 'peripherals, costal V, and costal VI',

149 'Hypoplastron B*' / Inguinal_buttress_terminates_on_peripheral_8 7 6,

150 Xiphiplastron_A / distinct_anal_notch_absent present,

151 Xiphiplastron_B / xiphiplastra_narrow_absent present,

152 Plastral_scutes_A / present absent,

153 Plastral_scutes_B / pronounced_midline_plastral_sulcus_sinuous_absent present,

154 Gular_A / one_pair only_one_scute,

155 Extragular_A / present absent,

156 Extragular_B / medial_contact_of_extragulars_absent 'present, contacting one another anterior to gulars' 'present, contacting one another posterior to gulars',

157 Extragular_C / anterior_plastral_tuberosities_present absent,

158 'Extragular D*' / Only_in_the_epiplastra Reach_the_entoplastron,

159 Intergular_A / absent present,

160 Humeral_A / 1_pair 2_pair_subdivided_by_a_plastral_hinge,

161 'Humeral B*' / 'Humero-pectoral sulcus only in the hyoplastra' 'humero-pectoral sulcus crossing the entoplastron',

162 Pectoral_A / present absent,

163 'Pectoral B*' / 'antero-posteriorly developed' 'very short antero-posteriorly',

164 Abdominal_A / 'present, with medial contact' 'present, medial contact absent' absent,

165 Anal_A / only_cover_parts_of_the_xiphiplastra anteromedially_overlap_onto_hypoplastra,

166 Inframarginal_A / present absent,

167 'Inframarginal B*' / 3_or_more 2,

168 'Inframarginal C*' / axillar_and_inguinal_not_in_contact axillar_and_inguinal_in_contact,

169 Cervical_rib_A / present absent,

170 Cervical_vertebra_A / position_of_transverse_processes_middle_of_the_centrum anterior_end_of_the_centrum,

171 Cervical_vertebra_B / ventral_keels_absent_or_slightly_developed_in_all_vertebrae ventral_keels_more_developed_on_posterior_vertebrae,

172 Cervical_vertebra_C / 'cervical centrum 8<7 absent' present,

173 Cervical_articulation_A / not_formed formed,

174 Cervical_articulation_H / '8(dorsal' '8)dorsal' 'none, vertebrae only meet at zygapophyses',

175 'Cervical vertebra E*' / Biconvex_cervical_vertebra_in_the_middle_of_the_neck_absent present,

176 'Cervical vertebra F*' / Biconvex_cervical_vertebra_in_the_middle_of_the_neck_2nd 3rd 4th 5th,

177 'Cervical vertebra G*' / Biconcave_cervical_vertebra_absent present,

178 'Cervical articulation I*' / double_articulation_between_5th_and_6th_absent present,

179 'Cervical articulation J*' / double_articulation_between_6th_and_7th_absent present,

180 'Cervical articulation K*' / 'Central articulation cervical 6-7 concave-convex' platicoelous,

181 'Cervical articulation L*' / double_articulation_between_7th_and_8th_absent present,

182 'Cervical vertebra H*' / total_height_of_centra_and_neural_arch_longer_than_the_anteroposterior_length_of_the_cervical_centra_ total_height_of_centra_and_neural_arch_much_shorter_than_the_anteroposterior_length_of_the_cervical_centra_,

183 'Cervical vertebra I*' / neural_arch_on_8th_cervical_not_modified 'neural arch on 8th cervical modified with the postzygapophyses articular surface greatly expanded AND/OR pointing posteroventrally ',

184 'Cervical vertebra J*' / postzygapophyses_not_united_in_midline postzygapophyses_united_in_midline,

185 Dorsal_rib_A / 'length first thoracic rib long, extends full legth of first costal and may contact peripherals' 'intermediate, in contact with axillary buttresses' intermediate_to_short,

186 Dorsal_rib_B / 'contact dorsal rib 9-10 with costals present' absent,

187 Dorsal_rib_C / 'dorsal rib X long, contacting peripherals' dorsal_rib_X_short,

188 Dorsal_vertebra_A / anterior_articulation_of_first_dorsal_centrum_faces_at_most_slightly_anteroventrally faces_strongly_anteroventrally,

189 Caudal_A / tail_club_present absent,

190 Caudal_B / all_centra_amphicoelous formed_centra,

191 'Caudal C*' / anterior_caudal_vertebrae_amphicoelous anterior_caudal_vertebrae_procoelous_or_platycoelous anterior_caudal_vertebrae_opisthocoelous,

192 'Caudal D*' / posterior_caudal_vertebrae_amphicoelous posterior_caudal_vertebrae_procoelous_or_platycoelous posterior_caudal_vertebrae_opisthocoelous,

193 Chevron_A / present_on_nearly_all_caudals absent_or_poorly_developed_along_posterior_caudals,

194 'Tail ring A*' / absent present,

195 'Tail ring B*' / closed_ventrally open_ventrally,

196 'Tail club A*' / with_three_spikes with_two_pairs_of_spikes_,

197 Pectoral_girdle_A / 'horizontal plate with a dorsal process, not triradiate' trirradiate,

198 Cleithrum_A / present_and_in_contact_with_the_carapace 'present, osseous contact with carapace absent' absent,

199 'Scapula A*' / lamina_between_the_dorsal_process_of_the_scapula_and_the_acromion_well_developed 'lamina between the dorsal process of the scapula and the acromion reduced: Kallokibotion' lamina_between_the_dorsal_process_of_the_scapula_and_the_acromion_absent,

200 'Humerus A*' / Ectepicondylar_foramen_in_a_channel only_a_groove,

201 'Humerus B*' / shoulder_present 'shoulder absent: pleurodires',

202 'Humerus C*' / lateral_process_in_the_proximal_end_of_the_humerus 'displaced from the proximal end, located in the shaft of the humerus',

203 'Humerus D*' / lateral_process_seen_in_dorsal_view lateral_process_not_seen_in_dorsal_view,

204 'Humerus E*' / length_of_the_humerus_two_times_or_less_than_the_width_of_the_proximal_end length_of_the_humerus_more_than_two_times_the_width_of_the_proximal_end,

205 Pelvis_A / 'pelvis-shell attachment by ligaments' ischium_attached_to_plastron_by_a_broad_suture ischium_attached_to_plastron_by_its_medial_surface,

206 'Pelvis B*' / Thyroid_fenestra_coalescent two_separated_fenestra_completely_or_partially_separated,

207 'Pubis A*' / 'lateral process small, poorly developed, columnar' lateral_process_well_developed_and_flat,

208 'Pubis B*' / Epipubis_process_osseous_or_calcified cartilaginous_or_absent,

209 Ilium_A / elongated_iliac_neck_absent present,

210 Ilium_B / iliac_scar_extends_from_costals_onto_the_peripherals_and_pygal positioned_on_costals_only,

211 Ilium_C / shape_of_articular_site_narrow_and_pointed_posteriorly oval,

212 Ilium_D / posterior_notch_in_acetabulum_absent present,

213 'Illium E*' / thelial_process_absent present,

214 'Ischium A*' / with_lateral_processes_absent with_lateral_processes_present,

215 Hypoischium_A / present absent,

216 Manus_A / most_digits_with_two_shortened_phalanges most_digits_with_three_elongate_phalanges,

217 Manus_B / paddles_absent short_paddles_present elongate_paddles_present,

218 Manus_C / flippers_absent short_flippers_present elongate_flippers_present,

219 Pes_A / claw_on_5th_digit_present absent,

220 Pes_B / metatarsal_V_functions_as_true_metatarsal metatarsal_V_functions_as_a_tarsal,

221 'Pes C*' / 5_digits 4_digits,

222 'Manus and Pes B*' / 'Hyperphalangy manus digits 4 and 5, pes digit 4 no' yes,

223 Posterior_plastral_fontanelle / 'posterior plastral fontanella between the xiphiplastra and/or the hypoplastra: absent in adult stage' retained_in_adults,

224 Neural_number / less_than_9_elements 9_elements,

225 Plastron_lobe / posterior_lobe_of_plastron__relatively_wide_and_short_ posterior_lobe_of_plastron_elongated_and_narrow_coupled_with_widely_spaced_plastral_buttresses._,

226 Shape_of_costal_3 / costal_3_tapering_towards_the_lateral_side_of_the_shell_or_with_parallel_anterior_and_posterior_borders costal_3_broadens_towards_the_lateral_side_of_the_shell,

227 Costal_rib / distal_portion_of_costal_ribs_not_visible_within_the_costal distal_portion_of_costal_rib_visible_on_the_surface_of_the_costal,

228 First_vertebral / vertebral_1_does_not_enter_anterior_margin_of_carapace enters_anterior_margin,

229 Peripheral_gutter / peripheral_gutter_absent_or_only_anteriorly_developed peripheral_gutter_extensively_developed_along_anterior_and_bridge_peripherals,

230 Costal_rib_distal_end / 'distal end of dorsal rib not visible or only within costo-peripheral fontanelles on the dorsal face of the carapace' 'costo-peripheral fontanelles absent, distal end of posterior dorsal ribs visible or distal end of posterior costals narrow and surrounded by the peripheral',

231 Nuchal_emargination / absent_or_indistinct 'present, excludes peripheral 1' deep_and_involves_peripheral_1 'broad, involved peripheral II',

232 Tail_length / tail_as_long_as_carapace tail_clearly_shorter_than_carapace,

233 Cruciform_plastron / absent present,

234 Articulation_of_posterior_cervical_centra / circular_or_subcircular_outline greatly_flattened_outline,

235 Nuchal_posterior_edge / less_than_3_times_longer_than_the_lateral_edge more_than_3_times_longer,

236 Carotid_canal_entry / fpcci_is_not_at_back_of_skull fpcci_located_at_back_of_skull_in_pterygoid,

237 Pterygoid_extension / _pterygoid_not_extending_to_posterior_end_of_skull_and_covering_prootic pterygoid_extending_to_posterior_end_of_skull_and_covering_prootic,

238 Carotid_canal_split / not_enclosed_in_bone not_enclosed_but_carotid_canal_is_covered_ventrally_from_the_posterior_end_of_the_skull enclosed_but_carotid_canal_is_not_covered_ventrally_from_posterior_edge_of_skull,

239 Antrum_postoticum / region_of_antrum_postoticum_enlarged_and_laterally_enclosed 'region of antrum postoticum enlarged, but not enclosed laterally',

240 'Jugal/quadrate contact' / jugal_clearly_not_in_contact_with_quadrate jugal_nearly_or_clearly_in_contact_with_quadrate,

241 Parabasisphenoid_decorated_by_ridges / absent present,

242 Entoplastral_scute / absent present,

243 Secondary_pair_of_basioccipital_tubercles_formed_by_pterygoid / absent present,

244 Shell_covered_by_highly_distinct_turbercles / absent present ;

MATRIX

Sphenodon_punctatus 0000100101000-0----0-00-000-10100-00?---0000000----0-001000000000-00-010000-100-???0--01?1---------------000--0---------------------------------------------------------010-0--------0-0----100000--0--??0?1-1-10--0011000010----------0-0-00{2 3}-0?-0-

Owenetta_kitchingorum 000100010102?-0--?0000000000?0000000?---0000?0000000-0?000000000?-00-?-?0000000????0--01?1---------------010--0---------------------------------------------------------0??-?--------?-0----1???00--0--??0?1-???0--00?1???????---------0-0-00{2 3}-0?-0-

Anthodon_serrarius 0000??0000020-0--?0000000000000000000---0000000000-0-?00100000000-00001?0000000????0--01?1---------------010--0---------------------------------------------------------?????--------?-0----100000--0--01--0-1-10--00?1?00????---------?-0-00{2 3}-0?-0-

Odontochelys_semitestacea 0000??01?000??0?-?0?00??0?000?00??00?0--??0???0000?0-0?00000?0??0000-?0???????0????0-????????????????????010--0-----00-------------100--0??000?-0-0--00?????????????????000?0--------?00----100000--0-0??0010100?--???0100???100001?---000-000-?0000

Proganochelys_quenstedti 0000?00000000-0--?000000000000000000001-000000000000-0?0000000000000-001000000000?00--0000011110-011100001100010-00??000000??-000-?000--000000?00?00?0000000010000000???01000--------0000000000000-00000000001000--00?000000000?0000001000?000-00000

Proterochersis_robusta ????????????????????????????????????????????????????????????????????????????????????????????????????????????0010-?????00??0??-110??000--001000?00?00?100000000000000000-?????????????????????????????0??????1?0?1??00?????????00000000??0???0?-??0?0

Palaeochersis_talampayensis 0000?00100000-1--?010000000000000?00?00-0?1?00110000-0?1100000000000-00?1110?00????0--00?????????????????1100010-?0????0??0??-?????000--001000?00?00?0000???0???????0???11000--------00000?0100000--0000?00121000???0?0000???000000000000??000-00000

Australochelys_africanus ???????10??0??1--?0?00?00?000??0??00?01-?0??0?110000-0??1000000?000?-???1110?00????0--00?????????????????11????????????????????????????????????????????????????????????????????????????????????????????????????????????????????????????????000-?0???

Kayentachelys_aprix 0010001111000-0--?010000000000?10?00001-101000120110-000100000000000-0001011010?1000--0000???????????????1110010-00000(0 1)100001121000100--001000?000000000000010000000000-0?0?0--------??000001?0000--11?0?00?01001--00?1?00????00000000200?0000?00000

Condorchelys_antiqua ??????????????0?-?0???????000?????????????????120?10-0?11000?0?000??-?0?1011?10????0--00??????????????????1?0010-????0?1?0002???00??10--00100??????????0???????0?0??????010?0?-??----0?0?0??1?00?0--1??0000?0100????0?1????????0?00000?00??000???0?0

Heckerochelys_romani 0??00???1100??0?-?01????0??0???1????????1?1???12??1??0?11000?0?1000?-???10120?0????11-00????????????????????0010-00000?10?0021?10?0110--110001?000000000000010000000000-????0--------??????0?????0--?1??????0????--???1???????000000000?0?0000??0000

Eileanchelys_waldmani 00000?111100??0?-10100000?0000?1000000??1?100?1202?0-0?1??00????000?-?001012?10??0011-0??????????????????1100?10-??0001?0000212100???0--1??000??0?0??000?00???00?0???0???????????????????????????????1????????????????????????000000102?0?0000?00000

Indochelys_spatulata ????????????????????????????????????????????????????????????????????????????????????????????????????????????0010-??000??00002?2100???0--0??000??0?0??000????1?????????????????????????????????????????????????????????????????010000000?0?0??????0?0

Niolamia_argentina 000000011000??0?010100010120?0110?00001-1010102200?100(0 1)1211000?000110000101??001??01??00?00000(0 1)00(0 1)000(0 1)1101100?10-???????????????????????????????????????????????????????????????????????????0????101????????????????????????????????????0??01?0?0?0?

Ninjemys_oweni 0???????1?????0???0?0?????20???1??00?0211?101??2????????????0?????1?0????????????????????011000000011001111?????????????????????????????????????????????????????????????????????????????????0????101??????????????????????????????????????????0?0?0?

Warkalania_carinaminor ??????????????????0????????????????????????????20031?????????????????????????????????????011101--011??????????????????????????????????????????????????????????????????????????????????????????????????????????????????????????????????????????0?????

Meiolania_platyceps 0000000110001-000101000101200011000000211010102200310001211000(0 1)000110000101200011001(0 1)?00001111111011110111100010-000??10000??1210?1110--101000?0?1?0000000001000????????010011120000000000?0012201111100000001001--00??000??001?0?000000000111000000

Chubutemys_copelloi ???0000110001100??0?000?00?00?11?????0??11100?120211?0?112000000001?-?0?1012000????11-0??????????????????11?0?1?????????0????????????????????????????????????????????????????1?????????0???0????????1???????????????????????????00000?0?0?0010000000

Peligrochelys ???0?????00?????01????????1?0?????????1-???????20?1100(0 1)?220?????0?1???001012?00110011-01?0???????????000011????????????????????????????????????????????????????????????????????????????????????????????????????????????????????????????????0?0??????

Patagoniaemys_gasparinae ????????????????????????????????????????????????????????????????????????????????????????????????????????????0010-000?0??0?00?1?10??110--???????0???0???0???????0?00?????010?1112100000?01??0112100--????????0?0?1--00?1???????????????20?0???0??????

Otwayemys_cunicularius ??????????????????(0 1)???????????????????0-?????????2?1????????????????-?????????????0????????????????????????0001???0????00?00??2?0??110--1???0???0?202000000000000001?00-00??11??10???0001??0?12?0?????????????????????????????0?0?0??0??0????????0?0

Platychelys_oberndorferi ????????????????????????????????????????????????????????????????????????????????????????????????????????????0010-100001100001111000010--111000?10111110001001100000001--110?11???----1?00010?????0--????????11?110000?????????100000000?0?0??????0?0

Caribemys_oxfordiensis ????????????????????????????????????????????????????????????????????????????????????????????????????????????001??10000?0??00??2????010--11?000?101111?00010011000000?1--1???1????----??0?0???????0--?????0?11100?10?0???????????000??0??0????????0?0

Notoemys_laticentralis ????????????????-?????????000?????????????????3202?0-0??2?0?????00???0101?02?00?10011-01????????????????????0010-100001000002121000010--11?000?1011?010001001?00000001--110?1?-------1?0201??????0--?????0011????11?0??????1??100000000?0?00020?0000

Prochelidella_cerrobarcinae ????????????????????????????????????????????????????????????????????????????????????????????????????????????0010-??1011001001121110100--1110001?0?1??10001001000000001--????????????????????????????????????????????0?????????000000001?000??????0?0

Elseya_dentata 0110111111001100-?0101--1000011100001020101100320230-021220100000010-0101002000110111-?101---------------110001??10?0-1?1200?0211-0000--111000?10021010001001?00?00001--10?011131000010?201011??10--122??0??1???11100?110011??0-000000010000020-0000

Myuchelys_latisternum 0110111111001100-10101--100001110000100-101100320230-021220100000010-0101002010110111-0101---------------110001??1010-1?120010211-0000--111000110021110001001000000001--10001113100001002010111110--122?00011001111000110011??0-000000010000020-0000

Chelodina_colliei 0110111111111100-12101--100001110000100-101?00320230-12122010000000001101002000110111-2101---------------11100110101001?120011211-0000--111000110120110001011000000001--10001113100001012010111110--1221100110011110001100110?00000000010000020-0000

Chelodina_longicollis 0110111111111100-12101--100001110000100-101100320230-12122010000000001101002000110111-2101---------------111001101010-11120011211-0000--111000110120110001011000000001--10001113100001012010111110--122110011001111000110011000-000000010000020-0000

Phrynops_geoffroanus 0110111111001100-12101--100001110000100-101100320230-02122010000000001101002000110111-2101---------------1110010-101011101001121110000--111000110121010001001000000001--10001113100001002010111110--1221000110011110001100110000000010010000020-0000

Chelus_fimbriatus 1--0101111001100-12101--100001111000000-101000320230-02122010000000001101002000110111-2101---------------1110210-101001100101121110000--111000110121111001020100100001--10111113100001012010111110--1221000110011110001100110000000000010000020-0000

Yaminuechelys_maior 0?1?????1100??0?-?2??????1000?1????0?0?????????????0-???22?????000000?1?100??00????11-?1?1---------------11100110100011?01001121110110--111000110111010001001000000001--100?11131000010120101????0--1?2??0??1????01???1???????000000002?0000020-0???

Araripemys_barretoi 1--1??011101110?-1211000100001110000?00-?11???3212?0-0212201010000???0101002??0??0111-01?????????????????11102112101011001101021110110--1111011101201100011---00000101--100?1110000001012??0111110--1221?0011?????????110011001100010031100002000000

Erymnochelys_madagascariensis 1--1110111011100-?01000010000111000000202111-0321230-121220100000110001?1002000110?11-?100---------------1100010-101011101001021110000--111000110011010001001100100001--1000111000000100201011??10--12???0??100111100?110011??0000000001000002000000

Pelomedusa_subrufa 1--1110111011100-121000010000111000000202111-0321230-(0 1)2122010000001000101002000110111-0100---------------1100010-101011101001021110000--111000110111010001001000000001--10001110000001002010111110--122100011001111000110011000000000001000002000000

Podocnemis_expansa 1--1110111011100-10100001000011100001021211100321230-12122010010011000101002000110111-2100---------------1100010-101011101001021110000--111000110111010001001000100001--10001110000001002010111110--122110011001111000110011000000000001000002000000

Dorsetochelys_delairi 0010001111001?0???01000000000?110?00000-10100?220211?011221000010000-00?1012?10?10?11-01?1---------------11?????????????????????????????????????????????????????????????????????????????????????????????????????????????????????000?????0??010000???

Pleurosternon_bullockii 0110??1111011100??01000000000?110000??0-1?10?0220211?01112?00101?0?0?0??1012000?10?????111---------------11?00111?00011?00001021011000--1??000??01010000010010000000000-00??0--------??000??????????????????0???1--00?????????000000000?0?0010000000

Glyptops_plicatulus 01100?11110?11?0??0100?0??00??110?01?01-1?1??0220211?01112100101000?-?0?1022000010011-21?1---------------11000111100011?00002121011000--1110001101010000000010000000000-?0000--------??0001010???0--112000010???1--00?1???????000000001?0?0010000000

Neurankylus_eximius ???0??11?10?11?00?1??????000?01???????????????220?1100212210?0??000000101?22000010011-?11????????????????11?00111100011?00002121111000--111000?10?01??00000010000000000-????????????????101?????????????????0????--???????????000000000?0?0010000000

Trinitichelys_hiatti 0010002111011100??110000000000110000?00-101?002202110?21221000010000001?1022000010011-0111---------------1100011110?01??0?0??12111?000--111000?10?01??00000011000000?00-??000--------?????10????????????????0?????????????????00000000??0??010000000

Plesiobaena_antiqua 00100021110111000?110000000000110000000-1010002202110021221000010000001?1022000010011-?111---------------11000111100011?0000?121110000--111000?10?01?00000021?000000100-000011????????0?101010??00--????????0???1--0??110011??0000000000000010000000

Boremys_pulchra 00100021110111000?110000000000110000????101?0022021100212210001?00000010102200001001???111---------------11000111100011?00002221110000--111000?10?01??0000021?000000?00-01000--------???1010112100--?2??????0????--???????????0000000000000010000000

Baena_arenosa ???00021110011000?010000000000110000001-1010002202110021221000010000-01?1022000010?11-?110???????????????11000111101011?00001221110000--111000?10111?000000211000000100-?10?0--------0001010112200--1220000101001--0011???????00000000??0??010000000

Chisternon_undatum 0010002111001100??110000000000110000001-1010002202110021221000110000001?1022000010011-0111---------------110001??100011?0000?2211-?000--111000?10?01?00000021100000010???0001112?0000??0101011??00-???2?000101001--0011???????000000000?0?0010000000

Portlandemys_macdowelli 00110001110?10010?1??????00000110000001-10100022021100112210000110???0101012000010011-0100???????????????110????????????????????????????????????????????????????????????????????????????????????????????????????????????????????0??????????113000???

Plesiochelys_etalloni 00110001110(0 1)10010?1100000000?0110000001-1010002202110011221010010010001010120000100????100???????????????1100010?100011?00002221010(0 1)(0 1)0--111000?10?210000(0 1)00010000000(0 1)00-????????????????0010????????1????0010???1--0??110011??000000001?0?(0 1)113000000

Solnhofia_parsonsi 00110001110(0 1)10000?110000?00000110000-10-1110-0220211001122100000101000101022000010011-0100???????????????1100010-??0011?0000312101?110--???001?0002000000??????000?00???110?0--------?????1?10??????1?2??0010?0??--???110011000000000?11001113000000

Thalassemys_moseri 00100011110011010?110000000000110000001-1110002202110011221010-?100000111022000010011-0100???????????????11?001??10001??0?0???2?0???10--???????1?02????00??????0?0?0????11??0--------0?01???????????????????????????????????????0000001?0?0113000000

Toxochelys_latiremys 1011000111011?0001110000000010110000000-10100022021100?122100001101000101022001010011-01??---------------1100010-200011000102121110110--11100110022010100???1??0???010??11?1111200??0?1??01011??00--1???????00???--0??111011??(0 1)0000000(1 2)111(0 1)113000000

Caretta_caretta 1--100011(0 1)00120001010000000010110100-10-1011002201111011221020-1101000101022101010011-0?00---------------1100010-200010000102121110110--111001100220?010001---10?00000??11111112000111102011111110--122111-100111--000112011001000000021111113000000

Chelonia_mydas 1--1?0011100120001010000000010110101-1201011002201110011221020-1101000101022101010011-0100---------------1100010-200011000102121110110--111001100220?010001---10?0?000??11111112000111102011111110--122111-100111--0001120110?100000002111?113000000

Mesodermochelys_undulatus ??????????????????????????????????????????????????????????????????10???????????????????????????????????????01010-200011000101?2???-110--111001?00?20?011----------------11101112000000102010111110--122111-101111--0001?20????100000?021100???000000

Dermochelys_coriacea 1--1010110000-00-1000000000010110000001-101000220110-021221020-0001000101022100010011-0101---------------110201-?20-0-300-1---2----11-??111001?0??20?011----------------111111120001101020-0111110--122111-101111--000112011??1-000--?01110113000000

Macroclemys_schmidti 1??1??011001110?0121000000000?110?01000-10100?2202410011221000?110100010102??00?10010101?0???????????????????????????????????????????????????????????????????????????????????????????????????????????????????????????????????????????0?????1?3000?0?

Macroclemys_temminckii 1--100011001110001210000000000110001000-101000220221001122100?1110100010102200001001010100???????????????1100010-120001000001111110110--1110011002201000001-1-00?001100-11111112001011102011112200--1221000100001--0011100111?1000000020111113000000

Protochelydra_zangerli 1--10?0110?11100???100000000?0110000????101???220?21???12210001???10?0??10???0?010??????0????????????????11???1???????????0???????0100--111001?0??20?0000?1-1-00?0?110???????????????????????????????2??????0???1--0??????????00000000?????113000?0?

Chelydra_serpentina 1--10001100111000?210000000000110001000-101000220221001122100011101000101022000010010??100???????????????1100010-120001000003121110110--111001?002201000001-1-0010?1100-11111112001011102011111200--1221000100011--00?110011??1000000000111113000000

Platysternon_megacephalum 1--100011001110001010010000000110001000-10100022022100112210001110100010102210001001010101---------------1100010-100001100002121110100--1110001002202100001-1-000000000-11011112101011102011111200--1220000100011--0011100110?0000000020011113000000

Mongolemys_elegans 1--(0 1)000111011100??21000000000011000000001010002202110011221000010010??00102200001001??0101---------------1100010-10?0?10000??121110000--111000?100210100001-1-00?000000-????1112101011102?111?1100--12??????0???1--0??1100????0000000001010111000000

Gopherus_polyphemus 1--100011101110011210000100000110000002010101022022100112210001100100010102200001001010101---------------1100010-101001000012121110000--1110000100210100001-1-000010001011111112101011102011111110--1220001101011--001100011100000000001010113000000

Eurotestudo_hermanni 1--10001110111001?210000100000110000002010101022022100112210001100100011102200001001010101---------------1100010-101001000012121110000--1110000100200100001-1-000010001011111112101011102011111110--1221001101011--001100011100000000001010113000000

Chelonoidis_gringorum ????????????????????????????????????????????????????????????????????????????????????????????????????????????0010-??1001000012001110000--1110000?0?2??100001-1-0000100010???????????????????????????????1?0?101????????????????0000000001010???000000

Chelonoidis_chilensis 1--100011101110011210000100000110000002010101022022100112210001100100010102200001001010101---------------1100010-101001000012021100000--111000000021?100001-1-000010001011011112101011102011111110--122(0 1)001101011--00010001110000000000?0?0113000000

Stylemys_nebraskensis 1--1??0111011?0???21000010000?11?????0????1????202?1?0?122?0???1?0??????10????0????????1?1???????????????11?0010-?0?0?1000002121110000--111000110122?100001-1-0000100010????????????????????????????1?2100111100?--??110001100000000000?0?0113000000

Chrysemys_picta 1--100011101110011210000100000110000002010100022021100112200001100100011102200001001010101---------------1100010-101011000001121110000--1110001101210100001-1-000010001011111112111011102011111110--1221001101011--001110011000000000001010113000000

Trachemys_scripta 1--100011100110011210000100000110000002110100022021100112210001100100011102200001001010101---------------1100010-101011000011121110000--1110001101210100001-1-000000001011?---12111011102011--??-0----21001101011--0011-----000000000001010113000000

Emys_orbicularis 1--100011001110011210000?00000110000000-10100022021100112200001100100011102200001001010101---------------1100010-101011000001121110101001010001001201000001-1-0010000???1111111210101110201111111???1221001101?11--00?110011000000000001010113000000

Geoclemys_hamiltonii 1--100011001110011211000100000110000000-10101022021100112210001110100011102200001001010101---------------1100110-101011100002121110000--1110000101211100001-1-001010001011111112101011102011111110--1221001101011--001110011000000000001010113000000

Echmatemys_wyomingensis ????????????????????????????????????????????????????????????????????????????????????????????????????????????0010-101011?0000?121110000--1110001100211100001-1-0010000010????????????????201?????????12??????0???1--?0?????????000000000?0?0113???0?0

Emarginachelys_cretacea 1--1000111?111000??100001000?0110000????1010?1220211??1122100011??10?01?102200?010???????1---------------11?011??1{1 2}?0?1?000???21110100--111000?0?120?000001-1-00????00??11??111???????????1?????????1?2?????0???1--01?110011??00000000001?0113000000

Baptemys_wyomingensis 1--10001110111000?210000100000110000002?1010012202110011221000(0 1)?001000101022?000-2-1??0101---------------1100110-100011?0000?121110000--111000110121?000001-1-001100000-11?1111?????????2?1111??1???122?????01??1--1??110011??000000000?0?0113000000

Dermatemys_mawii 1--100011(0 1)0111000?21000010000011000000201010012202110011221000110010001010220000-2-1010101---------------1100010-100011101001121110000--111000?101200100001-1-10010000001111110-00101110201111??10--1221001101101--00?110011000000000001010113000000

Xenochelys_formosa 1--10?01100?1100112??????00????10?01000-1?10??2????1?01122?020-????????0?????00?110????1?1---------------11?0110-1?1012101001121110000--1110001002201100001-1-00-1-20011??????????????????????????????????????????????????????000000001?0?0113??0000

Staurotypus_triporcatus 1--100011001110011211010100000110001?00-10100122021110112210001000100010102200001101010101---------------1100210-11001210(0 1)001121110001011110001002201000001-1-00-1-20011111111110010111021-1111?10--1221001100101--110110011??0000000001110113000000

Sternotherus_odoratus 1--100011001110011210010100000110000000-10100122021110112210001100100010102200001101010101---------------1100110-11001211100112111000101---?1-1002201000001-1-11-1-20011111111110010111021-1111110--1221001101101--110110011000000000001110113000000

Kinosternon_flavescens 1--100011001110011210010100000110000000-10100122021110112210001000100010102200001101??0101---------------1100110-11001201100112111000111---?1-1002201000001-1-11-1-20011111111110???111021-1111110--1221001101111--1101100110?0000000001010113000000

Basilemys_variolosa 1--1??0111?1??00???10000100?00110?00??????1????20?21???122?0????????????10???????????????????????????????11000112101111?0(0 1)001121111000--111000100020?000100(0 2)11001000001011?11?0-0????????????????????2??????01011--00?1000??000?0000002?010111000000

Yehguia_tatsuensis ????????????????????????????????????????????????????????????????????????????????????????????????????????????00112????11???001?2???1000--111000??0?2??000100010000000000-????????????????????????????1???????0????????11???????00000010?10????????0?0

Adocus_beatus 1--1000111011100112100001001001100000021101001220211101122100011101010101022000010011-1100???????????????11000112101111?0(0 1)00?121111000--1110001000200000100010001010000-1111100-001011102?111???????1220001101011--011110011??000000000?010111000000

Hoplochelys_crassa ????????????????????????????????????????????????????????????????????????????????????????????????????????????0210-11?0?1?000??121110000--111000000020?000001-1-00-1-1000-????????????????201?????????122??????????--???????????00000000??0????????000

Apalone_spinifera 1--101011101110011211000100100111210101-111101220221101122100111101010101022000010011-1101---------------11020112100013001-00------110--11110-100-20-0-1----1-----------1101120-00101110201-111110--1221101100111--00011011101(0 1)0000--001-11113000-00

Plastomenus_aff._thomassii 1--10?011101110?11211000100?0?11----100-11110?220221101122100111101010101022?00??0010111?1---------------11020112100013001-00------100--?????01-0-2--??1----------------????????????????20??1?????????????????????????????????10000--00?-?1113000-00

Pelodiscus_sinensis 1--10101110111001121100010010011?2??100-111101220221101122100101101010101022000010011-11?1---------------11?20112100013001-00------110--1111001-0-2--001----------------110?120-00?0111020--11??10--?????0??????????????????0110000--001-11113000-00

Lissemys_punctata 1--101011101110011211000100100111210100-11110122022110112210011010101010102200001001001101---------------11020112100013001-00------110--11110-101-20-0-1----1-----------1101120-00101110201-11--10--1221101100111--0101101110110000--001-11113000-00

Shachemys_laosiana 1--1??011101??0?11(1 2)??????00???11??????????????2????????12210001??0????1110???00?1?0????1?????????????????11?0010-??10-1?(0 1)20010210-0100--111000?000201000000010001000000-????????????????????1?????????????????????????????????000000000?0??113???0?0

Anosteira_ornata 1--1??0111?1??001??10000100??011?200????1111??220221???1221001(0 1)1?010?01?1022?0?010??????01---------------11?101121000120010001211?-100--1110001002201001----1-----------?????????????????????????????????????????--???110211??000000001?-?1113000000

Carettochelys_insculpta 1--101011101110011210010100100111200000-111101220221101122100111101010101022000010011-1101---------------11010112100012001001-2----100--1110001002201001----1-----------1101110-001011102011111110--122001-100111--0101102110000000-0001-11113000-00

Xinjiangchelys_wusu 0110?0111100??0?01210000000000110?000-??1?1?0?22021100?112000001101000001022?00?100?1-01?0011?????0??00??1100010-??001110(0 1)002121111100--1?10001000200000000010000000100-0(0 1)????-------1?0???????????????0?001?????--????100110000000010100?0111000000

Annemys_levensis 00?1??01110?1100012100000000001100000-0-1?1000220211001112000001?01000001022?00?100?1-0100011?????0?0000?1100010-100011101002121101100--1110001000200000100010000000100-????????????????0?00????????112000010???1--?0?????????000000102?0?0111000000

Annemys_latiens 01?0??11110?110001210?0??0????11?????????????02???1100111200?0???0?0???0102??00?100??-?1?????????????????11?0010-100011?0(0 1)0021211(0 1)1100--1110001000200000100010000000100-???????????????????????????????000010????--???????????000000102?0?0111???0?0

Xinjiangchelys_radiplicatoides 01?0??11110?110001{1 2}?0????0????11??0??????????0220?1100111200?????0?00000102??000100?1-01?????????????????11?0010-1000?1?0000212111??00--1?10001000200000100010000000100-????????????????0???????????11200001?????--???????????000000101?0?0111???000

Xinjiangchelys_junggarensis ????????????????????????????????????????????????????????????????????????????????????????????????????????????001??100011100002121111100--1110001000200000100010000000100-?11111??????011?000011??00--112?????0???1--0??1???????000000101?000??????0?0

Hangaiemys_hoburensis 001100011100110001110000000000110000000-10100022?211001112{0 1}00001001000001022010010010101?0???????????????1100010-100011?0000?121110100--111001100020?000001-1?000000(0 1)00-?1111112000001102?10?1??????1220?0010???1?????????????000000100?110111000000

Changmachelys_bohlini ???1??011100??0??111000000000011????000-??????22021??0?1??100?01?0100?00102??1001001??0??????????????????1100010-?00011?0000?1211??1?0--1?100??00?20000??????????????????1111?1?0??0?1?0????1????0--1?2??0?10???1--????1001100?000001?101?0111???000

Judithemys_sukhanovi ???1??01110?1100??110000?0000011???0?0??101?0?220211?0?1121000011010000?1022010010011-01?????????????????1100010?100011?00002121110100--111001?000200000001-1?000000(0 1)00-?1111112000001102?10111210--122000010???1--00?1100110?0000000000110111000000

Dracochelys_bicuspis ????0???110?110001??0000?000??110000001-10100?220{1 2}1100111210000110?0?01?1022?0001001??01?1---------------110001??100011?000020?111?110--???001?000200000????????????????0111110-000001100010?????0--1?20?0010???????????00????010111012?101111000000

Ordosemys_leios 0??????1????????0?{1 2}????0????????????000-???????2??111???1???0?0????????0?????10?100???01????????????????????0010?100011100002121010110--???00??0002000000??????0?0?0(0 1)00-011?111210000?100??01112?0--1?20?001000?1??00?1???????100000101010(0 1)??????0?0

Sinemys_brevispinus ????????????1???01?????0?000??1????0??????????2212?100?11?0?????00?0?0001022?10?1001??0?????????????????????0011??00?11?0?00?0211?0110--11100010002000000???1??0?0001???11??1?0-000001?00????112????122?10010?????????????????1?100101201?1111000000

Sinemys_lens 0010??111101110001210?00?0000?11??00?-0-??1???22?2?100?1?200000?0010??001022?1001001??01?1---------------110001??1000111000020211101(0 1)0--11100010002000000???1??0?000?0??????????????????0???1???????????????0??????????100110001100101201?1111000000

Sinemys_gamera 0?10??111101110?01210?00000?0011????0-0-??????2212210011120000?10010?0001022?10010010101?1---------------11?0011?????1?10000??2?11?1???????????????00??0???????????0???????????????????????????????????00001???????????????????1?0??01?????111000000

Liaochelys_jianchangensis 0011?0011101??0?01210???0000??110?0????????????????10???????0?????100??010??????100???????????????????????100010???0?11?01002121010110--???001?00?20?000????????????????011?1????????1?0????1112?0--??????????????????????11??100110(0 1)1101?(0 1)????0?000

new_taxon 1--1?0111101??0?011101--000000110?00?0?-101??02??2?100?111100001?0100?0010???10?100???01?0???????????????1100010-?00111?00002121110100--111001?000200000???????0000000??11111?12?????0?0?01?111200--1220?0?1?0?11--0???1?011000000001110100111?00?00

Basilochelys_macrobios ????????????1100012?????????????????????101???2????110111200?0??10????0010???10?100???01??????????????????1?0011???0111?0?00?121111000--111000?00{0 1}2??000????1??01???0????11??--------1?00??1????????????????01011--?011???????0?0000100?0?0111?0?000

Xiaochelys_ningchengensis_ ???1??01110???0?01{1 2}10?0?0000??110?0????????????????1??????????????100??010??????100???????????????????????1?0010-??0011?00002121?1010???????????0?200??0???????????0?0???1??1?12?????1?0????111200--???????????????????????????000001110??0?????????

Kallokibotion_bajazidi 00?0??011?001??00?0100000?000?100?00?0??111??02202?10011121??0??00?00?011?1?0?0?10011-01?0???????????????1100010-00001?000002??100??00--1?1000??0?1??00000011000?000000-?0000--------0?0?????0--00--?210000001001--00?????????000000?0000??010000000

Mongolochelys_efremovi 0(0 1)10001110001000010100000110001100000021101000120211000112000001000000011012?10?10011-010000001--011-00001100010-000001?000021210-0110--1010011000000000000110000000000-00001111100000000000111200--111000010?0?1--00?1100?1001(0 1)000000300?0010111000

Sichuanchelys_palatodentata 011000101000?{0 1}0?01010000000000110000001-10100?12021100?012000001000000001012?10?10011-011000?00???0??00??1100010-?0001{0 1}?0000??2101?110--1?1001100{0 1}0000000001100000000????00??????????0?0????10--00--1?11?0010????--??11?0?????100000?03?0?0000111000

Sichuanchelys_chowi ????????????????????????????????????????????????????????????????????????????????????????????????????????????0010-?00011?0?0031210?0?00--???001??0?0??00000011?000000000-??????????????????????????????????????????????????????0?00?0?03?0?0??????0?0

Helochelydra_nopcsai 0?10??1111001{0 1}00??010010000000110?00010-1?1?002202110011?210000?0001000?100??00?10001-01?0???????????????1100011-????1????00?????????????1100??????????0????????????????????????????????????????????????????????????????????????????????0???1????111

Naomichelys_speciosa 0110??111100??0001010000000000110?000??-??1???22021100?1??1??0??00000000100??00?10001-0??0???????????????1100011-?00011?0000?121010010--11100010010000000???1?000000000-00001112?00100000000100000--1?1000010?011--000110001000000000020000?1?000111

Spoochelys_ormondea ?????????0001000-1010000?0000?1????00?????????{0 1}2?{1 2}1?-00?100??0??000???001?12000?10011-00?0011??0-000??0?0?100?10-?0??????????1{0 1}????1???????????0????????????????????????000011120000000??????12?0???1?100000??????????????????????????0?000000000?0?

Siamochelys_peninsularis ????????????????????????????????????????????????????????????????????????????????????????????????????????????0011??000111000021211?0100--1?1000??01201000100010000000?00-??????????????????????????????????????????????????????00000010(1 2)?0?0??????000

Manchurochelys_manchoukuoensis ???1?00?1101??0?01210?0??0000?110??00-??101?0?2????100?11210?00?10100?0010???10?100?1-01?1---------------1100010???0?11?000021211101???-?????????????????????????????????11?1?0?0????1?0????1???00--1?20?0?1??????????????11??00100011001?0111?0?000

;

END;

BEGIN TAXA;

TITLE Taxa5;

DIMENSIONS NTAX=100;

TAXLABELS

Sphenodon_punctatus Simosaurus_gaillardoti Anthodon_serrarius Owenetta_kitchingorum Odontochelys_semitestacea Proganochelys_quenstedti Proterochersis_robusta Kayentachelys_aprix Condorchelys_antiqua Indochelys_spatulata_2 Heckerochelys_romani Eileanchelys_waldmani Chengyunchelys Siamochelys_peninsularis Dorsetochelys_delairi Notoemys_laticentralis Platychelys_oberndorferi Caribemys_oxfordiensis Phrynops_geoffroanus Prochelidella_cerrobarcinae Yaminuechelys_maior Chelus_fimbriatus Chelodina_colliei Chelodina_longicollis Elseya_dentata Myuchelys_latisternum Araripemys_barretoi Pelomedusa_subrufa Erymnochelys_madagascariensis Podocnemis_expansa Xinjiangchelys_latimarginalis Hangaiemys_hoburensis Judithemys_sukhanovi Solnhofia_parsonsi Thalassemys_moseri Santanachelys_gaffneyi Dracochelys_bicuspis Sinemys_lens Ordosemys_leios Toxochelys_latiremys Caretta_caretta Chelonia_mydas Mesodermochelys_undulatus Dermochelys_coriacea Platysternon_megacephalum Protochelydra_zangerli Macroclemys_schmidti Macroclemys_temminckii Chelydra_serpentina Emys_orbicularis_2 Geoclemys_hamiltonii Echmatemys_wyomingensis Mongolemys_elegans Stylemys_nebraskensis Gopherus_polyphemus Eurotestudo_hermanni_2 Chelonoidis_gringorum Chelonoidis_chilensis Chrysemys_picta Trachemys_scripta Emarginachelys_cretacea Baptemys_wyomingensis Dermatemys_mawii Hoplochelys_crassa Xenochelys_formosa Staurotypus_triporcatus Sternotherus_odoratus Kinosternon_flavescens Shachemys_laosiana_2 Adocus_beatus Basilemys_variolosa Yehguia_tatsuensis Anosteira_ornata Carettochelys_insculpta Apalone_spinifera Plastomenus_aff._thomassii Pelodiscus_sinensis Lissemys_punctata Portlandemys_macdowelli Plesiochelys_etalloni Neurankylus_eximius Trinitichelys_hiatti Plesiobaena_antiqua Boremys_pulchra Baena_arenosa Chisternon_undatum Pleurosternon_bullockii Glyptops_plicatulus Dinochelys_whitei Chubutemys_copelloi Mongolochelys_efremovi Patagoniaemys_gasparinae Otwayemys_cunicularius Kallokibotion_bajazidi Niolamia_argentina_Crossochelys Ninjemys_oweni Warkalania_carinaminor Meiolania_platyceps Palaeochersis_talampayensis Australochelys_africanus

;

END;

BEGIN TAXA;

TITLE Taxa;

DIMENSIONS NTAX=101;

TAXLABELS

Sphenodon_punctatus Simosaurus_gaillardoti Anthodon_serrarius Owenetta_kitchingorum Odontochelys_semitestacea Proganochelys_quenstedti Proterochersis_robusta Kayentachelys_aprix Condorchelys_antiqua Indochelys_spatulata Heckerochelys_romani Eileanchelys_waldmani Chengyunchelys Siamochelys_peninsularis Dorsetochelys_delairi Notoemys_laticentralis Platychelys_oberndorferi Caribemys_oxfordiensis Phrynops_geoffroanus Prochelidella_cerrobarcinae Yaminuechelys_maior Chelus_fimbriatus Chelodina_colliei Chelodina_longicollis Elseya_dentata Myuchelys_latisternum Araripemys_barretoi Pelomedusa_subrufa Erymnochelys_madagascariensis Podocnemis_expansa Xinjiangchelys_latimarginalis Hangaiemys_hoburensis Solnhofia_parsonsi Thalassemys_moseri Santanachelys_gaffneyi Judithemys_sukhanovi Dracochelys_bicuspis Sinemys_lens Ordosemys_leios Toxochelys_latiremys Caretta_caretta Chelonia_mydas Mesodermochelys_undulatus Dermochelys_coriacea Platysternon_megacephalum Protochelydra_zangerli Chelydra_serpentina Macroclemys_schmidti Macroclemys_temminckii Emys_orbicularis Geoclemys_hamiltonii Echmatemys_wyomingensis Mongolemys_elegans Stylemys_nebraskensis Eurotestudo_hermanni Gopherus_polyphemus Chelonoidis_gringorum Chelonoidis_chilensis Chrysemys_picta Trachemys_scripta Emarginachelys_cretacea Baptemys_wyomingensis Dermatemys_mawii Hoplochelys_crassa Xenochelys_formosa Staurotypus_triporcatus Sternotherus_odoratus Kinosternon_flavescens Shachemys_laosiana Adocus_beatus Basilemys_variolosa Yehguia_tatsuensis Plastomenus_aff._thomassii Lissemys_punctata Apalone_spinifera Pelodiscus_sinensis Anosteira_ornata Carettochelys_insculpta Portlandemys_macdowelli Plesiochelys_etalloni Neurankylus_eximius Trinitichelys_hiatti Plesiobaena_antiqua Baena_arenosa Boremys_pulchra Chisternon_undatum Pleurosternon_bullockii Glyptops_plicatulus Dinochelys_whitei Chubutemys_copelloi Niolamia_argentina Ninjemys_oweni Warkalania_carinaminor Meiolania_platyceps Patagoniaemys_gasparinae Otwayemys_cunicularius Kallokibotion_bajazidi Mongolochelys_efremovi Peligrochelys Palaeochersis_talampayensis Australochelys_africanus

;

END;

BEGIN AUTHORS;

AUTHOR NAME = Zhou CODE = 0;

END;

BEGIN TREES;

Title 'Trees from "trees.nex"';

ID 01624961b33c1;

LINK Taxa = Taxa;

TRANSLATE

[0] 1 Sphenodon_punctatus,

[1] 2 Simosaurus_gaillardoti,

[2] 3 Anthodon_serrarius,

[3] 4 Owenetta_kitchingorum,

[4] 5 Odontochelys_semitestacea,

[5] 6 Proganochelys_quenstedti,

[6] 7 Proterochersis_robusta,

[7] 8 Kayentachelys_aprix,

[8] 9 Condorchelys_antiqua,

[9] 10 Indochelys_spatulata,

[10] 11 Heckerochelys_romani,

[11] 12 Eileanchelys_waldmani,

[12] 13 Chengyunchelys,

[13] 14 Siamochelys_peninsularis,

[14] 15 Dorsetochelys_delairi,

[15] 16 Notoemys_laticentralis,

[16] 17 Platychelys_oberndorferi,

[17] 18 Caribemys_oxfordiensis,

[18] 19 Phrynops_geoffroanus,

[19] 20 Prochelidella_cerrobarcinae,

[20] 21 Yaminuechelys_maior,

[21] 22 Chelus_fimbriatus,

[22] 23 Chelodina_colliei,

[23] 24 Chelodina_longicollis,

[24] 25 Elseya_dentata,

[25] 26 Myuchelys_latisternum,

[26] 27 Araripemys_barretoi,

[27] 28 Pelomedusa_subrufa,

[28] 29 Erymnochelys_madagascariensis,

[29] 30 Podocnemis_expansa,

[30] 31 Xinjiangchelys_latimarginalis,

[31] 32 Hangaiemys_hoburensis,

[32] 33 Solnhofia_parsonsi,

[33] 34 Thalassemys_moseri,

[34] 35 Santanachelys_gaffneyi,

[35] 36 Judithemys_sukhanovi,

[36] 37 Dracochelys_bicuspis,

[37] 38 Sinemys_lens,

[38] 39 Ordosemys_leios,

[39] 40 Toxochelys_latiremys,

[40] 41 Caretta_caretta,

[41] 42 Chelonia_mydas,

[42] 43 Mesodermochelys_undulatus,

[43] 44 Dermochelys_coriacea,

[44] 45 Platysternon_megacephalum,

[45] 46 Protochelydra_zangerli,

[46] 47 Chelydra_serpentina,

[47] 48 Macroclemys_schmidti,

[48] 49 Macroclemys_temminckii,

[49] 50 Emys_orbicularis,

[50] 51 Geoclemys_hamiltonii,

[51] 52 Echmatemys_wyomingensis,

[52] 53 Mongolemys_elegans,

[53] 54 Stylemys_nebraskensis,

[54] 55 Eurotestudo_hermanni,

[55] 56 Gopherus_polyphemus,

[56] 57 Chelonoidis_gringorum,

[57] 58 Chelonoidis_chilensis,

[58] 59 Chrysemys_picta,

[59] 60 Trachemys_scripta,

[60] 61 Emarginachelys_cretacea,

[61] 62 Baptemys_wyomingensis,

[62] 63 Dermatemys_mawii,

[63] 64 Hoplochelys_crassa,

[64] 65 Xenochelys_formosa,

[65] 66 Staurotypus_triporcatus,

[66] 67 Sternotherus_odoratus,

[67] 68 Kinosternon_flavescens,

[68] 69 Shachemys_laosiana,

[69] 70 Adocus_beatus,

[70] 71 Basilemys_variolosa,

[71] 72 Yehguia_tatsuensis,

[72] 73 Plastomenus_aff._thomassii,

[73] 74 Lissemys_punctata,

[74] 75 Apalone_spinifera,

[75] 76 Pelodiscus_sinensis,

[76] 77 Anosteira_ornata,

[77] 78 Carettochelys_insculpta,

[78] 79 Portlandemys_macdowelli,

[79] 80 Plesiochelys_etalloni,

[80] 81 Neurankylus_eximius,

[81] 82 Trinitichelys_hiatti,

[82] 83 Plesiobaena_antiqua,

[83] 84 Baena_arenosa,

[84] 85 Boremys_pulchra,

[85] 86 Chisternon_undatum,

[86] 87 Pleurosternon_bullockii,

[87] 88 Glyptops_plicatulus,

[88] 89 Dinochelys_whitei,

[89] 90 Chubutemys_copelloi,

[90] 91 Niolamia_argentina,

[91] 92 Ninjemys_oweni,

[92] 93 Warkalania_carinaminor,

[93] 94 Meiolania_platyceps,

[94] 95 Patagoniaemys_gasparinae,

[95] 96 Otwayemys_cunicularius,

[96] 97 Kallokibotion_bajazidi,

[97] 98 Mongolochelys_efremovi,

[98] 99 Peligrochelys,

[99] 100 Palaeochersis_talampayensis,

[100] 101 Australochelys_africanus;

TREE Strict_Consensus_tree = (1,(2,(3,(4,(5,(6,((100,101),(7,(8,9,10,(11,(12,((90,((98,99),((95,(96,97)),(91,(92,(93,94)))))),(13,(14,((87,(88,89)),(15,((81,(82,(83,(84,85,86)))),((79,80),(((16,(17,18)),((27,(28,(29,30))),((25,26),(19,((20,21),(22,(23,24))))))),(31,(32,36,(33,(34,35)),(37,(38,39)),((40,((41,42),(43,44))),(45,((46,(47,48,49)),((50,(51,52,((59,60),(53,(54,(55,56,(57,58))))))),((61,((62,63),(64,(65,66,(67,68))))),((69,(70,(71,72))),(77,78,(73,74,75,76)))))))))))))))))))))))))))));

TREE tnt_1 = (1,(2,(3,(4,(5,(6,((7,((8,(9,10)),(11,(12,((13,(14,((15,(((((16,(17,18)),(((19,((20,21),(22,(23,24)))),(25,26)),(27,(28,(29,30))))),(31,(32,((33,(34,35)),((36,(37,(38,39))),((40,((41,42),(43,44))),(45,((46,(47,(48,49))),((50,(51,(52,((53,(54,(55,(56,(57,58))))),(59,60))))),((61,((62,63),(64,(65,(66,(67,68)))))),((69,(70,(71,72))),((73,(74,(75,76))),(77,78))))))))))))),(79,80)),(81,(82,(83,(84,(85,86))))))),(87,(88,89))))),(90,(((91,(92,(93,94))),(95,(96,97))),(98,99)))))))),(100,101))))))));

TREE tnt_2 = (1,(2,(3,(4,(5,(6,((7,((8,(9,10)),(11,(12,((13,(14,((15,(((((16,(17,18)),(((19,((20,21),(22,(23,24)))),(25,26)),(27,(28,(29,30))))),(31,(32,((33,(34,35)),((36,(37,(38,39))),((40,((41,42),(43,44))),(45,((46,(47,(48,49))),((50,(51,(52,((53,(54,(55,(56,(57,58))))),(59,60))))),((61,((62,63),(64,(65,(66,(67,68)))))),((69,(70,(71,72))),((73,(74,(75,76))),(77,78))))))))))))),(79,80)),(81,(82,(83,(85,(84,86))))))),(87,(88,89))))),(90,(((91,(92,(93,94))),(95,(96,97))),(98,99)))))))),(100,101))))))));

TREE tnt_3 = (1,(2,(3,(4,(5,(6,((7,((8,(9,10)),(11,(12,((13,(14,((15,(((((16,(17,18)),(((19,((20,21),(22,(23,24)))),(25,26)),(27,(28,(29,30))))),(31,((33,(34,35)),((32,(36,(37,(38,39)))),((40,((41,42),(43,44))),(45,((46,(47,(48,49))),((50,(51,(52,((53,(54,(55,(56,(57,58))))),(59,60))))),((61,((62,63),(64,(65,(66,(67,68)))))),((69,(70,(71,72))),((73,(74,(75,76))),(77,78)))))))))))),(79,80)),(81,(82,(83,(84,(85,86))))))),(87,(88,89))))),(90,(((91,(92,(93,94))),(95,(96,97))),(98,99)))))))),(100,101))))))));

TREE tnt_4 = (1,(2,(3,(4,(5,(6,((7,((8,(9,10)),(11,(12,((13,(14,((15,(((((16,(17,18)),(((19,((20,21),(22,(23,24)))),(25,26)),(27,(28,(29,30))))),(31,(32,((33,(34,35)),((36,(37,(38,39))),((40,((41,42),(43,44))),(45,((46,(47,(48,49))),((50,(51,(52,((53,(54,(55,(56,(57,58))))),(59,60))))),((61,((62,63),(64,((65,66),(67,68))))),((69,(70,(71,72))),((73,(74,(75,76))),(77,78))))))))))))),(79,80)),(81,(82,(83,(84,(85,86))))))),(87,(88,89))))),(90,(((91,(92,(93,94))),(95,(96,97))),(98,99)))))))),(100,101))))))));

TREE tnt_5 = (1,(2,(3,(4,(5,(6,((7,((8,(9,10)),(11,(12,((13,(14,((15,(((((16,(17,18)),(((19,((20,21),(22,(23,24)))),(25,26)),(27,(28,(29,30))))),(31,(32,((33,(34,35)),((36,(37,(38,39))),((40,((41,42),(43,44))),(45,((46,(47,(48,49))),((50,(51,(52,((53,(54,(55,(56,(57,58))))),(59,60))))),((61,((62,63),(64,(65,(66,(67,68)))))),((69,(70,(71,72))),(((75,76),(73,74)),(77,78))))))))))))),(79,80)),(81,(82,(83,(84,(85,86))))))),(87,(88,89))))),(90,(((91,(92,(93,94))),(95,(96,97))),(98,99)))))))),(100,101))))))));

TREE tnt_6 = (1,(2,(3,(4,(5,(6,((7,(8,((9,10),(11,(12,((13,(14,((15,(((((16,(17,18)),(((19,((20,21),(22,(23,24)))),(25,26)),(27,(28,(29,30))))),(31,(32,((33,(34,35)),((36,(37,(38,39))),((40,((41,42),(43,44))),(45,((46,(47,(48,49))),((50,(51,(52,((53,(54,(55,(56,(57,58))))),(59,60))))),((61,((62,63),(64,(65,(66,(67,68)))))),((69,(70,(71,72))),((73,(74,(75,76))),(77,78))))))))))))),(79,80)),(81,(82,(83,(84,(85,86))))))),(87,(88,89))))),(90,(((91,(92,(93,94))),(95,(96,97))),(98,99))))))))),(100,101))))))));

TREE tnt_7 = (1,(2,(3,(4,(5,(6,((7,(8,(10,(9,(11,(12,((13,(14,((15,(((((16,(17,18)),(((19,((20,21),(22,(23,24)))),(25,26)),(27,(28,(29,30))))),(31,(32,((33,(34,35)),((36,(37,(38,39))),((40,((41,42),(43,44))),(45,((46,(47,(48,49))),((50,(51,(52,((53,(54,(55,(56,(57,58))))),(59,60))))),((61,((62,63),(64,(65,(66,(67,68)))))),((69,(70,(71,72))),((73,(74,(75,76))),(77,78))))))))))))),(79,80)),(81,(82,(83,(84,(85,86))))))),(87,(88,89))))),(90,(((91,(92,(93,94))),(95,(96,97))),(98,99)))))))))),(100,101))))))));

TREE tnt_8 = (1,(2,(3,(4,(5,(6,((7,((8,(9,10)),(11,(12,((13,(14,((15,(((((16,(17,18)),(((19,((20,21),(22,(23,24)))),(25,26)),(27,(28,(29,30))))),(31,(32,((33,(34,35)),((36,(37,(38,39))),((40,((41,42),(43,44))),(45,((46,(47,(48,49))),((50,(51,(52,((53,(54,(55,(56,(57,58))))),(59,60))))),((61,((62,63),(64,(65,(66,(67,68)))))),((69,(70,(71,72))),(78,(77,(74,(73,(75,76)))))))))))))))),(79,80)),(81,(82,(83,(84,(85,86))))))),(87,(88,89))))),(90,(((91,(92,(93,94))),(95,(96,97))),(98,99)))))))),(100,101))))))));

TREE tnt_9 = (1,(2,(3,(4,(5,(6,((7,((8,10),(9,(11,(12,((13,(14,((15,(((((16,(17,18)),(((19,((20,21),(22,(23,24)))),(25,26)),(27,(28,(29,30))))),(31,(32,((33,(34,35)),((36,(37,(38,39))),((40,((41,42),(43,44))),(45,((46,(47,(48,49))),((50,(51,(52,((53,(54,(55,(56,(57,58))))),(59,60))))),((61,((62,63),(64,(65,(66,(67,68)))))),((69,(70,(71,72))),((73,(74,(75,76))),(77,78))))))))))))),(79,80)),(81,(82,(83,(84,(85,86))))))),(87,(88,89))))),(90,(((91,(92,(93,94))),(95,(96,97))),(98,99))))))))),(100,101))))))));

TREE tnt_10 = (1,(2,(3,(4,(5,(6,((7,(10,((8,9),(11,(12,((13,(14,((15,(((((16,(17,18)),(((19,((20,21),(22,(23,24)))),(25,26)),(27,(28,(29,30))))),(31,(32,((33,(34,35)),((36,(37,(38,39))),((40,((41,42),(43,44))),(45,((46,(47,(48,49))),((50,(51,(52,((53,(54,(55,(56,(57,58))))),(59,60))))),((61,((62,63),(64,(65,(66,(67,68)))))),((69,(70,(71,72))),((73,(74,(75,76))),(77,78))))))))))))),(79,80)),(81,(82,(83,(84,(85,86))))))),(87,(88,89))))),(90,(((91,(92,(93,94))),(95,(96,97))),(98,99))))))))),(100,101))))))));

TREE tnt_11 = (1,(2,(3,(4,(5,(6,((7,(10,(8,(9,(11,(12,((13,(14,((15,(((((16,(17,18)),(((19,((20,21),(22,(23,24)))),(25,26)),(27,(28,(29,30))))),(31,((33,(34,35)),(((32,36),(37,(38,39))),((40,((41,42),(43,44))),(45,((46,(48,(49,47))),((50,(((53,(54,(56,(55,(57,58))))),(59,60)),(51,52))),((61,((62,63),(64,(65,(66,(67,68)))))),((69,(70,(71,72))),((75,(76,(73,74))),(77,78)))))))))))),(79,80)),(81,(82,(83,(84,(85,86))))))),(87,(88,89))))),(90,(((91,(92,(93,94))),(95,(96,97))),(98,99)))))))))),(100,101))))))));

TREE tnt_12 = (1,(2,(3,(4,(5,(6,((7,(10,(8,(9,(11,(12,((13,(14,((15,(((((16,(17,18)),(((19,((20,21),(22,(23,24)))),(25,26)),(27,(28,(29,30))))),(31,((33,(34,35)),(((32,36),(37,(38,39))),((40,((41,42),(43,44))),(45,((46,(48,(49,47))),((50,(((53,(54,(56,(55,(57,58))))),(59,60)),(51,52))),((61,((62,63),(64,(65,(66,(67,68)))))),((69,(70,(71,72))),((75,(76,(73,74))),(77,78)))))))))))),(79,80)),(81,(82,(83,(85,(84,86))))))),(87,(88,89))))),(90,(((91,(92,(93,94))),(95,(96,97))),(98,99)))))))))),(100,101))))))));

TREE tnt_13 = (1,(2,(3,(4,(5,(6,((7,(10,(8,(9,(11,(12,((13,(14,((15,(((((16,(17,18)),(((19,((20,21),(22,(23,24)))),(25,26)),(27,(28,(29,30))))),(31,(32,((33,(34,35)),((36,(37,(38,39))),((40,((41,42),(43,44))),(45,((46,(48,(49,47))),((50,(((53,(54,(56,(55,(57,58))))),(59,60)),(51,52))),((61,((62,63),(64,(65,(66,(67,68)))))),((69,(70,(71,72))),((75,(76,(73,74))),(77,78))))))))))))),(79,80)),(81,(82,(83,(84,(85,86))))))),(87,(88,89))))),(90,(((91,(92,(93,94))),(95,(96,97))),(98,99)))))))))),(100,101))))))));

TREE tnt_14 = (1,(2,(3,(4,(5,(6,((7,(10,(8,(9,(11,(12,((13,(14,((15,(((((16,(17,18)),(((19,((20,21),(22,(23,24)))),(25,26)),(27,(28,(29,30))))),(31,((33,(34,35)),(((32,36),(37,(38,39))),((40,((41,42),(43,44))),(45,((46,(48,(49,47))),((50,(((53,(54,(56,(55,(57,58))))),(59,60)),(51,52))),((61,((62,63),(64,((65,66),(67,68))))),((69,(70,(71,72))),((75,(76,(73,74))),(77,78)))))))))))),(79,80)),(81,(82,(83,(84,(85,86))))))),(87,(88,89))))),(90,(((91,(92,(93,94))),(95,(96,97))),(98,99)))))))))),(100,101))))))));

TREE tnt_15 = (1,(2,(3,(4,(5,(6,((7,(10,(8,(9,(11,(12,((13,(14,((15,(((((16,(17,18)),(((19,((20,21),(22,(23,24)))),(25,26)),(27,(28,(29,30))))),(31,((33,(34,35)),(((32,36),(37,(38,39))),((40,((41,42),(43,44))),(45,((46,(48,(49,47))),((50,(((53,(54,(56,(55,(57,58))))),(59,60)),(51,52))),((61,((62,63),(64,(65,(66,(67,68)))))),((69,(70,(71,72))),((73,(75,(76,74))),(77,78)))))))))))),(79,80)),(81,(82,(83,(84,(85,86))))))),(87,(88,89))))),(90,(((91,(92,(93,94))),(95,(96,97))),(98,99)))))))))),(100,101))))))));

TREE tnt_16 = (1,(2,(3,(4,(5,(6,((7,((10,(8,9)),(11,(12,((13,(14,((15,(((((16,(17,18)),(((19,((20,21),(22,(23,24)))),(25,26)),(27,(28,(29,30))))),(31,((33,(34,35)),(((32,36),(37,(38,39))),((40,((41,42),(43,44))),(45,((46,(48,(49,47))),((50,(((53,(54,(56,(55,(57,58))))),(59,60)),(51,52))),((61,((62,63),(64,(65,(66,(67,68)))))),((69,(70,(71,72))),((75,(76,(73,74))),(77,78)))))))))))),(79,80)),(81,(82,(83,(84,(85,86))))))),(87,(88,89))))),(90,(((91,(92,(93,94))),(95,(96,97))),(98,99)))))))),(100,101))))))));

TREE tnt_17 = (1,(2,(3,(4,(5,(6,((7,(10,(8,(9,(11,(12,((13,(14,((15,(((((16,(17,18)),(((19,((20,21),(22,(23,24)))),(25,26)),(27,(28,(29,30))))),(31,((33,(34,35)),(((32,36),(37,(38,39))),((40,((41,42),(43,44))),(45,((46,(48,(49,47))),((50,(((53,(54,(56,(55,(57,58))))),(59,60)),(51,52))),((61,((62,63),(64,(65,(66,(67,68)))))),((69,(70,(71,72))),(78,(77,(74,(73,(75,76))))))))))))))),(79,80)),(81,(82,(83,(84,(85,86))))))),(87,(88,89))))),(90,(((91,(92,(93,94))),(95,(96,97))),(98,99)))))))))),(100,101))))))));

TREE tnt_18 = (1,(2,(3,(4,(5,(6,((7,(10,((8,9),(11,(12,((13,(14,((15,(((((16,(17,18)),(((19,((20,21),(22,(23,24)))),(25,26)),(27,(28,(29,30))))),(31,((33,(34,35)),(((32,36),(37,(38,39))),((40,((41,42),(43,44))),(45,((46,(48,(49,47))),((50,(((53,(54,(56,(55,(57,58))))),(59,60)),(51,52))),((61,((62,63),(64,(65,(66,(67,68)))))),((69,(70,(71,72))),((75,(76,(73,74))),(77,78)))))))))))),(79,80)),(81,(82,(83,(84,(85,86))))))),(87,(88,89))))),(90,(((91,(92,(93,94))),(95,(96,97))),(98,99))))))))),(100,101))))))));

TREE tnt_19 = (1,(2,(3,(4,(5,(6,((7,(8,((9,10),(11,(12,((13,(14,((15,(((((16,(17,18)),(((19,((20,21),(22,(23,24)))),(25,26)),(27,(28,(29,30))))),(31,((33,(34,35)),(((32,36),(37,(38,39))),((40,((41,42),(43,44))),(45,((46,(48,(49,47))),((50,(((53,(54,(56,(55,(57,58))))),(59,60)),(51,52))),((61,((62,63),(64,(65,(66,(67,68)))))),((69,(70,(71,72))),((75,(76,(73,74))),(77,78)))))))))))),(79,80)),(81,(82,(83,(84,(85,86))))))),(87,(88,89))))),(90,(((91,(92,(93,94))),(95,(96,97))),(98,99))))))))),(100,101))))))));

TREE tnt_20 = (1,(2,(3,(4,(5,(6,((7,(8,(10,(9,(11,(12,((13,(14,((15,(((((16,(17,18)),(((19,((20,21),(22,(23,24)))),(25,26)),(27,(28,(29,30))))),(31,((33,(34,35)),(((32,36),(37,(38,39))),((40,((41,42),(43,44))),(45,((46,(48,(49,47))),((50,(((53,(54,(56,(55,(57,58))))),(59,60)),(51,52))),((61,((62,63),(64,(65,(66,(67,68)))))),((69,(70,(71,72))),((75,(76,(73,74))),(77,78)))))))))))),(79,80)),(81,(82,(83,(85,(84,86))))))),(87,(88,89))))),(90,(((91,(92,(93,94))),(95,(96,97))),(98,99)))))))))),(100,101))))))));

TREE tnt_21 = (1,(2,(3,(4,(5,(6,((7,(8,(9,(10,(11,(12,((13,(14,((15,(((((16,(17,18)),(((19,((20,21),(22,(23,24)))),(25,26)),(27,(28,(29,30))))),(31,((33,(34,35)),((32,(36,(37,(38,39)))),((40,((41,42),(43,44))),(45,((46,(49,(48,47))),((50,(52,(51,((53,(54,((56,55),(57,58)))),(59,60))))),((61,((62,63),(64,((65,66),(67,68))))),((69,(70,(71,72))),((76,(75,(73,74))),(77,78)))))))))))),(79,80)),(81,(82,(83,(84,(85,86))))))),(87,(88,89))))),(90,(((91,(92,(93,94))),(95,(96,97))),(98,99)))))))))),(100,101))))))));

TREE tnt_22 = (1,(2,(3,(4,(5,(6,((7,(8,(9,(10,(11,(12,((13,(14,((15,(((((16,(17,18)),(((19,((20,21),(22,(23,24)))),(25,26)),(27,(28,(29,30))))),(31,((33,(34,35)),((32,(36,(37,(38,39)))),((40,((41,42),(43,44))),(45,((46,(49,(48,47))),((50,(52,(51,((53,(54,((56,55),(57,58)))),(59,60))))),((61,((62,63),(64,((65,66),(67,68))))),((69,(70,(71,72))),(78,(77,(74,(73,(75,76))))))))))))))),(79,80)),(81,(82,(83,(84,(85,86))))))),(87,(88,89))))),(90,(((91,(92,(93,94))),(95,(96,97))),(98,99)))))))))),(100,101))))))));

TREE tnt_23 = (1,(2,(3,(4,(5,(6,((7,((8,9),(10,(11,(12,((13,(14,((15,(((((16,(17,18)),(((19,((20,21),(22,(23,24)))),(25,26)),(27,(28,(29,30))))),(31,((33,(34,35)),((32,(36,(37,(38,39)))),((40,((41,42),(43,44))),(45,((46,(49,(48,47))),((50,(52,(51,((53,(54,((56,55),(57,58)))),(59,60))))),((61,((62,63),(64,((65,66),(67,68))))),((69,(70,(71,72))),((76,(75,(73,74))),(77,78)))))))))))),(79,80)),(81,(82,(83,(84,(85,86))))))),(87,(88,89))))),(90,(((91,(92,(93,94))),(95,(96,97))),(98,99))))))))),(100,101))))))));

TREE tnt_24 = (1,(2,(3,(4,(5,(6,((7,(8,(10,(9,(11,(12,((13,(14,((15,(((((16,(17,18)),(((19,((20,21),(22,(23,24)))),(25,26)),(27,(28,(29,30))))),(31,((33,(34,35)),((32,(36,(37,(38,39)))),((40,((41,42),(43,44))),(45,((46,(49,(48,47))),((50,(52,(51,((53,(54,((56,55),(57,58)))),(59,60))))),((61,((62,63),(64,((65,66),(67,68))))),((69,(70,(71,72))),((76,(75,(73,74))),(77,78)))))))))))),(79,80)),(81,(82,(83,(84,(85,86))))))),(87,(88,89))))),(90,(((91,(92,(93,94))),(95,(96,97))),(98,99)))))))))),(100,101))))))));

TREE tnt_25 = (1,(2,(3,(4,(5,(6,((7,(8,(9,(10,(11,(12,((13,(14,((15,(((((16,(17,18)),(((19,((20,21),(22,(23,24)))),(25,26)),(27,(28,(29,30))))),(31,((33,(34,35)),((32,(36,(37,(38,39)))),((40,((41,42),(43,44))),(45,((46,(49,(48,47))),((50,(52,(51,((53,(54,((56,55),(57,58)))),(59,60))))),((61,((62,63),(64,((65,66),(67,68))))),((69,(70,(71,72))),((76,(75,(73,74))),(77,78)))))))))))),(79,80)),(81,(82,(83,(85,(84,86))))))),(87,(88,89))))),(90,(((91,(92,(93,94))),(95,(96,97))),(98,99)))))))))),(100,101))))))));

TREE tnt_26 = (1,(2,(3,(4,(5,(6,((7,(8,(9,(10,(11,(12,((13,(14,((15,(((((16,(17,18)),(((19,((20,21),(22,(23,24)))),(25,26)),(27,(28,(29,30))))),(31,(32,((33,(34,35)),((36,(37,(38,39))),((40,((41,42),(43,44))),(45,((46,(49,(48,47))),((50,(52,(51,((53,(54,((56,55),(57,58)))),(59,60))))),((61,((62,63),(64,((65,66),(67,68))))),((69,(70,(71,72))),((76,(75,(73,74))),(77,78))))))))))))),(79,80)),(81,(82,(83,(84,(85,86))))))),(87,(88,89))))),(90,(((91,(92,(93,94))),(95,(96,97))),(98,99)))))))))),(100,101))))))));

TREE tnt_27 = (1,(2,(3,(4,(5,(6,((7,(8,(9,(10,(11,(12,((13,(14,((15,(((((16,(17,18)),(((19,((20,21),(22,(23,24)))),(25,26)),(27,(28,(29,30))))),(31,((33,(34,35)),((32,(36,(37,(38,39)))),((40,((41,42),(43,44))),(45,((46,(49,(48,47))),((50,(52,(51,((53,(54,((56,55),(57,58)))),(59,60))))),((61,((62,63),(64,((65,66),(67,68))))),((69,(70,(71,72))),((73,(76,(75,74))),(77,78)))))))))))),(79,80)),(81,(82,(83,(84,(85,86))))))),(87,(88,89))))),(90,(((91,(92,(93,94))),(95,(96,97))),(98,99)))))))))),(100,101))))))));

TREE tnt_28 = (1,(2,(3,(4,(5,(6,((7,((8,(9,10)),(11,(12,((13,(14,((15,(((((16,(17,18)),(((19,((20,21),(22,(23,24)))),(25,26)),(27,(28,(29,30))))),(31,((33,(34,35)),((32,(36,(37,(38,39)))),((40,((41,42),(43,44))),(45,((46,(49,(48,47))),((50,(52,(51,((53,(54,((56,55),(57,58)))),(59,60))))),((61,((62,63),(64,((65,66),(67,68))))),((69,(70,(71,72))),((76,(75,(73,74))),(77,78)))))))))))),(79,80)),(81,(82,(83,(84,(85,86))))))),(87,(88,89))))),(90,(((91,(92,(93,94))),(95,(96,97))),(98,99)))))))),(100,101))))))));

TREE tnt_29 = (1,(2,(3,(4,(5,(6,((7,((8,9),(10,(11,(12,((13,(14,((15,(((((16,(17,18)),(((19,((20,21),(22,(23,24)))),(25,26)),(27,(28,(29,30))))),(31,((33,(34,35)),((32,(36,(37,(38,39)))),((40,((41,42),(43,44))),(45,((46,(49,(48,47))),((50,(52,(51,((53,(54,((56,55),(57,58)))),(59,60))))),((61,((62,63),(64,((65,66),(67,68))))),((69,(70,(71,72))),(78,(77,(74,(73,(75,76))))))))))))))),(79,80)),(81,(82,(83,(84,(85,86))))))),(87,(88,89))))),(90,(((91,(92,(93,94))),(95,(96,97))),(98,99))))))))),(100,101))))))));

TREE tnt_30 = (1,(2,(3,(4,(5,(6,((7,(10,(8,(9,(11,(12,((13,(14,((15,(((((16,(17,18)),(((19,((20,21),(22,(23,24)))),(25,26)),(27,(28,(29,30))))),(31,((33,(34,35)),((32,(36,(37,(38,39)))),((40,((41,42),(43,44))),(45,((46,(49,(48,47))),((50,(52,(51,((53,(54,((56,55),(57,58)))),(59,60))))),((61,((62,63),(64,((65,66),(67,68))))),((69,(70,(71,72))),(78,(77,(74,(73,(75,76))))))))))))))),(79,80)),(81,(82,(83,(84,(85,86))))))),(87,(88,89))))),(90,(((91,(92,(93,94))),(95,(96,97))),(98,99)))))))))),(100,101))))))));

TREE tnt_31 = (1,(2,(3,(4,(5,(6,((7,((10,(8,9)),(11,(12,((13,(14,((15,(((((16,(17,18)),(((19,((20,21),(22,(23,24)))),(25,26)),(27,(28,(29,30))))),(31,((33,(34,35)),((32,(36,(37,(38,39)))),((40,((41,42),(43,44))),(45,((46,(47,(48,49))),((50,(((53,(54,(55,(56,(57,58))))),(59,60)),(51,52))),((61,((62,63),(64,(65,(66,(67,68)))))),((69,(70,(71,72))),((73,(76,(75,74))),(77,78)))))))))))),(79,80)),(81,(82,(83,(85,(84,86))))))),(87,(88,89))))),(90,(((91,(92,(93,94))),(95,(96,97))),(98,99)))))))),(100,101))))))));

TREE tnt_32 = (1,(2,(3,(4,(5,(6,((7,((10,(8,9)),(11,(12,((13,(14,((15,(((((16,(17,18)),(((19,((20,21),(22,(23,24)))),(25,26)),(27,(28,(29,30))))),(31,((33,(34,35)),((32,(36,(37,(38,39)))),((40,((41,42),(43,44))),(45,((46,(47,(48,49))),((50,(((53,(54,(55,(56,(57,58))))),(59,60)),(51,52))),((61,((62,63),(64,(65,(66,(67,68)))))),((69,(70,(71,72))),((73,(76,(75,74))),(77,78)))))))))))),(79,80)),(81,(82,(83,(84,(85,86))))))),(87,(88,89))))),(90,(((91,(92,(93,94))),(95,(96,97))),(98,99)))))))),(100,101))))))));

TREE tnt_33 = (1,(2,(3,(4,(5,(6,((7,((10,(8,9)),(11,(12,((13,(14,((15,(((((16,(17,18)),(((19,((20,21),(22,(23,24)))),(25,26)),(27,(28,(29,30))))),(31,(32,((33,(34,35)),((36,(37,(38,39))),((40,((41,42),(43,44))),(45,((46,(47,(48,49))),((50,(((53,(54,(55,(56,(57,58))))),(59,60)),(51,52))),((61,((62,63),(64,(65,(66,(67,68)))))),((69,(70,(71,72))),((73,(76,(75,74))),(77,78))))))))))))),(79,80)),(81,(82,(83,(85,(84,86))))))),(87,(88,89))))),(90,(((91,(92,(93,94))),(95,(96,97))),(98,99)))))))),(100,101))))))));

TREE tnt_34 = (1,(2,(3,(4,(5,(6,((7,((10,(8,9)),(11,(12,((13,(14,((15,(((((16,(17,18)),(((19,((20,21),(22,(23,24)))),(25,26)),(27,(28,(29,30))))),(31,((33,(34,35)),((32,(36,(37,(38,39)))),((40,((41,42),(43,44))),(45,((46,(47,(48,49))),((50,(((53,(54,(55,(56,(57,58))))),(59,60)),(51,52))),((61,((62,63),(64,((65,66),(67,68))))),((69,(70,(71,72))),((73,(76,(75,74))),(77,78)))))))))))),(79,80)),(81,(82,(83,(85,(84,86))))))),(87,(88,89))))),(90,(((91,(92,(93,94))),(95,(96,97))),(98,99)))))))),(100,101))))))));

TREE tnt_35 = (1,(2,(3,(4,(5,(6,((7,((10,(8,9)),(11,(12,((13,(14,((15,(((((16,(17,18)),(((19,((20,21),(22,(23,24)))),(25,26)),(27,(28,(29,30))))),(31,((33,(34,35)),((32,(36,(37,(38,39)))),((40,((41,42),(43,44))),(45,((46,(47,(48,49))),((50,(((53,(54,(55,(56,(57,58))))),(59,60)),(51,52))),((61,((62,63),(64,(65,(66,(67,68)))))),((69,(70,(71,72))),(78,(77,(74,(75,(73,76))))))))))))))),(79,80)),(81,(82,(83,(85,(84,86))))))),(87,(88,89))))),(90,(((91,(92,(93,94))),(95,(96,97))),(98,99)))))))),(100,101))))))));

TREE tnt_36 = (1,(2,(3,(4,(5,(6,((7,((8,9),(10,(11,(12,((13,(14,((15,(((((16,(17,18)),(((19,((20,21),(22,(23,24)))),(25,26)),(27,(28,(29,30))))),(31,((33,(34,35)),((32,(36,(37,(38,39)))),((40,((41,42),(43,44))),(45,((46,(47,(48,49))),((50,(((53,(54,(55,(56,(57,58))))),(59,60)),(51,52))),((61,((62,63),(64,(65,(66,(67,68)))))),((69,(70,(71,72))),((73,(76,(75,74))),(77,78)))))))))))),(79,80)),(81,(82,(83,(85,(84,86))))))),(87,(88,89))))),(90,(((91,(92,(93,94))),(95,(96,97))),(98,99))))))))),(100,101))))))));

TREE tnt_37 = (1,(2,(3,(4,(5,(6,((7,(8,((9,10),(11,(12,((13,(14,((15,(((((16,(17,18)),(((19,((20,21),(22,(23,24)))),(25,26)),(27,(28,(29,30))))),(31,((33,(34,35)),((32,(36,(37,(38,39)))),((40,((41,42),(43,44))),(45,((46,(47,(48,49))),((50,(((53,(54,(55,(56,(57,58))))),(59,60)),(51,52))),((61,((62,63),(64,(65,(66,(67,68)))))),((69,(70,(71,72))),((73,(76,(75,74))),(77,78)))))))))))),(79,80)),(81,(82,(83,(85,(84,86))))))),(87,(88,89))))),(90,(((91,(92,(93,94))),(95,(96,97))),(98,99))))))))),(100,101))))))));

TREE tnt_38 = (1,(2,(3,(4,(5,(6,((7,(8,((9,10),(11,(12,((13,(14,((15,(((((16,(17,18)),(((19,((20,21),(22,(23,24)))),(25,26)),(27,(28,(29,30))))),(31,((33,(34,35)),((32,(36,(37,(38,39)))),((40,((41,42),(43,44))),(45,((46,(47,(48,49))),((50,(((53,(54,(55,(56,(57,58))))),(59,60)),(51,52))),((61,((62,63),(64,(65,(66,(67,68)))))),((69,(70,(71,72))),((73,(76,(75,74))),(77,78)))))))))))),(79,80)),(81,(82,(83,(84,(85,86))))))),(87,(88,89))))),(90,(((91,(92,(93,94))),(95,(96,97))),(98,99))))))))),(100,101))))))));

TREE tnt_39 = (1,(2,(3,(4,(5,(6,((7,(10,((8,9),(11,(12,((13,(14,((15,(((((16,(17,18)),(((19,((20,21),(22,(23,24)))),(25,26)),(27,(28,(29,30))))),(31,((33,(34,35)),((32,(36,(37,(38,39)))),((40,((41,42),(43,44))),(45,((46,(47,(48,49))),((50,(((53,(54,(55,(56,(57,58))))),(59,60)),(51,52))),((61,((62,63),(64,(65,(66,(67,68)))))),((69,(70,(71,72))),((73,(76,(75,74))),(77,78)))))))))))),(79,80)),(81,(82,(83,(84,(85,86))))))),(87,(88,89))))),(90,(((91,(92,(93,94))),(95,(96,97))),(98,99))))))))),(100,101))))))));

TREE tnt_40 = (1,(2,(3,(4,(5,(6,((7,((10,(8,9)),(11,(12,((13,(14,((15,(((((16,(17,18)),(((19,((20,21),(22,(23,24)))),(25,26)),(27,(28,(29,30))))),(31,((33,(34,35)),((32,(36,(37,(38,39)))),((40,((41,42),(43,44))),(45,((46,(47,(48,49))),((50,(((53,(54,(55,(56,(57,58))))),(59,60)),(51,52))),((61,((62,63),(64,((65,66),(67,68))))),((69,(70,(71,72))),((73,(76,(75,74))),(77,78)))))))))))),(79,80)),(81,(82,(83,(84,(85,86))))))),(87,(88,89))))),(90,(((91,(92,(93,94))),(95,(96,97))),(98,99)))))))),(100,101))))))));

TREE tnt_41 = (1,(2,(3,(4,(5,(6,((7,(10,(8,(9,(11,(12,((13,(14,((15,(((((16,(17,18)),(((19,((20,21),(22,(23,24)))),(25,26)),(27,(28,(29,30))))),(31,(32,((33,(34,35)),((36,(37,(38,39))),((40,((41,42),(43,44))),(45,((46,(48,(49,47))),((50,(52,(51,((53,(54,((56,55),(57,58)))),(59,60))))),((61,((62,63),(64,((65,66),(67,68))))),((69,(70,(71,72))),((73,(76,(75,74))),(77,78))))))))))))),(79,80)),(81,(82,(83,(84,(85,86))))))),(87,(88,89))))),(90,(((91,(92,(93,94))),(95,(96,97))),(98,99)))))))))),(100,101))))))));

TREE tnt_42 = (1,(2,(3,(4,(5,(6,((7,(10,(8,(9,(11,(12,((13,(14,((15,(((((16,(17,18)),(((19,((20,21),(22,(23,24)))),(25,26)),(27,(28,(29,30))))),(31,(32,((33,(34,35)),((36,(37,(38,39))),((40,((41,42),(43,44))),(45,((46,(48,(49,47))),((50,(52,(51,((53,(54,((56,55),(57,58)))),(59,60))))),((61,((62,63),(64,((65,66),(67,68))))),((69,(70,(71,72))),((73,(76,(75,74))),(77,78))))))))))))),(79,80)),(81,(82,(83,(85,(84,86))))))),(87,(88,89))))),(90,(((91,(92,(93,94))),(95,(96,97))),(98,99)))))))))),(100,101))))))));

TREE tnt_43 = (1,(2,(3,(4,(5,(6,((7,(10,(8,(9,(11,(12,((13,(14,((15,(((((16,(17,18)),(((19,((20,21),(22,(23,24)))),(25,26)),(27,(28,(29,30))))),(31,((33,(34,35)),((32,(36,(37,(38,39)))),((40,((41,42),(43,44))),(45,((46,(48,(49,47))),((50,(52,(51,((53,(54,((56,55),(57,58)))),(59,60))))),((61,((62,63),(64,((65,66),(67,68))))),((69,(70,(71,72))),((73,(76,(75,74))),(77,78)))))))))))),(79,80)),(81,(82,(83,(84,(85,86))))))),(87,(88,89))))),(90,(((91,(92,(93,94))),(95,(96,97))),(98,99)))))))))),(100,101))))))));

TREE tnt_44 = (1,(2,(3,(4,(5,(6,((7,(10,(8,(9,(11,(12,((13,(14,((15,(((((16,(17,18)),(((19,((20,21),(22,(23,24)))),(25,26)),(27,(28,(29,30))))),(31,(32,((33,(34,35)),((36,(37,(38,39))),((40,((41,42),(43,44))),(45,((46,(48,(49,47))),((50,(52,(51,((53,(54,((56,55),(57,58)))),(59,60))))),((61,((62,63),(64,((65,66),(67,68))))),((69,(70,(71,72))),((76,(75,(73,74))),(77,78))))))))))))),(79,80)),(81,(82,(83,(84,(85,86))))))),(87,(88,89))))),(90,(((91,(92,(93,94))),(95,(96,97))),(98,99)))))))))),(100,101))))))));

TREE tnt_45 = (1,(2,(3,(4,(5,(6,((7,((10,(8,9)),(11,(12,((13,(14,((15,(((((16,(17,18)),(((19,((20,21),(22,(23,24)))),(25,26)),(27,(28,(29,30))))),(31,(32,((33,(34,35)),((36,(37,(38,39))),((40,((41,42),(43,44))),(45,((46,(48,(49,47))),((50,(52,(51,((53,(54,((56,55),(57,58)))),(59,60))))),((61,((62,63),(64,((65,66),(67,68))))),((69,(70,(71,72))),((73,(76,(75,74))),(77,78))))))))))))),(79,80)),(81,(82,(83,(84,(85,86))))))),(87,(88,89))))),(90,(((91,(92,(93,94))),(95,(96,97))),(98,99)))))))),(100,101))))))));

TREE tnt_46 = (1,(2,(3,(4,(5,(6,((7,(10,(8,(9,(11,(12,((13,(14,((15,(((((16,(17,18)),(((19,((20,21),(22,(23,24)))),(25,26)),(27,(28,(29,30))))),(31,(32,((33,(34,35)),((36,(37,(38,39))),((40,((41,42),(43,44))),(45,((46,(48,(49,47))),((50,(52,(51,((53,(54,((56,55),(57,58)))),(59,60))))),((61,((62,63),(64,((65,66),(67,68))))),((69,(70,(71,72))),(78,(77,(74,(75,(73,76)))))))))))))))),(79,80)),(81,(82,(83,(84,(85,86))))))),(87,(88,89))))),(90,(((91,(92,(93,94))),(95,(96,97))),(98,99)))))))))),(100,101))))))));

TREE tnt_47 = (1,(2,(3,(4,(5,(6,((7,(10,((8,9),(11,(12,((13,(14,((15,(((((16,(17,18)),(((19,((20,21),(22,(23,24)))),(25,26)),(27,(28,(29,30))))),(31,(32,((33,(34,35)),((36,(37,(38,39))),((40,((41,42),(43,44))),(45,((46,(48,(49,47))),((50,(52,(51,((53,(54,((56,55),(57,58)))),(59,60))))),((61,((62,63),(64,((65,66),(67,68))))),((69,(70,(71,72))),((73,(76,(75,74))),(77,78))))))))))))),(79,80)),(81,(82,(83,(84,(85,86))))))),(87,(88,89))))),(90,(((91,(92,(93,94))),(95,(96,97))),(98,99))))))))),(100,101))))))));

TREE tnt_48 = (1,(2,(3,(4,(5,(6,((7,(8,((9,10),(11,(12,((13,(14,((15,(((((16,(17,18)),(((19,((20,21),(22,(23,24)))),(25,26)),(27,(28,(29,30))))),(31,(32,((33,(34,35)),((36,(37,(38,39))),((40,((41,42),(43,44))),(45,((46,(48,(49,47))),((50,(52,(51,((53,(54,((56,55),(57,58)))),(59,60))))),((61,((62,63),(64,((65,66),(67,68))))),((69,(70,(71,72))),((73,(76,(75,74))),(77,78))))))))))))),(79,80)),(81,(82,(83,(84,(85,86))))))),(87,(88,89))))),(90,(((91,(92,(93,94))),(95,(96,97))),(98,99))))))))),(100,101))))))));

TREE tnt_49 = (1,(2,(3,(4,(5,(6,((7,(8,(10,(9,(11,(12,((13,(14,((15,(((((16,(17,18)),(((19,((20,21),(22,(23,24)))),(25,26)),(27,(28,(29,30))))),(31,(32,((33,(34,35)),((36,(37,(38,39))),((40,((41,42),(43,44))),(45,((46,(48,(49,47))),((50,(52,(51,((53,(54,((56,55),(57,58)))),(59,60))))),((61,((62,63),(64,((65,66),(67,68))))),((69,(70,(71,72))),((73,(76,(75,74))),(77,78))))))))))))),(79,80)),(81,(82,(83,(85,(84,86))))))),(87,(88,89))))),(90,(((91,(92,(93,94))),(95,(96,97))),(98,99)))))))))),(100,101))))))));

TREE tnt_50 = (1,(2,(3,(4,(5,(6,((7,(10,((8,9),(11,(12,((13,(14,((15,(((((16,(17,18)),(((19,((20,21),(22,(23,24)))),(25,26)),(27,(28,(29,30))))),(31,(32,((33,(34,35)),((36,(37,(38,39))),((40,((41,42),(43,44))),(45,((46,(48,(49,47))),((50,(52,(51,((53,(54,((56,55),(57,58)))),(59,60))))),((61,((62,63),(64,((65,66),(67,68))))),((69,(70,(71,72))),((73,(76,(75,74))),(77,78))))))))))))),(79,80)),(81,(82,(83,(85,(84,86))))))),(87,(88,89))))),(90,(((91,(92,(93,94))),(95,(96,97))),(98,99))))))))),(100,101))))))));

TREE tnt_51 = (1,(2,(3,(4,(5,(6,((7,(8,(10,(9,(11,(12,((13,(14,((15,(((((16,(17,18)),(((19,((20,21),(22,(23,24)))),(25,26)),(27,(28,(29,30))))),(31,((33,(34,35)),((32,(36,(37,(38,39)))),((40,((41,42),(43,44))),(45,((46,(48,(49,47))),((50,(((53,(54,(55,(56,(57,58))))),(59,60)),(51,52))),((61,((62,63),(64,(65,(66,(67,68)))))),((69,(70,(71,72))),(78,(77,(74,(76,(75,73))))))))))))))),(79,80)),(81,(82,(83,(84,(85,86))))))),(87,(88,89))))),(90,(((91,(92,(93,94))),(95,(96,97))),(98,99)))))))))),(100,101))))))));

TREE tnt_52 = (1,(2,(3,(4,(5,(6,((7,(8,(10,(9,(11,(12,((13,(14,((15,(((((16,(17,18)),(((19,((20,21),(22,(23,24)))),(25,26)),(27,(28,(29,30))))),(31,((33,(34,35)),((32,(36,(37,(38,39)))),((40,((41,42),(43,44))),(45,((46,(48,(49,47))),((50,(((53,(54,(55,(56,(57,58))))),(59,60)),(51,52))),((61,((62,63),(64,((65,66),(67,68))))),((69,(70,(71,72))),(78,(77,(74,(76,(75,73))))))))))))))),(79,80)),(81,(82,(83,(84,(85,86))))))),(87,(88,89))))),(90,(((91,(92,(93,94))),(95,(96,97))),(98,99)))))))))),(100,101))))))));

TREE tnt_53 = (1,(2,(3,(4,(5,(6,((7,(8,(10,(9,(11,(12,((13,(14,((15,(((((16,(17,18)),(((19,((20,21),(22,(23,24)))),(25,26)),(27,(28,(29,30))))),(31,(32,((33,(34,35)),((36,(37,(38,39))),((40,((41,42),(43,44))),(45,((46,(48,(49,47))),((50,(((53,(54,(55,(56,(57,58))))),(59,60)),(51,52))),((61,((62,63),(64,(65,(66,(67,68)))))),((69,(70,(71,72))),(78,(77,(74,(76,(75,73)))))))))))))))),(79,80)),(81,(82,(83,(84,(85,86))))))),(87,(88,89))))),(90,(((91,(92,(93,94))),(95,(96,97))),(98,99)))))))))),(100,101))))))));

TREE tnt_54 = (1,(2,(3,(4,(5,(6,((7,((8,(9,10)),(11,(12,((13,(14,((15,(((((16,(17,18)),(((19,((20,21),(22,(23,24)))),(25,26)),(27,(28,(29,30))))),(31,((33,(34,35)),((32,(36,(37,(38,39)))),((40,((41,42),(43,44))),(45,((46,(48,(49,47))),((50,(((53,(54,(55,(56,(57,58))))),(59,60)),(51,52))),((61,((62,63),(64,(65,(66,(67,68)))))),((69,(70,(71,72))),(78,(77,(74,(76,(75,73))))))))))))))),(79,80)),(81,(82,(83,(84,(85,86))))))),(87,(88,89))))),(90,(((91,(92,(93,94))),(95,(96,97))),(98,99)))))))),(100,101))))))));

TREE tnt_55 = (1,(2,(3,(4,(5,(6,((7,(8,(10,(9,(11,(12,((13,(14,((15,(((((16,(17,18)),(((19,((20,21),(22,(23,24)))),(25,26)),(27,(28,(29,30))))),(31,((33,(34,35)),((32,(36,(37,(38,39)))),((40,((41,42),(43,44))),(45,((46,(48,(49,47))),((50,(((53,(54,(55,(56,(57,58))))),(59,60)),(51,52))),((61,((62,63),(64,((65,66),(67,68))))),((69,(70,(71,72))),(78,(77,(74,(76,(75,73))))))))))))))),(79,80)),(81,(82,(83,(85,(84,86))))))),(87,(88,89))))),(90,(((91,(92,(93,94))),(95,(96,97))),(98,99)))))))))),(100,101))))))));

TREE tnt_56 = (1,(2,(3,(4,(5,(6,((7,(8,(10,(9,(11,(12,((13,(14,((15,(((((16,(17,18)),(((19,((20,21),(22,(23,24)))),(25,26)),(27,(28,(29,30))))),(31,(32,((33,(34,35)),((36,(37,(38,39))),((40,((41,42),(43,44))),(45,((46,(48,(49,47))),((50,(((53,(54,(55,(56,(57,58))))),(59,60)),(51,52))),((61,((62,63),(64,((65,66),(67,68))))),((69,(70,(71,72))),(78,(77,(74,(76,(75,73)))))))))))))))),(79,80)),(81,(82,(83,(84,(85,86))))))),(87,(88,89))))),(90,(((91,(92,(93,94))),(95,(96,97))),(98,99)))))))))),(100,101))))))));

TREE tnt_57 = (1,(2,(3,(4,(5,(6,((7,((8,(9,10)),(11,(12,((13,(14,((15,(((((16,(17,18)),(((19,((20,21),(22,(23,24)))),(25,26)),(27,(28,(29,30))))),(31,((33,(34,35)),((32,(36,(37,(38,39)))),((40,((41,42),(43,44))),(45,((46,(48,(49,47))),((50,(((53,(54,(55,(56,(57,58))))),(59,60)),(51,52))),((61,((62,63),(64,((65,66),(67,68))))),((69,(70,(71,72))),(78,(77,(74,(76,(75,73))))))))))))))),(79,80)),(81,(82,(83,(84,(85,86))))))),(87,(88,89))))),(90,(((91,(92,(93,94))),(95,(96,97))),(98,99)))))))),(100,101))))))));

TREE tnt_58 = (1,(2,(3,(4,(5,(6,((7,(10,(8,(9,(11,(12,((13,(14,((15,(((((16,(17,18)),(((19,((20,21),(22,(23,24)))),(25,26)),(27,(28,(29,30))))),(31,(32,((33,(34,35)),((36,(37,(38,39))),((40,((41,42),(43,44))),(45,((46,(48,(49,47))),((50,(((53,(54,(55,(56,(57,58))))),(59,60)),(51,52))),((61,((62,63),(64,(65,(66,(67,68)))))),((69,(70,(71,72))),(78,(77,(74,(76,(75,73)))))))))))))))),(79,80)),(81,(82,(83,(84,(85,86))))))),(87,(88,89))))),(90,(((91,(92,(93,94))),(95,(96,97))),(98,99)))))))))),(100,101))))))));

TREE tnt_59 = (1,(2,(3,(4,(5,(6,((7,(8,(10,(9,(11,(12,((13,(14,((15,(((((16,(17,18)),(((19,((20,21),(22,(23,24)))),(25,26)),(27,(28,(29,30))))),(31,(32,((33,(34,35)),((36,(37,(38,39))),((40,((41,42),(43,44))),(45,((46,(48,(49,47))),((50,(((53,(54,(55,(56,(57,58))))),(59,60)),(51,52))),((61,((62,63),(64,(65,(66,(67,68)))))),((69,(70,(71,72))),(78,(77,(74,(76,(75,73)))))))))))))))),(79,80)),(81,(82,(83,(85,(84,86))))))),(87,(88,89))))),(90,(((91,(92,(93,94))),(95,(96,97))),(98,99)))))))))),(100,101))))))));

TREE tnt_60 = (1,(2,(3,(4,(5,(6,((7,(8,((9,10),(11,(12,((13,(14,((15,(((((16,(17,18)),(((19,((20,21),(22,(23,24)))),(25,26)),(27,(28,(29,30))))),(31,((33,(34,35)),((32,(36,(37,(38,39)))),((40,((41,42),(43,44))),(45,((46,(48,(49,47))),((50,(((53,(54,(55,(56,(57,58))))),(59,60)),(51,52))),((61,((62,63),(64,(65,(66,(67,68)))))),((69,(70,(71,72))),(78,(77,(74,(76,(75,73))))))))))))))),(79,80)),(81,(82,(83,(84,(85,86))))))),(87,(88,89))))),(90,(((91,(92,(93,94))),(95,(96,97))),(98,99))))))))),(100,101))))))));

TREE tnt_61 = (1,(2,(3,(4,(5,(6,((7,(10,((8,9),(11,(12,((13,(14,((15,(((((16,(17,18)),(((19,((20,21),(22,(23,24)))),(25,26)),(27,(28,(29,30))))),(31,(32,((33,(34,35)),((36,(37,(38,39))),((40,((41,42),(43,44))),(45,((46,(47,(48,49))),((50,(51,(52,((53,(54,(55,(56,(57,58))))),(59,60))))),((61,((62,63),(64,(65,(66,(67,68)))))),((69,(70,(71,72))),((73,(75,(76,74))),(77,78))))))))))))),(79,80)),(81,(82,(83,(85,(84,86))))))),(87,(88,89))))),(90,(((91,(92,(93,94))),(95,(96,97))),(98,99))))))))),(100,101))))))));

TREE tnt_62 = (1,(2,(3,(4,(5,(6,((7,(10,((8,9),(11,(12,((13,(14,((15,(((((16,(17,18)),(((19,((20,21),(22,(23,24)))),(25,26)),(27,(28,(29,30))))),(31,(32,((33,(34,35)),((36,(37,(38,39))),((40,((41,42),(43,44))),(45,((46,(47,(48,49))),((50,(51,(52,((53,(54,(55,(56,(57,58))))),(59,60))))),((61,((62,63),(64,(65,(66,(67,68)))))),((69,(70,(71,72))),(78,(77,(74,(76,(75,73)))))))))))))))),(79,80)),(81,(82,(83,(85,(84,86))))))),(87,(88,89))))),(90,(((91,(92,(93,94))),(95,(96,97))),(98,99))))))))),(100,101))))))));

TREE tnt_63 = (1,(2,(3,(4,(5,(6,((7,(10,((8,9),(11,(12,((13,(14,((15,(((((16,(17,18)),(((19,((20,21),(22,(23,24)))),(25,26)),(27,(28,(29,30))))),(31,(32,((33,(34,35)),((36,(37,(38,39))),((40,((41,42),(43,44))),(45,((46,(47,(48,49))),((50,(51,(52,((53,(54,(55,(56,(57,58))))),(59,60))))),((61,((62,63),(64,(65,(66,(67,68)))))),((69,(70,(71,72))),((75,(76,(73,74))),(77,78))))))))))))),(79,80)),(81,(82,(83,(85,(84,86))))))),(87,(88,89))))),(90,(((91,(92,(93,94))),(95,(96,97))),(98,99))))))))),(100,101))))))));

TREE tnt_64 = (1,(2,(3,(4,(5,(6,((7,(10,(8,(9,(11,(12,((13,(14,((15,(((((16,(17,18)),(((19,((20,21),(22,(23,24)))),(25,26)),(27,(28,(29,30))))),(31,(32,((33,(34,35)),((36,(37,(38,39))),((40,((41,42),(43,44))),(45,((46,(47,(48,49))),((50,(51,(52,((53,(54,(55,(56,(57,58))))),(59,60))))),((61,((62,63),(64,(65,(66,(67,68)))))),((69,(70,(71,72))),((73,(75,(76,74))),(77,78))))))))))))),(79,80)),(81,(82,(83,(85,(84,86))))))),(87,(88,89))))),(90,(((91,(92,(93,94))),(95,(96,97))),(98,99)))))))))),(100,101))))))));

TREE tnt_65 = (1,(2,(3,(4,(5,(6,((7,((9,(8,10)),(11,(12,((13,(14,((15,(((((16,(17,18)),(((19,((20,21),(22,(23,24)))),(25,26)),(27,(28,(29,30))))),(31,(32,((33,(34,35)),((36,(37,(38,39))),((40,((41,42),(43,44))),(45,((46,(47,(48,49))),((50,(51,(52,((53,(54,(55,(56,(57,58))))),(59,60))))),((61,((62,63),(64,(65,(66,(67,68)))))),((69,(70,(71,72))),(78,(77,(74,(76,(75,73)))))))))))))))),(79,80)),(81,(82,(83,(85,(84,86))))))),(87,(88,89))))),(90,(((91,(92,(93,94))),(95,(96,97))),(98,99)))))))),(100,101))))))));

TREE tnt_66 = (1,(2,(3,(4,(5,(6,((7,(10,((8,9),(11,(12,((13,(14,((15,(((((16,(17,18)),(((19,((20,21),(22,(23,24)))),(25,26)),(27,(28,(29,30))))),(31,(32,((33,(34,35)),((36,(37,(38,39))),((40,((41,42),(43,44))),(45,((46,(47,(48,49))),((50,(51,(52,((53,(54,(55,(56,(57,58))))),(59,60))))),((61,((62,63),(64,(65,(66,(67,68)))))),((69,(70,(71,72))),(78,(77,(74,(76,(75,73)))))))))))))))),(79,80)),(81,(82,(83,(84,(85,86))))))),(87,(88,89))))),(90,(((91,(92,(93,94))),(95,(96,97))),(98,99))))))))),(100,101))))))));

TREE tnt_67 = (1,(2,(3,(4,(5,(6,((7,(10,((8,9),(11,(12,((13,(14,((15,(((((16,(17,18)),(((19,((20,21),(22,(23,24)))),(25,26)),(27,(28,(29,30))))),(31,((33,(34,35)),((32,(36,(37,(38,39)))),((40,((41,42),(43,44))),(45,((46,(47,(48,49))),((50,(51,(52,((53,(54,(55,(56,(57,58))))),(59,60))))),((61,((62,63),(64,(65,(66,(67,68)))))),((69,(70,(71,72))),(78,(77,(74,(76,(75,73))))))))))))))),(79,80)),(81,(82,(83,(85,(84,86))))))),(87,(88,89))))),(90,(((91,(92,(93,94))),(95,(96,97))),(98,99))))))))),(100,101))))))));

TREE tnt_68 = (1,(2,(3,(4,(5,(6,((7,(10,((8,9),(11,(12,((13,(14,((15,(((((16,(17,18)),(((19,((20,21),(22,(23,24)))),(25,26)),(27,(28,(29,30))))),(31,(32,((33,(34,35)),((36,(37,(38,39))),((40,((41,42),(43,44))),(45,((46,(47,(48,49))),((50,(51,(52,((53,(54,(55,(56,(57,58))))),(59,60))))),((61,((62,63),(64,((65,66),(67,68))))),((69,(70,(71,72))),(78,(77,(74,(76,(75,73)))))))))))))))),(79,80)),(81,(82,(83,(85,(84,86))))))),(87,(88,89))))),(90,(((91,(92,(93,94))),(95,(96,97))),(98,99))))))))),(100,101))))))));

TREE tnt_69 = (1,(2,(3,(4,(5,(6,((7,(10,(8,(9,(11,(12,((13,(14,((15,(((((16,(17,18)),(((19,((20,21),(22,(23,24)))),(25,26)),(27,(28,(29,30))))),(31,(32,((33,(34,35)),((36,(37,(38,39))),((40,((41,42),(43,44))),(45,((46,(47,(48,49))),((50,(51,(52,((53,(54,(55,(56,(57,58))))),(59,60))))),((61,((62,63),(64,(65,(66,(67,68)))))),((69,(70,(71,72))),(78,(77,(74,(76,(75,73)))))))))))))))),(79,80)),(81,(82,(83,(85,(84,86))))))),(87,(88,89))))),(90,(((91,(92,(93,94))),(95,(96,97))),(98,99)))))))))),(100,101))))))));

TREE tnt_70 = (1,(2,(3,(4,(5,(6,((7,(8,(10,(9,(11,(12,((13,(14,((15,(((((16,(17,18)),(((19,((20,21),(22,(23,24)))),(25,26)),(27,(28,(29,30))))),(31,(32,((33,(34,35)),((36,(37,(38,39))),((40,((41,42),(43,44))),(45,((46,(47,(48,49))),((50,(51,(52,((53,(54,(55,(56,(57,58))))),(59,60))))),((61,((62,63),(64,(65,(66,(67,68)))))),((69,(70,(71,72))),((75,(76,(73,74))),(77,78))))))))))))),(79,80)),(81,(82,(83,(85,(84,86))))))),(87,(88,89))))),(90,(((91,(92,(93,94))),(95,(96,97))),(98,99)))))))))),(100,101))))))));

TREE tnt_71 = (1,(2,(3,(4,(5,(6,((7,(8,(9,(10,(11,(12,((13,(14,((15,(((((16,(17,18)),(((19,((20,21),(22,(23,24)))),(25,26)),(27,(28,(29,30))))),(31,((33,(34,35)),((32,(36,(37,(38,39)))),((40,((41,42),(43,44))),(45,((46,(49,(48,47))),((50,(51,(52,((53,(54,(55,(56,(57,58))))),(59,60))))),((61,((62,63),(64,(65,(66,(67,68)))))),((69,(70,(71,72))),(78,(77,(74,(75,(73,76))))))))))))))),(79,80)),(81,(82,(83,(85,(84,86))))))),(87,(88,89))))),(90,(((91,(92,(93,94))),(95,(96,97))),(98,99)))))))))),(100,101))))))));

TREE tnt_72 = (1,(2,(3,(4,(5,(6,((7,(8,(9,(10,(11,(12,((13,(14,((15,(((((16,(17,18)),(((19,((20,21),(22,(23,24)))),(25,26)),(27,(28,(29,30))))),(31,(32,((33,(34,35)),((36,(37,(38,39))),((40,((41,42),(43,44))),(45,((46,(49,(48,47))),((50,(51,(52,((53,(54,(55,(56,(57,58))))),(59,60))))),((61,((62,63),(64,(65,(66,(67,68)))))),((69,(70,(71,72))),(78,(77,(74,(75,(73,76)))))))))))))))),(79,80)),(81,(82,(83,(85,(84,86))))))),(87,(88,89))))),(90,(((91,(92,(93,94))),(95,(96,97))),(98,99)))))))))),(100,101))))))));

TREE tnt_73 = (1,(2,(3,(4,(5,(6,((7,(8,(9,(10,(11,(12,((13,(14,((15,(((((16,(17,18)),(((19,((20,21),(22,(23,24)))),(25,26)),(27,(28,(29,30))))),(31,((33,(34,35)),((32,(36,(37,(38,39)))),((40,((41,42),(43,44))),(45,((46,(49,(48,47))),((50,(51,(52,((53,(54,(55,(56,(57,58))))),(59,60))))),((61,((62,63),(64,((65,66),(67,68))))),((69,(70,(71,72))),(78,(77,(74,(75,(73,76))))))))))))))),(79,80)),(81,(82,(83,(85,(84,86))))))),(87,(88,89))))),(90,(((91,(92,(93,94))),(95,(96,97))),(98,99)))))))))),(100,101))))))));

TREE tnt_74 = (1,(2,(3,(4,(5,(6,((7,((8,(9,10)),(11,(12,((13,(14,((15,(((((16,(17,18)),(((19,((20,21),(22,(23,24)))),(25,26)),(27,(28,(29,30))))),(31,((33,(34,35)),((32,(36,(37,(38,39)))),((40,((41,42),(43,44))),(45,((46,(49,(48,47))),((50,(51,(52,((53,(54,(55,(56,(57,58))))),(59,60))))),((61,((62,63),(64,(65,(66,(67,68)))))),((69,(70,(71,72))),(78,(77,(74,(75,(73,76))))))))))))))),(79,80)),(81,(82,(83,(85,(84,86))))))),(87,(88,89))))),(90,(((91,(92,(93,94))),(95,(96,97))),(98,99)))))))),(100,101))))))));

TREE tnt_75 = (1,(2,(3,(4,(5,(6,((7,(10,(8,(9,(11,(12,((13,(14,((15,(((((16,(17,18)),(((19,((20,21),(22,(23,24)))),(25,26)),(27,(28,(29,30))))),(31,((33,(34,35)),((32,(36,(37,(38,39)))),((40,((41,42),(43,44))),(45,((46,(49,(48,47))),((50,(51,(52,((53,(54,(55,(56,(57,58))))),(59,60))))),((61,((62,63),(64,(65,(66,(67,68)))))),((69,(70,(71,72))),(78,(77,(74,(75,(73,76))))))))))))))),(79,80)),(81,(82,(83,(85,(84,86))))))),(87,(88,89))))),(90,(((91,(92,(93,94))),(95,(96,97))),(98,99)))))))))),(100,101))))))));

TREE tnt_76 = (1,(2,(3,(4,(5,(6,((7,(8,(9,(10,(11,(12,((13,(14,((15,(((((16,(17,18)),(((19,((20,21),(22,(23,24)))),(25,26)),(27,(28,(29,30))))),(31,(32,((33,(34,35)),((36,(37,(38,39))),((40,((41,42),(43,44))),(45,((46,(49,(48,47))),((50,(51,(52,((53,(54,(55,(56,(57,58))))),(59,60))))),((61,((62,63),(64,(65,(66,(67,68)))))),((69,(70,(71,72))),(78,(77,(74,(75,(73,76)))))))))))))))),(79,80)),(81,(82,(83,(84,(85,86))))))),(87,(88,89))))),(90,(((91,(92,(93,94))),(95,(96,97))),(98,99)))))))))),(100,101))))))));

TREE tnt_77 = (1,(2,(3,(4,(5,(6,((7,(8,(9,(10,(11,(12,((13,(14,((15,(((((16,(17,18)),(((19,((20,21),(22,(23,24)))),(25,26)),(27,(28,(29,30))))),(31,(32,((33,(34,35)),((36,(37,(38,39))),((40,((41,42),(43,44))),(45,((46,(49,(48,47))),((50,(51,(52,((53,(54,(55,(56,(57,58))))),(59,60))))),((61,((62,63),(64,((65,66),(67,68))))),((69,(70,(71,72))),(78,(77,(74,(75,(73,76)))))))))))))))),(79,80)),(81,(82,(83,(85,(84,86))))))),(87,(88,89))))),(90,(((91,(92,(93,94))),(95,(96,97))),(98,99)))))))))),(100,101))))))));

TREE tnt_78 = (1,(2,(3,(4,(5,(6,((7,(8,(9,(10,(11,(12,((13,(14,((15,(((((16,(17,18)),(((19,((20,21),(22,(23,24)))),(25,26)),(27,(28,(29,30))))),(31,(32,((33,(34,35)),((36,(37,(38,39))),((40,((41,42),(43,44))),(45,((46,(49,(48,47))),((50,(51,(52,((53,(54,(55,(56,(57,58))))),(59,60))))),((61,((62,63),(64,(65,(66,(67,68)))))),((69,(70,(71,72))),((73,(76,(75,74))),(77,78))))))))))))),(79,80)),(81,(82,(83,(85,(84,86))))))),(87,(88,89))))),(90,(((91,(92,(93,94))),(95,(96,97))),(98,99)))))))))),(100,101))))))));

TREE tnt_79 = (1,(2,(3,(4,(5,(6,((7,((8,9),(10,(11,(12,((13,(14,((15,(((((16,(17,18)),(((19,((20,21),(22,(23,24)))),(25,26)),(27,(28,(29,30))))),(31,((33,(34,35)),((32,(36,(37,(38,39)))),((40,((41,42),(43,44))),(45,((46,(49,(48,47))),((50,(51,(52,((53,(54,(55,(56,(57,58))))),(59,60))))),((61,((62,63),(64,((65,66),(67,68))))),((69,(70,(71,72))),(78,(77,(74,(75,(73,76))))))))))))))),(79,80)),(81,(82,(83,(85,(84,86))))))),(87,(88,89))))),(90,(((91,(92,(93,94))),(95,(96,97))),(98,99))))))))),(100,101))))))));

TREE tnt_80 = (1,(2,(3,(4,(5,(6,((7,(10,(8,(9,(11,(12,((13,(14,((15,(((((16,(17,18)),(((19,((20,21),(22,(23,24)))),(25,26)),(27,(28,(29,30))))),(31,((33,(34,35)),((32,(36,(37,(38,39)))),((40,((41,42),(43,44))),(45,((46,(49,(48,47))),((50,(51,(52,((53,(54,(55,(56,(57,58))))),(59,60))))),((61,((62,63),(64,((65,66),(67,68))))),((69,(70,(71,72))),(78,(77,(74,(75,(73,76))))))))))))))),(79,80)),(81,(82,(83,(85,(84,86))))))),(87,(88,89))))),(90,(((91,(92,(93,94))),(95,(96,97))),(98,99)))))))))),(100,101))))))));

TREE tnt_81 = (1,(2,(3,(4,(5,(6,((7,((9,(8,10)),(11,(12,((13,(14,((15,(((((16,(17,18)),(((19,((20,21),(22,(23,24)))),(25,26)),(27,(28,(29,30))))),(31,((33,(34,35)),((32,(36,(37,(38,39)))),((40,((41,42),(43,44))),(45,((46,(49,(48,47))),((50,(51,(52,((53,(54,((56,55),(57,58)))),(59,60))))),((61,((62,63),(64,((65,66),(67,68))))),((69,(70,(71,72))),(((75,76),(73,74)),(77,78)))))))))))),(79,80)),(81,(82,(83,(85,(84,86))))))),(87,(88,89))))),(90,(((91,(92,(93,94))),(95,(96,97))),(98,99)))))))),(100,101))))))));

TREE tnt_82 = (1,(2,(3,(4,(5,(6,((7,((9,(8,10)),(11,(12,((13,(14,((15,(((((16,(17,18)),(((19,((20,21),(22,(23,24)))),(25,26)),(27,(28,(29,30))))),(31,(32,((33,(34,35)),((36,(37,(38,39))),((40,((41,42),(43,44))),(45,((46,(49,(48,47))),((50,(51,(52,((53,(54,((56,55),(57,58)))),(59,60))))),((61,((62,63),(64,((65,66),(67,68))))),((69,(70,(71,72))),(((75,76),(73,74)),(77,78))))))))))))),(79,80)),(81,(82,(83,(85,(84,86))))))),(87,(88,89))))),(90,(((91,(92,(93,94))),(95,(96,97))),(98,99)))))))),(100,101))))))));

TREE tnt_83 = (1,(2,(3,(4,(5,(6,((7,((9,(8,10)),(11,(12,((13,(14,((15,(((((16,(17,18)),(((19,((20,21),(22,(23,24)))),(25,26)),(27,(28,(29,30))))),(31,((33,(34,35)),((32,(36,(37,(38,39)))),((40,((41,42),(43,44))),(45,((46,(49,(48,47))),((50,(51,(52,((53,(54,((56,55),(57,58)))),(59,60))))),((61,((62,63),(64,(65,(66,(67,68)))))),((69,(70,(71,72))),(((75,76),(73,74)),(77,78)))))))))))),(79,80)),(81,(82,(83,(85,(84,86))))))),(87,(88,89))))),(90,(((91,(92,(93,94))),(95,(96,97))),(98,99)))))))),(100,101))))))));

TREE tnt_84 = (1,(2,(3,(4,(5,(6,((7,((9,(8,10)),(11,(12,((13,(14,((15,(((((16,(17,18)),(((19,((20,21),(22,(23,24)))),(25,26)),(27,(28,(29,30))))),(31,((33,(34,35)),((32,(36,(37,(38,39)))),((40,((41,42),(43,44))),(45,((46,(49,(48,47))),((50,(51,(52,((53,(54,((56,55),(57,58)))),(59,60))))),((61,((62,63),(64,((65,66),(67,68))))),((69,(70,(71,72))),((73,(74,(75,76))),(77,78)))))))))))),(79,80)),(81,(82,(83,(85,(84,86))))))),(87,(88,89))))),(90,(((91,(92,(93,94))),(95,(96,97))),(98,99)))))))),(100,101))))))));

TREE tnt_85 = (1,(2,(3,(4,(5,(6,((7,((8,10),(9,(11,(12,((13,(14,((15,(((((16,(17,18)),(((19,((20,21),(22,(23,24)))),(25,26)),(27,(28,(29,30))))),(31,((33,(34,35)),((32,(36,(37,(38,39)))),((40,((41,42),(43,44))),(45,((46,(49,(48,47))),((50,(51,(52,((53,(54,((56,55),(57,58)))),(59,60))))),((61,((62,63),(64,((65,66),(67,68))))),((69,(70,(71,72))),(((75,76),(73,74)),(77,78)))))))))))),(79,80)),(81,(82,(83,(85,(84,86))))))),(87,(88,89))))),(90,(((91,(92,(93,94))),(95,(96,97))),(98,99))))))))),(100,101))))))));

TREE tnt_86 = (1,(2,(3,(4,(5,(6,((7,((9,(8,10)),(11,(12,((13,(14,((15,(((((16,(17,18)),(((19,((20,21),(22,(23,24)))),(25,26)),(27,(28,(29,30))))),(31,((33,(34,35)),((32,(36,(37,(38,39)))),((40,((41,42),(43,44))),(45,((46,(49,(48,47))),((50,(51,(52,((53,(54,((56,55),(57,58)))),(59,60))))),((61,((62,63),(64,((65,66),(67,68))))),((69,(70,(71,72))),(78,(77,(74,(73,(75,76))))))))))))))),(79,80)),(81,(82,(83,(85,(84,86))))))),(87,(88,89))))),(90,(((91,(92,(93,94))),(95,(96,97))),(98,99)))))))),(100,101))))))));

TREE tnt_87 = (1,(2,(3,(4,(5,(6,((7,(10,((8,9),(11,(12,((13,(14,((15,(((((16,(17,18)),(((19,((20,21),(22,(23,24)))),(25,26)),(27,(28,(29,30))))),(31,((33,(34,35)),((32,(36,(37,(38,39)))),((40,((41,42),(43,44))),(45,((46,(49,(48,47))),((50,(51,(52,((53,(54,((56,55),(57,58)))),(59,60))))),((61,((62,63),(64,((65,66),(67,68))))),((69,(70,(71,72))),(((75,76),(73,74)),(77,78)))))))))))),(79,80)),(81,(82,(83,(85,(84,86))))))),(87,(88,89))))),(90,(((91,(92,(93,94))),(95,(96,97))),(98,99))))))))),(100,101))))))));

TREE tnt_88 = (1,(2,(3,(4,(5,(6,((7,((10,(8,9)),(11,(12,((13,(14,((15,(((((16,(17,18)),(((19,((20,21),(22,(23,24)))),(25,26)),(27,(28,(29,30))))),(31,((33,(34,35)),((32,(36,(37,(38,39)))),((40,((41,42),(43,44))),(45,((46,(49,(48,47))),((50,(51,(52,((53,(54,((56,55),(57,58)))),(59,60))))),((61,((62,63),(64,((65,66),(67,68))))),((69,(70,(71,72))),(((75,76),(73,74)),(77,78)))))))))))),(79,80)),(81,(82,(83,(85,(84,86))))))),(87,(88,89))))),(90,(((91,(92,(93,94))),(95,(96,97))),(98,99)))))))),(100,101))))))));

TREE tnt_89 = (1,(2,(3,(4,(5,(6,((7,(8,((9,10),(11,(12,((13,(14,((15,(((((16,(17,18)),(((19,((20,21),(22,(23,24)))),(25,26)),(27,(28,(29,30))))),(31,(32,((33,(34,35)),((36,(37,(38,39))),((40,((41,42),(43,44))),(45,((46,(49,(48,47))),((50,(51,(52,((53,(54,((56,55),(57,58)))),(59,60))))),((61,((62,63),(64,((65,66),(67,68))))),((69,(70,(71,72))),(((75,76),(73,74)),(77,78))))))))))))),(79,80)),(81,(82,(83,(85,(84,86))))))),(87,(88,89))))),(90,(((91,(92,(93,94))),(95,(96,97))),(98,99))))))))),(100,101))))))));

TREE tnt_90 = (1,(2,(3,(4,(5,(6,((7,(10,((8,9),(11,(12,((13,(14,((15,(((((16,(17,18)),(((19,((20,21),(22,(23,24)))),(25,26)),(27,(28,(29,30))))),(31,(32,((33,(34,35)),((36,(37,(38,39))),((40,((41,42),(43,44))),(45,((46,(49,(48,47))),((50,(51,(52,((53,(54,((56,55),(57,58)))),(59,60))))),((61,((62,63),(64,((65,66),(67,68))))),((69,(70,(71,72))),(((75,76),(73,74)),(77,78))))))))))))),(79,80)),(81,(82,(83,(85,(84,86))))))),(87,(88,89))))),(90,(((91,(92,(93,94))),(95,(96,97))),(98,99))))))))),(100,101))))))));

TREE tnt_91 = (1,(2,(3,(4,(5,(6,((7,((9,(8,10)),(11,(12,((13,(14,((15,(((((16,(17,18)),(((19,((20,21),(22,(23,24)))),(25,26)),(27,(28,(29,30))))),(31,((33,(34,35)),((32,(36,(37,(38,39)))),((40,((41,42),(43,44))),(45,((46,(48,(49,47))),((50,(51,(52,((53,(54,((56,55),(57,58)))),(59,60))))),((61,((62,63),(64,((65,66),(67,68))))),((69,(70,(71,72))),((73,(74,(75,76))),(77,78)))))))))))),(79,80)),(81,(82,(83,(84,(85,86))))))),(87,(88,89))))),(90,(((91,(92,(93,94))),(95,(96,97))),(98,99)))))))),(100,101))))))));

TREE tnt_92 = (1,(2,(3,(4,(5,(6,((7,(10,((8,9),(11,(12,((13,(14,((15,(((((16,(17,18)),(((19,((20,21),(22,(23,24)))),(25,26)),(27,(28,(29,30))))),(31,((33,(34,35)),((32,(36,(37,(38,39)))),((40,((41,42),(43,44))),(45,((46,(48,(49,47))),((50,(51,(52,((53,(54,((56,55),(57,58)))),(59,60))))),((61,((62,63),(64,((65,66),(67,68))))),((69,(70,(71,72))),((73,(74,(75,76))),(77,78)))))))))))),(79,80)),(81,(82,(83,(84,(85,86))))))),(87,(88,89))))),(90,(((91,(92,(93,94))),(95,(96,97))),(98,99))))))))),(100,101))))))));

TREE tnt_93 = (1,(2,(3,(4,(5,(6,((7,(10,((8,9),(11,(12,((13,(14,((15,(((((16,(17,18)),(((19,((20,21),(22,(23,24)))),(25,26)),(27,(28,(29,30))))),(31,((33,(34,35)),((32,(36,(37,(38,39)))),((40,((41,42),(43,44))),(45,((46,(48,(49,47))),((50,(51,(52,((53,(54,((56,55),(57,58)))),(59,60))))),((61,((62,63),(64,((65,66),(67,68))))),((69,(70,(71,72))),((73,(74,(75,76))),(77,78)))))))))))),(79,80)),(81,(82,(83,(85,(84,86))))))),(87,(88,89))))),(90,(((91,(92,(93,94))),(95,(96,97))),(98,99))))))))),(100,101))))))));

TREE tnt_94 = (1,(2,(3,(4,(5,(6,((7,(10,(8,(9,(11,(12,((13,(14,((15,(((((16,(17,18)),(((19,((20,21),(22,(23,24)))),(25,26)),(27,(28,(29,30))))),(31,((33,(34,35)),((32,(36,(37,(38,39)))),((40,((41,42),(43,44))),(45,((46,(48,(49,47))),((50,(51,(52,((53,(54,((56,55),(57,58)))),(59,60))))),((61,((62,63),(64,((65,66),(67,68))))),((69,(70,(71,72))),((73,(74,(75,76))),(77,78)))))))))))),(79,80)),(81,(82,(83,(85,(84,86))))))),(87,(88,89))))),(90,(((91,(92,(93,94))),(95,(96,97))),(98,99)))))))))),(100,101))))))));

TREE tnt_95 = (1,(2,(3,(4,(5,(6,((7,(8,((9,10),(11,(12,((13,(14,((15,(((((16,(17,18)),(((19,((20,21),(22,(23,24)))),(25,26)),(27,(28,(29,30))))),(31,((33,(34,35)),((32,(36,(37,(38,39)))),((40,((41,42),(43,44))),(45,((46,(48,(49,47))),((50,(51,(52,((53,(54,((56,55),(57,58)))),(59,60))))),((61,((62,63),(64,((65,66),(67,68))))),((69,(70,(71,72))),((73,(74,(75,76))),(77,78)))))))))))),(79,80)),(81,(82,(83,(85,(84,86))))))),(87,(88,89))))),(90,(((91,(92,(93,94))),(95,(96,97))),(98,99))))))))),(100,101))))))));

TREE tnt_96 = (1,(2,(3,(4,(5,(6,((7,(10,(8,(9,(11,(12,((13,(14,((15,(((((16,(17,18)),(((19,((20,21),(22,(23,24)))),(25,26)),(27,(28,(29,30))))),(31,((33,(34,35)),((32,(36,(37,(38,39)))),((40,((41,42),(43,44))),(45,((46,(48,(49,47))),((50,(51,(52,((53,(54,((56,55),(57,58)))),(59,60))))),((61,((62,63),(64,(65,(66,(67,68)))))),((69,(70,(71,72))),((73,(74,(75,76))),(77,78)))))))))))),(79,80)),(81,(82,(83,(85,(84,86))))))),(87,(88,89))))),(90,(((91,(92,(93,94))),(95,(96,97))),(98,99)))))))))),(100,101))))))));

TREE tnt_97 = (1,(2,(3,(4,(5,(6,((7,(8,((9,10),(11,(12,((13,(14,((15,(((((16,(17,18)),(((19,((20,21),(22,(23,24)))),(25,26)),(27,(28,(29,30))))),(31,(32,((33,(34,35)),((36,(37,(38,39))),((40,((41,42),(43,44))),(45,((46,(48,(49,47))),((50,(51,(52,((53,(54,((56,55),(57,58)))),(59,60))))),((61,((62,63),(64,((65,66),(67,68))))),((69,(70,(71,72))),((73,(74,(75,76))),(77,78))))))))))))),(79,80)),(81,(82,(83,(85,(84,86))))))),(87,(88,89))))),(90,(((91,(92,(93,94))),(95,(96,97))),(98,99))))))))),(100,101))))))));

TREE tnt_98 = (1,(2,(3,(4,(5,(6,((7,((8,(9,10)),(11,(12,((13,(14,((15,(((((16,(17,18)),(((19,((20,21),(22,(23,24)))),(25,26)),(27,(28,(29,30))))),(31,(32,((33,(34,35)),((36,(37,(38,39))),((40,((41,42),(43,44))),(45,((46,(48,(49,47))),((50,(51,(52,((53,(54,((56,55),(57,58)))),(59,60))))),((61,((62,63),(64,((65,66),(67,68))))),((69,(70,(71,72))),((73,(74,(75,76))),(77,78))))))))))))),(79,80)),(81,(82,(83,(85,(84,86))))))),(87,(88,89))))),(90,(((91,(92,(93,94))),(95,(96,97))),(98,99)))))))),(100,101))))))));

TREE tnt_99 = (1,(2,(3,(4,(5,(6,((7,((8,(9,10)),(11,(12,((13,(14,((15,(((((16,(17,18)),(((19,((20,21),(22,(23,24)))),(25,26)),(27,(28,(29,30))))),(31,(32,((33,(34,35)),((36,(37,(38,39))),((40,((41,42),(43,44))),(45,((46,(48,(49,47))),((50,(51,(52,((53,(54,((56,55),(57,58)))),(59,60))))),((61,((62,63),(64,((65,66),(67,68))))),((69,(70,(71,72))),(78,(77,(74,(73,(75,76)))))))))))))))),(79,80)),(81,(82,(83,(85,(84,86))))))),(87,(88,89))))),(90,(((91,(92,(93,94))),(95,(96,97))),(98,99)))))))),(100,101))))))));

TREE tnt_100 = (1,(2,(3,(4,(5,(6,((7,((8,10),(9,(11,(12,((13,(14,((15,(((((16,(17,18)),(((19,((20,21),(22,(23,24)))),(25,26)),(27,(28,(29,30))))),(31,(32,((33,(34,35)),((36,(37,(38,39))),((40,((41,42),(43,44))),(45,((46,(48,(49,47))),((50,(51,(52,((53,(54,((56,55),(57,58)))),(59,60))))),((61,((62,63),(64,((65,66),(67,68))))),((69,(70,(71,72))),(78,(77,(74,(73,(75,76)))))))))))))))),(79,80)),(81,(82,(83,(85,(84,86))))))),(87,(88,89))))),(90,(((91,(92,(93,94))),(95,(96,97))),(98,99))))))))),(100,101))))))));

TREE tnt_101 = (1,(2,(3,(4,(5,(6,((7,(10,((8,9),(11,(12,((13,(14,((15,(((((16,(17,18)),(((19,((20,21),(22,(23,24)))),(25,26)),(27,(28,(29,30))))),(31,((33,(34,35)),(((32,36),(37,(38,39))),((40,((41,42),(43,44))),(45,((46,(47,(48,49))),((50,(52,(51,((53,(54,(55,(56,(57,58))))),(59,60))))),((61,((62,63),(64,(65,(66,(67,68)))))),((69,(70,(71,72))),(78,(77,(74,(75,(73,76))))))))))))))),(79,80)),(81,(82,(83,(84,(85,86))))))),(87,(88,89))))),(90,(((91,(92,(93,94))),(95,(96,97))),(98,99))))))))),(100,101))))))));

TREE tnt_102 = (1,(2,(3,(4,(5,(6,((7,((8,(9,10)),(11,(12,((13,(14,((15,(((((16,(17,18)),(((19,((20,21),(22,(23,24)))),(25,26)),(27,(28,(29,30))))),(31,((33,(34,35)),((32,(36,(37,(38,39)))),((40,((41,42),(43,44))),(45,((46,(47,(48,49))),((50,(51,(52,((53,(54,(55,(56,(57,58))))),(59,60))))),((61,((62,63),(64,(65,(66,(67,68)))))),((69,(70,(71,72))),((73,(74,(75,76))),(77,78)))))))))))),(79,80)),(81,(82,(83,(85,(84,86))))))),(87,(88,89))))),(90,(((91,(92,(93,94))),(95,(96,97))),(98,99)))))))),(100,101))))))));

TREE tnt_103 = (1,(2,(3,(4,(5,(6,((7,((8,(9,10)),(11,(12,((13,(14,((15,(((((16,(17,18)),(((19,((20,21),(22,(23,24)))),(25,26)),(27,(28,(29,30))))),(31,(32,((33,(34,35)),((36,(37,(38,39))),((40,((41,42),(43,44))),(45,((46,(47,(48,49))),((50,(51,(52,((53,(54,(55,(56,(57,58))))),(59,60))))),((61,((62,63),(64,(65,(66,(67,68)))))),((69,(70,(71,72))),(((75,76),(73,74)),(77,78))))))))))))),(79,80)),(81,(82,(83,(85,(84,86))))))),(87,(88,89))))),(90,(((91,(92,(93,94))),(95,(96,97))),(98,99)))))))),(100,101))))))));

TREE tnt_104 = (1,(2,(3,(4,(5,(6,((7,((8,(9,10)),(11,(12,((13,(14,((15,(((((16,(17,18)),(((19,((20,21),(22,(23,24)))),(25,26)),(27,(28,(29,30))))),(31,((33,(34,35)),((32,(36,(37,(38,39)))),((40,((41,42),(43,44))),(45,((46,(47,(48,49))),((50,(51,(52,((53,(54,(55,(56,(57,58))))),(59,60))))),((61,((62,63),(64,(65,(66,(67,68)))))),((69,(70,(71,72))),(((75,76),(73,74)),(77,78)))))))))))),(79,80)),(81,(82,(83,(84,(85,86))))))),(87,(88,89))))),(90,(((91,(92,(93,94))),(95,(96,97))),(98,99)))))))),(100,101))))))));

TREE tnt_105 = (1,(2,(3,(4,(5,(6,((7,((8,(9,10)),(11,(12,((13,(14,((15,(((((16,(17,18)),(((19,((20,21),(22,(23,24)))),(25,26)),(27,(28,(29,30))))),(31,(32,((33,(34,35)),((36,(37,(38,39))),((40,((41,42),(43,44))),(45,((46,(47,(48,49))),((50,(51,(52,((53,(54,(55,(56,(57,58))))),(59,60))))),((61,((62,63),(64,((65,66),(67,68))))),((69,(70,(71,72))),(((75,76),(73,74)),(77,78))))))))))))),(79,80)),(81,(82,(83,(84,(85,86))))))),(87,(88,89))))),(90,(((91,(92,(93,94))),(95,(96,97))),(98,99)))))))),(100,101))))))));

TREE tnt_106 = (1,(2,(3,(4,(5,(6,((7,((8,(9,10)),(11,(12,((13,(14,((15,(((((16,(17,18)),(((19,((20,21),(22,(23,24)))),(25,26)),(27,(28,(29,30))))),(31,(32,((33,(34,35)),((36,(37,(38,39))),((40,((41,42),(43,44))),(45,((46,(47,(48,49))),((50,(51,(52,((53,(54,(55,(56,(57,58))))),(59,60))))),((61,((62,63),(64,((65,66),(67,68))))),((69,(70,(71,72))),(78,(77,(74,(73,(75,76)))))))))))))))),(79,80)),(81,(82,(83,(84,(85,86))))))),(87,(88,89))))),(90,(((91,(92,(93,94))),(95,(96,97))),(98,99)))))))),(100,101))))))));

TREE tnt_107 = (1,(2,(3,(4,(5,(6,((7,(8,((9,10),(11,(12,((13,(14,((15,(((((16,(17,18)),(((19,((20,21),(22,(23,24)))),(25,26)),(27,(28,(29,30))))),(31,(32,((33,(34,35)),((36,(37,(38,39))),((40,((41,42),(43,44))),(45,((46,(47,(48,49))),((50,(51,(52,((53,(54,(55,(56,(57,58))))),(59,60))))),((61,((62,63),(64,(65,(66,(67,68)))))),((69,(70,(71,72))),(((75,76),(73,74)),(77,78))))))))))))),(79,80)),(81,(82,(83,(84,(85,86))))))),(87,(88,89))))),(90,(((91,(92,(93,94))),(95,(96,97))),(98,99))))))))),(100,101))))))));

TREE tnt_108 = (1,(2,(3,(4,(5,(6,((7,(10,((8,9),(11,(12,((13,(14,((15,(((((16,(17,18)),(((19,((20,21),(22,(23,24)))),(25,26)),(27,(28,(29,30))))),(31,(32,((33,(34,35)),((36,(37,(38,39))),((40,((41,42),(43,44))),(45,((46,(47,(48,49))),((50,(51,(52,((53,(54,(55,(56,(57,58))))),(59,60))))),((61,((62,63),(64,(65,(66,(67,68)))))),((69,(70,(71,72))),(((75,76),(73,74)),(77,78))))))))))))),(79,80)),(81,(82,(83,(84,(85,86))))))),(87,(88,89))))),(90,(((91,(92,(93,94))),(95,(96,97))),(98,99))))))))),(100,101))))))));

TREE tnt_109 = (1,(2,(3,(4,(5,(6,((7,((10,(8,9)),(11,(12,((13,(14,((15,(((((16,(17,18)),(((19,((20,21),(22,(23,24)))),(25,26)),(27,(28,(29,30))))),(31,(32,((33,(34,35)),((36,(37,(38,39))),((40,((41,42),(43,44))),(45,((46,(47,(48,49))),((50,(51,(52,((53,(54,(55,(56,(57,58))))),(59,60))))),((61,((62,63),(64,(65,(66,(67,68)))))),((69,(70,(71,72))),(((75,76),(73,74)),(77,78))))))))))))),(79,80)),(81,(82,(83,(84,(85,86))))))),(87,(88,89))))),(90,(((91,(92,(93,94))),(95,(96,97))),(98,99)))))))),(100,101))))))));

TREE tnt_110 = (1,(2,(3,(4,(5,(6,((7,(10,((8,9),(11,(12,((13,(14,((15,(((((16,(17,18)),(((19,((20,21),(22,(23,24)))),(25,26)),(27,(28,(29,30))))),(31,((33,(34,35)),(((32,36),(37,(38,39))),((40,((41,42),(43,44))),(45,((46,(48,(49,47))),((50,(((53,(54,(56,(55,(57,58))))),(59,60)),(51,52))),((61,((62,63),(64,(65,(66,(67,68)))))),((69,(70,(71,72))),((75,(76,(73,74))),(77,78)))))))))))),(79,80)),(81,(82,(83,(85,(84,86))))))),(87,(88,89))))),(90,(((91,(92,(93,94))),(95,(96,97))),(98,99))))))))),(100,101))))))));

TREE tnt_111 = (1,(2,(3,(4,(5,(6,((7,(8,((9,10),(11,(12,((13,(14,((15,(((((16,(17,18)),(((19,((20,21),(22,(23,24)))),(25,26)),(27,(28,(29,30))))),(31,((33,(34,35)),(((32,36),(37,(38,39))),((40,((41,42),(43,44))),(45,((46,(48,(49,47))),((50,(((53,(54,(56,(55,(57,58))))),(59,60)),(51,52))),((61,((62,63),(64,(65,(66,(67,68)))))),((69,(70,(71,72))),((75,(76,(73,74))),(77,78)))))))))))),(79,80)),(81,(82,(83,(85,(84,86))))))),(87,(88,89))))),(90,(((91,(92,(93,94))),(95,(96,97))),(98,99))))))))),(100,101))))))));

TREE tnt_112 = (1,(2,(3,(4,(5,(6,((7,(10,(8,(9,(11,(12,((13,(14,((15,(((((16,(17,18)),(((19,((20,21),(22,(23,24)))),(25,26)),(27,(28,(29,30))))),(31,(32,((33,(34,35)),((36,(37,(38,39))),((40,((41,42),(43,44))),(45,((46,(48,(49,47))),((50,(((53,(54,(56,(55,(57,58))))),(59,60)),(51,52))),((61,((62,63),(64,(65,(66,(67,68)))))),((69,(70,(71,72))),((75,(76,(73,74))),(77,78))))))))))))),(79,80)),(81,(82,(83,(85,(84,86))))))),(87,(88,89))))),(90,(((91,(92,(93,94))),(95,(96,97))),(98,99)))))))))),(100,101))))))));

TREE tnt_113 = (1,(2,(3,(4,(5,(6,((7,(8,(9,(10,(11,(12,((13,(14,((15,(((((16,(17,18)),(((19,((20,21),(22,(23,24)))),(25,26)),(27,(28,(29,30))))),(31,(32,((33,(34,35)),((36,(37,(38,39))),((40,((41,42),(43,44))),(45,((46,(49,(48,47))),((50,(52,(51,((53,(54,((56,55),(57,58)))),(59,60))))),((61,((62,63),(64,((65,66),(67,68))))),((69,(70,(71,72))),(78,(77,(74,(73,(75,76)))))))))))))))),(79,80)),(81,(82,(83,(84,(85,86))))))),(87,(88,89))))),(90,(((91,(92,(93,94))),(95,(96,97))),(98,99)))))))))),(100,101))))))));

TREE tnt_114 = (1,(2,(3,(4,(5,(6,((7,((8,9),(10,(11,(12,((13,(14,((15,(((((16,(17,18)),(((19,((20,21),(22,(23,24)))),(25,26)),(27,(28,(29,30))))),(31,(32,((33,(34,35)),((36,(37,(38,39))),((40,((41,42),(43,44))),(45,((46,(49,(48,47))),((50,(52,(51,((53,(54,((56,55),(57,58)))),(59,60))))),((61,((62,63),(64,((65,66),(67,68))))),((69,(70,(71,72))),((76,(75,(73,74))),(77,78))))))))))))),(79,80)),(81,(82,(83,(84,(85,86))))))),(87,(88,89))))),(90,(((91,(92,(93,94))),(95,(96,97))),(98,99))))))))),(100,101))))))));

TREE tnt_115 = (1,(2,(3,(4,(5,(6,((7,(8,(10,(9,(11,(12,((13,(14,((15,(((((16,(17,18)),(((19,((20,21),(22,(23,24)))),(25,26)),(27,(28,(29,30))))),(31,(32,((33,(34,35)),((36,(37,(38,39))),((40,((41,42),(43,44))),(45,((46,(49,(48,47))),((50,(52,(51,((53,(54,((56,55),(57,58)))),(59,60))))),((61,((62,63),(64,((65,66),(67,68))))),((69,(70,(71,72))),((76,(75,(73,74))),(77,78))))))))))))),(79,80)),(81,(82,(83,(84,(85,86))))))),(87,(88,89))))),(90,(((91,(92,(93,94))),(95,(96,97))),(98,99)))))))))),(100,101))))))));

TREE tnt_116 = (1,(2,(3,(4,(5,(6,((7,((8,9),(10,(11,(12,((13,(14,((15,(((((16,(17,18)),(((19,((20,21),(22,(23,24)))),(25,26)),(27,(28,(29,30))))),(31,(32,((33,(34,35)),((36,(37,(38,39))),((40,((41,42),(43,44))),(45,((46,(49,(48,47))),((50,(52,(51,((53,(54,((56,55),(57,58)))),(59,60))))),((61,((62,63),(64,((65,66),(67,68))))),((69,(70,(71,72))),(78,(77,(74,(73,(75,76)))))))))))))))),(79,80)),(81,(82,(83,(84,(85,86))))))),(87,(88,89))))),(90,(((91,(92,(93,94))),(95,(96,97))),(98,99))))))))),(100,101))))))));

TREE tnt_117 = (1,(2,(3,(4,(5,(6,((7,(10,(8,(9,(11,(12,((13,(14,((15,(((((16,(17,18)),(((19,((20,21),(22,(23,24)))),(25,26)),(27,(28,(29,30))))),(31,(32,((33,(34,35)),((36,(37,(38,39))),((40,((41,42),(43,44))),(45,((46,(48,(49,47))),((50,(52,(51,((53,(54,((56,55),(57,58)))),(59,60))))),((61,((62,63),(64,((65,66),(67,68))))),((69,(70,(71,72))),((76,(75,(73,74))),(77,78))))))))))))),(79,80)),(81,(82,(83,(85,(84,86))))))),(87,(88,89))))),(90,(((91,(92,(93,94))),(95,(96,97))),(98,99)))))))))),(100,101))))))));

TREE tnt_118 = (1,(2,(3,(4,(5,(6,((7,(8,(10,(9,(11,(12,((13,(14,((15,(((((16,(17,18)),(((19,((20,21),(22,(23,24)))),(25,26)),(27,(28,(29,30))))),(31,(32,((33,(34,35)),((36,(37,(38,39))),((40,((41,42),(43,44))),(45,((46,(48,(49,47))),((50,(52,(51,((53,(54,((56,55),(57,58)))),(59,60))))),((61,((62,63),(64,((65,66),(67,68))))),((69,(70,(71,72))),((76,(75,(73,74))),(77,78))))))))))))),(79,80)),(81,(82,(83,(85,(84,86))))))),(87,(88,89))))),(90,(((91,(92,(93,94))),(95,(96,97))),(98,99)))))))))),(100,101))))))));

TREE tnt_119 = (1,(2,(3,(4,(5,(6,((7,(8,(10,(9,(11,(12,((13,(14,((15,(((((16,(17,18)),(((19,((20,21),(22,(23,24)))),(25,26)),(27,(28,(29,30))))),(31,(32,((33,(34,35)),((36,(37,(38,39))),((40,((41,42),(43,44))),(45,((46,(48,(49,47))),((50,(52,(51,((53,(54,((56,55),(57,58)))),(59,60))))),((61,((62,63),(64,((65,66),(67,68))))),((69,(70,(71,72))),(78,(77,(74,(75,(73,76)))))))))))))))),(79,80)),(81,(82,(83,(85,(84,86))))))),(87,(88,89))))),(90,(((91,(92,(93,94))),(95,(96,97))),(98,99)))))))))),(100,101))))))));

TREE tnt_120 = (1,(2,(3,(4,(5,(6,((7,(8,(9,(10,(11,(12,((13,(14,((15,(((((16,(17,18)),(((19,((20,21),(22,(23,24)))),(25,26)),(27,(28,(29,30))))),(31,(32,((33,(34,35)),((36,(37,(38,39))),((40,((41,42),(43,44))),(45,((46,(49,(48,47))),((50,(51,(52,((53,(54,(55,(56,(57,58))))),(59,60))))),((61,((62,63),(64,(65,(66,(67,68)))))),((69,(70,(71,72))),((76,(75,(73,74))),(77,78))))))))))))),(79,80)),(81,(82,(83,(85,(84,86))))))),(87,(88,89))))),(90,(((91,(92,(93,94))),(95,(96,97))),(98,99)))))))))),(100,101))))))));

END;

BEGIN CODONS;

CODESET * UNTITLED = universal: 1- 244;

END;

BEGIN LABELS;

TAXAGROUPLABEL Meiolaniidae;

TAXAGROUPLABEL Chelidae;

TAXAGROUPLABEL Pelomedusoides;

TAXAGROUPLABEL Glyptopsidae;

TAXAGROUPLABEL Baenidae;

TAXAGROUPLABEL Plesiochelyidae;

TAXAGROUPLABEL Chelonioidea_sl;

TAXAGROUPLABEL Chelydridae_sl;

TAXAGROUPLABEL Testudinidae;

TAXAGROUPLABEL Emydidae;

TAXAGROUPLABEL Geoemydidae;

TAXAGROUPLABEL Kinosternidae_sl;

TAXAGROUPLABEL Adocidae;

TAXAGROUPLABEL Trionychidae;

TAXAGROUPLABEL Carettochelyidae_sl;

TAXAGROUPLABEL Xinjiangchelyidae;

TAXAGROUPLABEL 'Sinemydae-Macrobaenidae';

TAXAGROUPLABEL 'Sterli''s';

TAXAGROUPLABEL 'Joyce''s';

TAXAGROUPLABEL Gaffney_1996;

TAXAGROUPLABEL Sterli_and_de_la_Fuente;

TAXAGROUPLABEL outgroups;

TAXAGROUPLABEL Testudinata;

TAXAGROUPLABEL stem_turtles;

TAXAGROUPLABEL Pleurodira;

TAXAGROUPLABEL Cryptodira;

TAXAGROUPLABEL score!;

CHARGROUPLABEL 'Joyce (2007)';

CHARGROUPLABEL 'Bona and de la Fuente (2005)';

CHARGROUPLABEL 'Meylan and Gaffney (1989)';

CHARGROUPLABEL New;

CHARGROUPLABEL 'Kear and Lee (2006)';

CHARGROUPLABEL 'Gaffney (1996)';

CHARGROUPLABEL 'Gaffney (1992)';

CHARGROUPLABEL 'Meylan (1987)';

CHARGROUPLABEL 'Hirayama et al. (2001)';

CHARGROUPLABEL 'Hirayama et al. (2000)';

CHARGROUPLABEL 'Hutchison (1991)';

CHARGROUPLABEL 'Iverson (1991)';

CHARGROUPLABEL 'Gaffney et al. (2007)';

CHARGROUPLABEL 'Gaffney (1990)';

CHARGROUPLABEL 'Gaffney (1983)';

CHARGROUPLABEL skull;

CHARGROUPLABEL mandibule;

CHARGROUPLABEL postcranium;

END;

BEGIN SETS;

TAXPARTITION * UNTITLED (TAXA = Untitled_Taxa) = Meiolaniidae : 14- 17, Chelidae : 25- 32, Pelomedusoides : 33- 36, Glyptopsidae : 37- 39, Baenidae : 40- 45, Plesiochelyidae : 46- 49, Chelonioidea_sl : 50- 54, Chelydridae_sl : 55- 58, Testudinidae : 60- 64, Emydidae : 66- 68, Geoemydidae : 69, Kinosternidae_sl : 74- 77, Adocidae : 78- 80, Trionychidae : 82- 85, Carettochelyidae_sl : 87- 88, Xinjiangchelyidae : 93, 'Sinemydae-Macrobaenidae' : 94 96- 97 100;

TAXPARTITION phylogeny (TAXA = Untitled_Taxa) = 'Sterli''s' : 1- 3 10- 11 14 18 27- 29 31 64 67 113, 'Joyce''s' : 5- 9 17 22- 24 26 30 34- 54 56- 61 66 69 71- 73 75- 76 78 80- 82 85 88 93- 94 96- 97 100 106- 107, Gaffney_1996 : 15- 16, Sterli_and_de_la_Fuente : 20- 21;

TAXPARTITION Families (TAXA = Untitled_Taxa) = Meiolaniidae : 14- 17, Chelidae : 25- 32, Pelomedusoides : 33- 36, Glyptopsidae : 37- 39, Baenidae : 40- 45, Plesiochelyidae : 46- 49, Chelonioidea_sl : 50- 54, Chelydridae_sl : 55- 58, Testudinidae : 60- 61 63- 64, Emydidae : 66- 67, Geoemydidae : 69, Kinosternidae_sl : 74- 77, Adocidae : 78- 80, Trionychidae : 82- 85, Carettochelyidae_sl : 87- 88, Xinjiangchelyidae : 93, 'Sinemydae-Macrobaenidae' : 94 96- 97 100;

TAXPARTITION Testudinata (TAXA = Untitled_Taxa) = outgroups : 1- 3 113, Testudinata : 4- 12 14- 18 20- 61 63- 67 69- 85 87- 88 93- 94 96- 97 100 106- 107;

TAXPARTITION 'stem-pleuro-crypto' (TAXA = Untitled_Taxa) = stem_turtles : 4- 12 14- 17 20- 21 106- 107, Pleurodira : 22- 36, Cryptodira : 50- 61 63- 67 69- 85 87- 88 93- 94 96- 97 100;

TAXPARTITION Partition (TAXA = Untitled_Taxa) = Meiolaniidae : 14- 17, Chelidae : 25- 32, Pelomedusoides : 33- 36, Glyptopsidae : 37- 39, Baenidae : 40- 45, Plesiochelyidae : 46- 49, Chelonioidea_sl : 50- 54, Chelydridae_sl : 55- 58, Testudinidae : 60- 61 63- 64, Emydidae : 66- 67, Geoemydidae : 69, Kinosternidae_sl : 74- 77, Adocidae : 78- 80, Trionychidae : 82- 85, Carettochelyidae_sl : 87- 88, Xinjiangchelyidae : 93, 'Sinemydae-Macrobaenidae' : 94 96- 97 100;

TAXPARTITION 'exclude-score' (TAXA = Untitled_Taxa) = score! : 1- 4 12 25 33 63 65 113;

CHARPARTITION * UNTITLED = 'Joyce (2007)' : 1- 10 12- 16 20- 26 30- 36 41- 44 46- 52 55- 64 67- 68 70- 71 73- 78 80- 82 89 106- 111 114- 115 117 119 121- 123 126- 130 132- 134 137- 142 144-147\3 148 150- 157 159- 160 162 164- 166 169- 174 185- 190 193 197- 198 205 209- 212 215- 220, 'Bona and de la Fuente (2005)' : 11 54 184, 'Meylan and Gaffney (1989)' : 17 131, New : 18 28- 29 37- 39 45 65- 66 69-72\3 79 83- 85 87 98 102- 105 112- 113 116 118 120 124 135- 136 145 158-161\3 163 167 175- 177 180 182- 183 191- 192 195- 196 199-202\3 203- 204 207- 208 221, 'Kear and Lee (2006)' : 19, 'Gaffney (1996)' : 27 91- 92 94- 96 99- 101 181 194, 'Gaffney (1992)' : 40 93 97, 'Meylan (1987)' : 53 86 200 213- 214 222, 'Hirayama et al. (2001)' : 90 206, 'Hirayama et al. (2000)' : 125 143 179, 'Hutchison (1991)' : 146, 'Iverson (1991)' : 149 168, 'Gaffney et al. (2007)' : 178, 'Gaffney (1990)' : 201;

CHARPARTITION phylogeny = 'Joyce (2007)' : 1- 10 12- 16 20- 26 30- 36 41- 44 46- 52 55- 64 67- 68 70- 71 73- 78 80- 82 89 106- 111 114- 115 117 119 121- 123 126- 130 132- 134 137- 142 144-147\3 148 150- 157 159- 160 162 164- 166 169- 174 185- 190 193 197- 198 205 209- 212 215- 220, 'Bona and de la Fuente (2005)' : 11 54 184, 'Meylan and Gaffney (1989)' : 17 131, New : 18 28- 29 37- 39 45 65- 66 69-72\3 79 83- 85 87 98 102- 103 112- 113 116 118 120 124 135- 136 145 158-161\3 163 167 175- 177 180 182- 183 191- 192 195- 196 199-202\3 203- 204 207- 208 221, 'Kear and Lee (2006)' : 19, 'Gaffney (1996)' : 27 91- 92 94- 96 99- 101 181 194, 'Gaffney (1992)' : 40 93 97, 'Meylan (1987)' : 53 86 200 213- 214 222, 'Hirayama et al. (2001)' : 90 206, 'Hirayama et al. (2000)' : 125 143 179, 'Hutchison (1991)' : 146, 'Iverson (1991)' : 149 168, 'Gaffney et al. (2007)' : 178, 'Gaffney (1990)' : 201;

CHARPARTITION 'cranium-postcranium' = skull : 1- 103 106- 107, mandibule : 108, postcranium : 109- 222;

END;

BEGIN ASSUMPTIONS;

TYPESET * UNTITLED = unord: 1- 6 8- 18 20- 26 28- 38 40 42- 47 49 51- 56 58- 74 77- 81 83- 87 89- 108 111- 113 115- 118 120- 121 123- 124 128- 145 148 150- 163 165- 175 177- 197 200- 216 219- 230 232- 244, ord: 7 19 27 39 41 48 50 57 75- 76 82 88 109- 110 114 119-125\3 126- 127 146- 147 149 164 176 198- 199 217- 218 231;

EXSET * UNTITLED = ;

END;

BEGIN MESQUITECHARMODELS;

ProbModelSet * UNTITLED = 'Mk1 (est.)': 1- 143 145- 147 149- 153 155- 159 161- 164 166- 172 177- 181 186- 191 193 198- 199 206 215- 216 218 220- 244;

END;

Begin MESQUITE;

MESQUITESCRIPTVERSION 2;

TITLE AUTO;

tell ProjectCoordinator;

timeSaved 1526721063793;

getEmployee #mesquite.minimal.ManageTaxa.ManageTaxa;

tell It;

setID 0 5376187594245270751;

tell It;

setDefaultOrder 0 2 3 84 4 5 6 7 8 9 10 86 158 13 231 232 14 30 140 16 83 18 19 20 93 21 22 23 24 25 26 91 88 27 28 29 31 32 33 35 36 37 38 39 40 41 42 43 44 52 53 54 55 56 238 58 57 59 60 61 62 157 216 63 236 64 65 161 66 235 67 68 69 154 71 72 73 75 183 76 70 79 233 220 80 159 81 82 101 102 103 104 46 47 111 48 49 110 106 50 107 108 109 105 112 17 15 115 116 117 118 119 1 113;

attachments ;

endTell;

setID 1 6340754446151219931;

setID 2 8376767731966258259;

endTell;

getEmployee #mesquite.charMatrices.ManageCharacters.ManageCharacters;

tell It;

setID 0 1104077935316279450;

tell It;

setDefaultOrder 0 1 2 3 4 5 6 7 8 9 409 10 11 12 14 15 206 211 386 16 17 18 19 20 21 22 304 203 338 23 24 25 26 27 28 29 326 172 301 314 30 31 32 33 390 34 35 36 37 38 39 40 199 409 41 42 43 51 45 46 47 48 49 50 194 221 52 53 201 54 55 208 56 57 58 59 60 61 185 62 63 64 420 422 210 351 205 413 68 312 228 229 313 230 231 232 461 310 234 235 236 462 309 291 292 71 72 69 73 74 75 383 423 76 77 331 78 318 79 414 81 82 83 181 165 84 85 86 87 88 168 89 90 91 377 404 92 93 94 95 96 97 192 98 329 376 99 100 381 101 102 103 104 105 106 107 108 178 109 110 179 111 465 112 113 114 167 463 115 116 117 118 119 126 414 415 416 418 155 336 156 159 162 176 127 129 130 131 133 134 157 158 132 306 307 310 135 137 222 221 223 424 224 315 142 214 197 215 143 144 145 146 163 219 148 149 150 151 152 153 188 353 240 241 242 243 244 246 247 248 259 256 257 258 260 261 262 263 249 250 252 272 254 255;

attachments ;

endTell;

mqVersion 350;

checksumv 0 3 350518384 null getNumChars 244 numChars 244 getNumTaxa 114 numTaxa 114 short true bits 2305843009213693983 states 31 sumSquaresStatesOnly 55804.0 sumSquares -9.223372036854776E19 longCompressibleToShort false usingShortMatrix true NumFiles 1 NumMatrices 1;

mqVersion;

endTell;

getWindow;

tell It;

suppress;

setResourcesState false false 7;

setPopoutState 400;

setExplanationSize 0;

setAnnotationSize 0;

setFontIncAnnot 0;

setFontIncExp 0;

setSize 1920 961;

setLocation -8 0;

setFont SanSerif;

setFontSize 10;

getToolPalette;

tell It;

endTell;

desuppress;

endTell;

getEmployee #mesquite.minimal.ManageTaxa.ManageTaxa;

tell It;

showTaxa #5376187594245270751 #mesquite.lists.TaxonList.TaxonList;

tell It;

setTaxa #5376187594245270751;

getWindow;

tell It;

useTargetValue off;

setTargetValue ;

newAssistant #mesquite.lists.TaxonListCurrPartition.TaxonListCurrPartition;

newAssistant #mesquite.lists.DefaultTaxaOrder.DefaultTaxaOrder;

getTable;

tell It;

columnWidth 1 161;

endTell;

setExplanationSize 30;

setAnnotationSize 20;

setFontIncAnnot 0;

setFontIncExp 0;

setSize 1913 889;

setLocation -8 0;

setFont SanSerif;

setFontSize 10;

getToolPalette;

tell It;

setTool mesquite.lists.TaxonList.TaxonListWindow.arrow;

endTell;

endTell;

showWindow;

getEmployee #mesquite.lists.ColorTaxon.ColorTaxon;

tell It;

setColor Red;

removeColor off;

endTell;

getEmployee #mesquite.lists.TaxonListAnnotPanel.TaxonListAnnotPanel;

tell It;

togglePanel off;

endTell;

endTell;

endTell;

getEmployee #mesquite.trees.ManageTrees.ManageTrees;

tell It;

getTreeBlock 0;

tell It;

setSelected 1;

attachments ;

endTell;

showTrees 0 #mesquite.lists.TreesList.TreesList;

tell It;

setTreeBlock 1;

getWindow;

tell It;

useTargetValue off;

setTargetValue ;

newAssistant #mesquite.lists.NumForTreeList.NumForTreeList;

tell It;

suppress;

setValueTask #mesquite.trees.NumberOfTaxa.NumberOfTaxa;

desuppress;

endTell;

newAssistant #mesquite.lists.TreeListRooted.TreeListRooted;

newAssistant #mesquite.lists.TreeListPolys.TreeListPolys;

newAssistant #mesquite.lists.TreeListPolyAssumption.TreeListPolyAssumption;

setExplanationSize 30;

setAnnotationSize 20;

setFontIncAnnot 0;

setFontIncExp 0;

setSize 1913 889;

setLocation -8 0;

setFont SanSerif;

setFontSize 10;

getToolPalette;

tell It;

setTool mesquite.lists.TreesList.TreesListWindow.ibeam;

endTell;

endTell;

showWindow;

endTell;

endTell;

getEmployee #mesquite.trees.BasicTreeWindowCoord.BasicTreeWindowCoord;

tell It;

makeTreeWindow #8376767731966258259 #mesquite.trees.BasicTreeWindowMaker.BasicTreeWindowMaker;

tell It;

suppressEPCResponse;

setTreeSource #mesquite.trees.StoredTrees.StoredTrees;

tell It;

setTaxa #8376767731966258259;

setTreeBlock 1;

setTreeBlockID 01624961b33c1;

toggleUseWeights off;

endTell;

setAssignedID 730.1310057232862.1821540146855595573;

getTreeWindow;

tell It;

setExplanationSize 30;

setAnnotationSize 20;

setFontIncAnnot 0;

setFontIncExp 0;

setSize 1913 889;

setLocation -8 0;

setFont SanSerif;

setFontSize 10;

getToolPalette;

tell It;

setTool mesquite.trees.BasicTreeWindowMaker.BasicTreeWindow.arrow;

endTell;

getTreeDrawCoordinator #mesquite.trees.BasicTreeDrawCoordinator.BasicTreeDrawCoordinator;

tell It;

suppress;

setTreeDrawer #mesquite.trees.SquareTree.SquareTree;

tell It;

setNodeLocs #mesquite.trees.NodeLocsStandard.NodeLocsStandard;

tell It;

branchLengthsToggle off;

toggleScale on;

toggleBroadScale off;

toggleCenter on;

toggleEven on;

setFixedTaxonDistance 0;

endTell;

setEdgeWidth 6;

orientUp;

setCornerMode Right_Angle 50;

endTell;

setBackground White;

setBranchColor Black;

showNodeNumbers off;

showBranchColors on;

labelBranchLengths off;

centerBrLenLabels on;

showBrLensUnspecified on;

showBrLenLabelsOnTerminals on;

setBrLenLabelColor 0 0 255;

setNumBrLenDecimals 6;

desuppress;

getEmployee #mesquite.trees.BasicDrawTaxonNames.BasicDrawTaxonNames;

tell It;

setColor Black;

toggleColorPartition on;

toggleColorAssigned off;

toggleShadePartition off;

toggleShowFootnotes on;

toggleNodeLabels on;

toggleCenterNodeNames off;

toggleShowNames on;

namesAngle ?;

endTell;

endTell;

setTreeNumber 1;

setTree '(1,(2,(3,(4,(5,((100,101),(7,(8,9,10,((((11,77),(61,62)),64),(((((12,73),(69,(70,(71,72)))),65),((37,57),(38,39))),((90,((98,99),((95,(96,97)),(91,(92,(93,94)))))),(13,(14,((87,(88,89)),(((15,43),40),(((81,(82,(83,(84,85,86)))),(20,21)),(((((79,80),48),46),(16,(17,18))),(31,(32,36,(27,(52,(53,(54,(33,(34,35)))))),(6,(((28,(29,30)),((19,22),((25,26),(23,24)))),((78,(74,(75,76))),(((((59,60),50),45),(51,(56,(55,58)))),(((41,42),44),((63,(66,(67,68))),(47,49))))))))))))))))))))))))));';

setDrawingSizeMode 0;

toggleLegendFloat on;

scale 0;

toggleTextOnTree off;

togglePrintName on;

showWindow;

endTell;

desuppressEPCResponse;

getEmployee #mesquite.trees.ColorBranches.ColorBranches;

tell It;

setColor Red;

removeColor off;

endTell;

getEmployee #mesquite.ornamental.BranchNotes.BranchNotes;

tell It;

setAlwaysOn off;

endTell;

getEmployee #mesquite.ornamental.ColorTreeByPartition.ColorTreeByPartition;

tell It;

colorByPartition off;

endTell;

getEmployee #mesquite.ornamental.DrawTreeAssocDoubles.DrawTreeAssocDoubles;

tell It;

setOn on;

toggleShow consensusFrequency;

toggleShow posteriorProbability;

toggleShow bootstrapFrequency;

toggleShow consensusFrequency;

toggleShow posteriorProbability;

toggleShow bootstrapFrequency;

setDigits 4;

setThreshold ?;

writeAsPercentage off;

toggleCentred on;

toggleHorizontal on;

toggleWhiteEdges on;

toggleShowOnTerminals on;

setFontSize 10;

setOffset 0 0;

endTell;

getEmployee #mesquite.ornamental.DrawTreeAssocStrings.DrawTreeAssocStrings;

tell It;

setOn on;

toggleCentred on;

toggleHorizontal on;

setFontSize 10;

setOffset 0 0;

toggleShowOnTerminals on;

endTell;

getEmployee #mesquite.trees.TreeInfoValues.TreeInfoValues;

tell It;

panelOpen false;

endTell;

endTell;

endTell;

getEmployee #mesquite.charMatrices.BasicDataWindowCoord.BasicDataWindowCoord;

tell It;

showDataWindow #1104077935316279450 #mesquite.charMatrices.BasicDataWindowMaker.BasicDataWindowMaker;

tell It;

getWindow;

tell It;

getTable;

tell It;

rowNamesWidth 212;

endTell;

setExplanationSize 30;

setAnnotationSize 40;

setFontIncAnnot 0;

setFontIncExp 0;

setSize 1913 869;

setLocation -8 0;

setFont SanSerif;

setFontSize 12;

getToolPalette;

tell It;

setTool mesquite.charMatrices.BasicDataWindowMaker.BasicDataWindow.ibeam;

endTell;

setActive;

setTool mesquite.charMatrices.BasicDataWindowMaker.BasicDataWindow.ibeam;

colorCells #mesquite.charMatrices.ColorByState.ColorByState;

tell It;

setStateLimit 9;

toggleUniformMaximum on;

endTell;

colorRowNames #mesquite.charMatrices.TaxonGroupColor.TaxonGroupColor;

colorColumnNames #mesquite.charMatrices.ColorByFootnote.ColorByFootnote;

colorText #mesquite.charMatrices.NoColor.NoColor;

setBackground White;

toggleShowNames off;

toggleShowTaxonNames on;

toggleTight off;

toggleThinRows off;

toggleShowChanges on;

toggleSeparateLines off;

toggleShowStates on;

toggleAutoWCharNames on;

toggleAutoTaxonNames off;

toggleShowDefaultCharNames off;

toggleConstrainCW on;

setColumnWidth 16;

toggleBirdsEye off;

toggleShowPaleGrid off;

toggleShowPaleCellColors off;

toggleShowPaleExcluded off;

togglePaleInapplicable off;

toggleShowBoldCellText off;

toggleAllowAutosize on;

toggleColorsPanel off;

toggleDiagonal on;

setDiagonalHeight 80;

toggleLinkedScrolling on;

toggleScrollLinkedTables off;

endTell;

showWindow;

getEmployee #mesquite.charMatrices.AlterData.AlterData;

tell It;

toggleBySubmenus off;

endTell;

getEmployee #mesquite.charMatrices.ColorCells.ColorCells;

tell It;

setColor Orange;

removeColor off;

endTell;

getEmployee #mesquite.categ.StateNamesEditor.StateNamesEditor;

tell It;

makeWindow;

tell It;

getTable;

tell It;

rowNamesWidth 255;

endTell;

setExplanationSize 30;

setAnnotationSize 20;

setFontIncAnnot 0;

setFontIncExp 0;

setSize 1913 889;

setLocation -8 0;

setFont SanSerif;

setFontSize 10;

getToolPalette;

tell It;

setTool mesquite.categ.StateNamesEditor.StateNamesWindow.ibeam;

endTell;

rowsAreCharacters on;

toggleConstrainChar on;

toggleConstrainCharNum 3;

togglePanel off;

toggleSummaryPanel off;

endTell;

showWindow;

endTell;

getEmployee #mesquite.categ.StateNamesStrip.StateNamesStrip;

tell It;

showStrip off;

endTell;

getEmployee #mesquite.charMatrices.AnnotPanel.AnnotPanel;

tell It;

togglePanel off;

endTell;

getEmployee #mesquite.charMatrices.CharReferenceStrip.CharReferenceStrip;

tell It;

showStrip off;

endTell;

getEmployee #mesquite.charMatrices.QuickKeySelector.QuickKeySelector;

tell It;

autotabOff;

endTell;

getEmployee #mesquite.charMatrices.SelSummaryStrip.SelSummaryStrip;

tell It;

showStrip off;

endTell;

getEmployee #mesquite.categ.SmallStateNamesEditor.SmallStateNamesEditor;

tell It;

panelOpen true;

endTell;

endTell;

endTell;

getEmployee #mesquite.charMatrices.ManageCharacters.ManageCharacters;

tell It;

showCharacters #1104077935316279450 #mesquite.lists.CharacterList.CharacterList;

tell It;

setData 0;

getWindow;

tell It;

useTargetValue off;

setTargetValue ;

newAssistant #mesquite.lists.DefaultCharOrder.DefaultCharOrder;

newAssistant #mesquite.lists.CharListInclusion.CharListInclusion;

newAssistant #mesquite.lists.CharListPartition.CharListPartition;

newAssistant #mesquite.charMatrices.SortsInCharList.SortsInCharList;

newAssistant #mesquite.parsimony.CharListParsModels.CharListParsModels;

getTable;

tell It;

columnWidth 1 70;

columnWidth 2 145;

columnWidth 4 100;

endTell;

setExplanationSize 30;

setAnnotationSize 20;

setFontIncAnnot 0;

setFontIncExp 0;

setSize 1913 889;

setLocation -8 0;

setFont SanSerif;

setFontSize 10;

getToolPalette;

tell It;

setTool mesquite.lists.CharacterList.CharacterListWindow.ibeam;

endTell;

endTell;

showWindow;

getEmployee #mesquite.lists.CharListAnnotPanel.CharListAnnotPanel;

tell It;

togglePanel off;

endTell;

endTell;

endTell;

endTell;

end;
